# Salmonids elicit an acute behavioral response to heterothermal environments

**DOI:** 10.7554/eLife.99126

**Published:** 2026-10-14

**Authors:** Robert Naudascher, Stefano Brizzolara, Jonasz Slomka, Robert M Boes, Markus Holzner, Luiz GM Silva, Roman Stocker

**Affiliations:** 1 Institute of Environmental Engineering, Department of Civil, Environmental and Geomatic Engineering, ETH Zurich Zurich Switzerland; 2 Swiss Federal Institute of Forest, Snow and Landscape Research WSL Birmensdorf Switzerland; 3 Laboratory of Hydraulics, Hydrology and Glaciology (VAW), Swiss Federal Institute of Technology Zurich Zurich Switzerland; Emory University United States; CNRS France

**Keywords:** *Salmo trutta*, Salmonids, Thermoregulation, Thermopeaking, Thermal Interfaces, Ecohydraulics, Brown Trout

## Abstract

Most fish species are ectothermic and rely on behavioral strategies to control their body temperature in heterothermal environments. Both thermotaxis and thermokinesis have been suggested as important underlying mechanisms. However, to what extent these behaviors allow fish to respond to rapid (timescales of minutes) and strong thermal disturbances, like those caused by anthropogenic water releases into natural freshwater systems, is poorly understood. Here, we quantify this response for a salmonid species with a novel laboratory approach coupled with image-based tracking. We exposed brown trout parr (*Salmo trutta*), acclimated to 12 °C, to rapidly forming cold- and warm-water interfaces with temperatures ranging from 4 to 20 °C. We found that fish actively avoided colder water (≤8 °C) through a rapid response that combined thermotaxis and thermokinesis. In contrast, fish did not avoid warmer water and frequently crossed interfaces having temperature contrasts of up to 8 °C. This study shows that brown trout parr swiftly deploy multiple behavioral strategies to minimize exposure to cold water and take advantage of warm water, illustrating their capability to cope with rapidly occurring thermal alterations.

## Introduction

Water temperature constitutes a key physical property of riverine ecosystems. It has been described as the ‘abiotic master factor’ for fishes (Beitinger and Fitzpatrick, 1979; Brett, 1971), strongly affecting their energy balance and behavior (Volkoff and Rønnestad, 2020; Takatsu et al., 2022). In natural habitats, temperature may change on a variety of spatial and temporal scales. Thermal gradients occur at river confluences, at locations where rivers flow into lakes, and within vertically stratified lakes. As most fish species are ectotherms, their body temperature changes according to the temperature of the water they occupy (Haesemeyer, 2020). Because temperature influences the rates of most physiological processes, rapid warming or cooling can affect fish performance traits, including metabolic rates, swimming ability, and thermal tolerance (Jutfelt et al., 2024).

Because they lack physiological means to regulate body temperature, fish have two main choices when exposed to heterothermal environments (i.e. environments in which temperature varies over space and/or time): acclimate physiologically to changes in local temperature (physiological plasticity; Fry, 1967; Morgan et al., 2022) or adopt specific behaviors to regulate their body temperature (behavioral thermoregulation; Haesemeyer, 2020; Prosser and Nelson, 1981). Behavioral thermoregulation allows fish to thrive in environments where temperature fluctuates, by maximizing the time spent in areas where temperature is within their optimum performance range and by avoiding temperature extremes. While fish may generally adjust to gradual temperature changes through physiological plasticity, the risk of stress increases for more rapid temperature changes (Donaldson et al., 2008) and in this situation, behavioral thermoregulation may be of greater importance.

Fish were found to actively seek optimal temperatures in their environment through movement behavior 50 years ago (Neill et al., 1972). Recent experiments to determine the mechanisms underlying behavioral thermoregulation have identified both thermokinetic and thermotactic components (Haesemeyer et al., 2015; Haesemeyer et al., 2018). Thermokinesis refers to a change in the rate of movement of fish in response to temperature. One explanation for thermokinesis is that a change in body temperature impacts the metabolic rate, which in turn affects movement activity. In this scenario, thermal sensing is not required. For instance, zebrafish larvae (*Danio rerio*) increase their turn frequency, turn amplitude, and swimming speed when water temperature is increased from 18 °C to 32 °C (Le Goc et al., 2021). This thermokinetic behavior can explain how zebrafish larvae avoid warmer water, but not how they avoid colder water.

Thermotaxis, in contrast, is the directed movement along a temperature gradient, toward warmer or colder water, and requires the ability to sense a temperature gradient (Haesemeyer et al., 2015; Haesemeyer et al., 2018). Fish may achieve thermotaxis either by sensing a thermal gradient spatially along their body or temporally over a given time window as they swim. The response to a thermal gradient can then be either deterministic, through directed movement along the gradient, or biased, for example, through the increase in the turning rate when moving down the gradient, analogous to bacterial chemotaxis (Keegstra et al., 2022; Stocker and Seymour, 2012). Thermotaxis has been observed in a number of vertebrate organisms (Giacometti et al., 2021; Ye et al., 2021; Leonard, 1974). While the thermal preference of fish is a well-established field of research, very few quantitative studies of behavioral mechanisms allowing fish to seek their preferendum (i.e. thermotaxis) have been conducted in fish. Among these is work showing that larval zebrafish integrate thermal information over a 500 ms time window (Haesemeyer, 2020) and bias the magnitude and direction of their turns to favor movement toward colder regions.

Past studies have investigated the long-term (hours) behavioral response of fish to stationary thermal gradients. In contrast, very little is known about the thermoregulatory behavior in response to the exposure to sharp, rapidly occurring thermal gradients associated, for example with anthropogenic disturbances (Haesemeyer et al., 2015). Here, we report experiments to quantify the thermoregulatory behavior of a salmonid in its early life-stages in response to the rapid formation of sharp vertical temperature gradients generated using a lock-release approach. In particular, we identified a knowledge gap associated with the rapid intrusion of cold and warm water into the fish’s habituated surrounding. Thereby, we advance our understanding of thermoregulatory behavior in vertically stratified heterothermal environments. Salmonids are among the most common and well-studied families of freshwater fish in the northern hemisphere, yet little is known about their thermoregulatory behavior (McCullough et al., 2009). All salmonids spawn in the shallow gravel beds of freshwater headstreams and spend most of their juvenile stage in rivers and small creeks, where temperature regimes are often strongly affected by human activities (Antonetti et al., 2023). Due to their small body size, the early life-stages of fish gain or lose heat rapidly, making them particularly sensitive to thermal disturbances (Stevens and Sutterlin, 1976).

The natural thermal regime of rivers can be significantly altered by human activities (Vanzo et al., 2016), including the operation of storage hydropower (i.e. induced thermopeaking; Antonetti et al., 2023; Zolezzi et al., 2011), the use of water for cooling in thermal power plants (Prats et al., 2010), flow regulation at artificial barriers (Dickson et al., 2012; Olden and Naiman, 2010), urbanization (Nelson et al., 2021), and climate change (Barbarossa et al., 2021; Collas et al., 2019). If the local water temperature changes too rapidly, this might trigger an avoidance response, driving fish to abandon habitats (McCullough et al., 2009). For example, recent observations of the impact of thermopeaking show that a sudden decrease in water temperature (by 2.5 °C in 10 min) led to significantly higher downstream drift of juvenile European grayling (*Thymallus thymallus*) than during events with increasing water temperature (Mameri et al., 2023). However, research on this topic remains scarce (Moreira et al., 2019), and the mechanisms underlying rapid behavioral thermoregulation have remained unclear.

Human-induced thermal alterations in freshwater systems can be unnaturally rapid and strong (Zolezzi et al., 2011). For example, the water temperature downstream of hydropower facilities can decrease by up to 6 °C within minutes in spring and summer, and increase strongly (up to 4 °C) in winter (Zolezzi et al., 2011). During spring and summer, cold thermopeaking may cause significant mortality, as it coincides with the presence of early life stages of salmonids and cyprinids (Auer et al., 2023). Rapid thermal changes (multiple degrees Celsius over minutes) are likely challenging for fish, partly because their natural occurrence over evolutionary timescales might have been rare. For instance, if they are unable to relocate upon being exposed to cold water, fish can become cold-shocked, a lethargic state of slowed metabolism, reduced oxygen uptake, and muscle stiffness, resulting in reduced swimming activity and increased risk of predation (Donaldson et al., 2008).

The primary aim of our study was to quantify how brown trout parr (*Salmo trutta*), hereafter trout, respond to rapid (i.e. over timescales $\boldsymbol{\tau }_{\mathrm{PHYS}}$ of minutes) thermal disturbances. To mimic the rapid release of thermal effluent into a freshwater system, we developed a laboratory setup that uses the lock-release of a gravity current to rapidly expose fish acclimated to 12° (their assumed thermal preferendum) to a warmer or colder body of water. By continuously tracking individual fish, we quantified their response to incoming water of different temperatures ($T_{\mathrm{incoming}} = {4-20\,}^{\circ} {\rm C}$). We hypothesized that after rapid exposure, fish would avoid the incoming colder or warmer water across all treatments and that they would do so through a combination of thermokinesis and thermotaxis. To test this hypothesis, we developed a novel imaging approach that allowed simultaneous tracking of the thermal interface and the swimming behavior of individual fish at high spatio-temporal resolution. Our results indicate that fish use both thermokinetic and thermotactic behaviors to rapidly and effectively avoid incoming cold water, whereas they perform frequent excursions into incoming warm water, possibly as a strategy to gradually elevate their body temperature.

## Results

**Figure 1.**
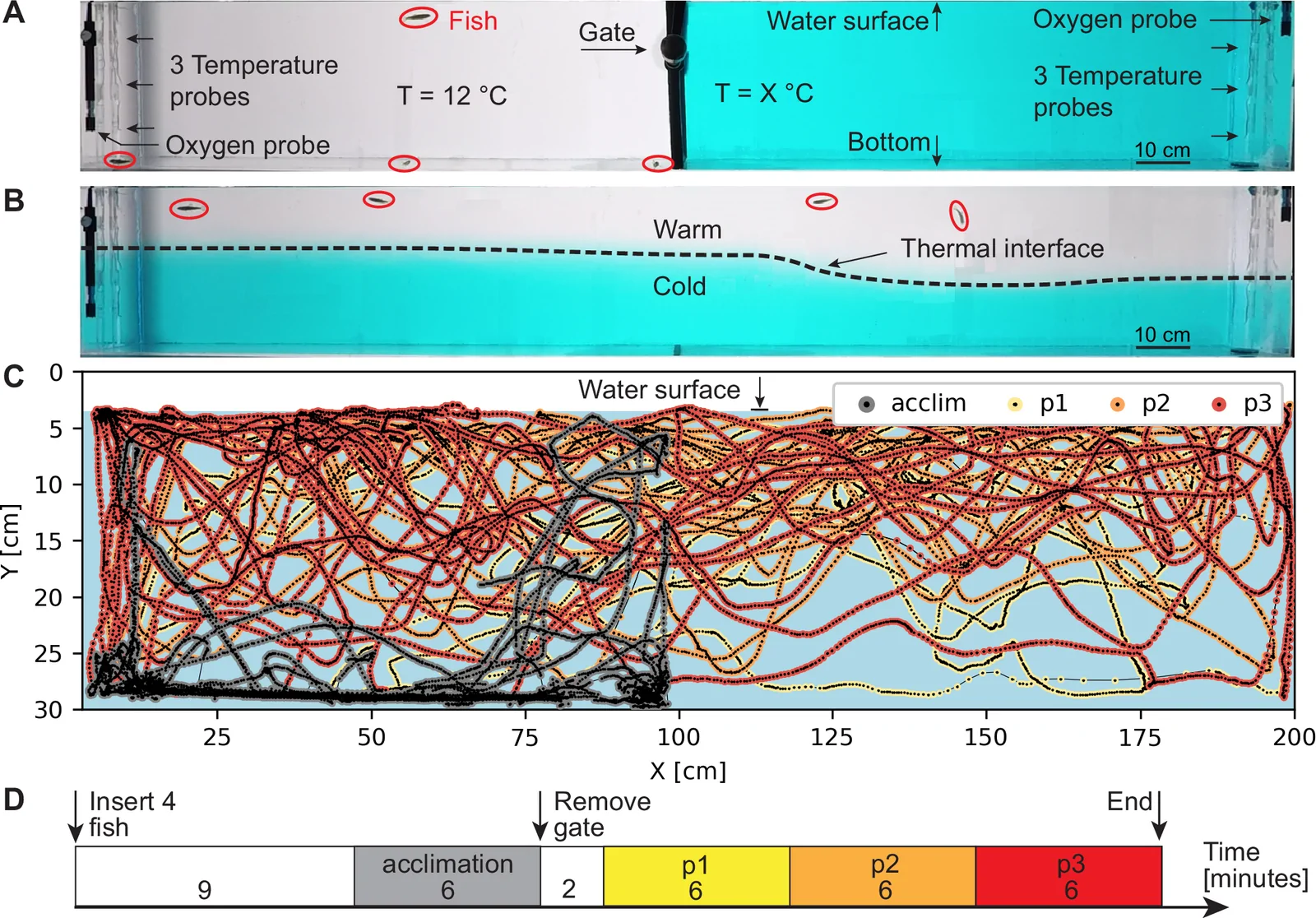
Lock-exchange tank used to expose fish to an acute heterothermal environment. (**A**) Side-view of the experimental tank during the acclimation phase (15 min), with fish in the left compartment exposed to water at their acclimation temperature (12 °C) separated by a closed gate from the dyed water of a different temperature in the right compartment. Red ellipses indicate the locations of four trout parr tested simultaneously. Fish were tracked continuously based on video recordings of the lateral view of the entire tank (Figure 1—figure supplements 1–3). (**B**) The treatment phase was initiated by rapidly removing the gate, allowing the cold water to slowly intrude below the warmer water (1–2 min), thereby exposing the fish to a heterothermal environment. A sharp thermal interface (dashed line) separated upper, warmer water from lower, colder water. Within the first two minutes, the thermal interface moved laterally and afterwards spanned the entire lateral extent of the tank. Over time, the interface increasingly stabilized around the central depth of the tank (Figure 1—figure supplement 4). The movement of the interface was much slower than fish swimming speeds (Figure 1—figure supplement 4 and Appendix 4—figure 2). The frame used here for illustration was captured in a cold-water treatment, 11 min after gate removal. (**C**) Trajectory of a single fish over 18 min (treatment TR3, $\mathrm{T}_{\mathrm{bottom}} = {6\,}^{\circ} {\rm C}$, $\mathrm{T}_{\mathrm{top}} = {12\,}^{\circ} {\rm C}$). The trajectory is color-coded according to the temporal phase of the experiment (see **D**). (**D**) The different phases of the experiments. After 15 min acclimation, the gate was removed and it took approximately 2 min to establish a horizontal thermal interface spanning the entire tank length. To allow the analysis of temporal trends in fish behavior, the dataset was divided into four periods of equal duration: acclimation, and experimental phases p1–p3. For a list of treatments, see Appendix 1—table 1.

To test the hypothesis that strong thermal gradients trigger an avoidance response in trout, we exposed individuals (in groups of four for each replicate experiment) to heterothermal conditions in a laboratory setup. The setup consisted of a lock-exchange tank initially divided into equal halves containing water at two different temperatures (Figure 1A). To avoid behavioral bias due to visual cues, the tank was shielded with white semi-transparent material (except for the front and the water surface). The experiments were conducted in a dark room, and the tank was illuminated with a light source from behind (Figure 1—figure supplement 1). In the left side of the tank, fish were acclimated for 15 min to water at 12 °C, which was assumed to be close to their thermal preferendum (Reynolds and Casterlin, 1979a; Reynolds and Casterlin, 1979b). This water temperature was also equal to the husbandry tank at our experimental facility. The right side of the tank contained water at a different temperature (colder or warmer), dyed blue (Patent blue V sodium salt, Sigma Aldrich) to allow visualization of the thermal interface (Appendix 1—figure 1). Experiments included four cold treatments (TR1, 10 °C; TR2, 8 °C; TR3, 6 °C; TR4, 4 °C) and four warm treatments (TR6, 14 °C; TR7, 16 °C; TR8, 18 °C; TR9, 20 °C; Appendix 1—table 1). After the 15 min acclimation time, the vertical lock separating the two halves of the tank was rapidly lifted, resulting in a gravity current with warmer water intruding along the top of the tank and colder water intruding in the opposite direction along the bottom. The initial gravity current reached the end-wall of the tank after 1–2 min, creating a sharp thermal interface vertically separating the colder water in the bottom part of the tank ($T_{\mathrm{bottom}}$) from the warmer water in the top part ($T_{\mathrm{top}}$; Figure 1B; Appendix 1—figure 1). Owing to the oscillations triggered by the lock release, the vertical location of the thermal interface was not constant in time (Appendix 13—Videos 1 and 2), but instead oscillated around the half depth of the tank, with oscillations dissipating over time (Figure 1—figure supplement 4).

**Figure 1—figure supplement 1.**
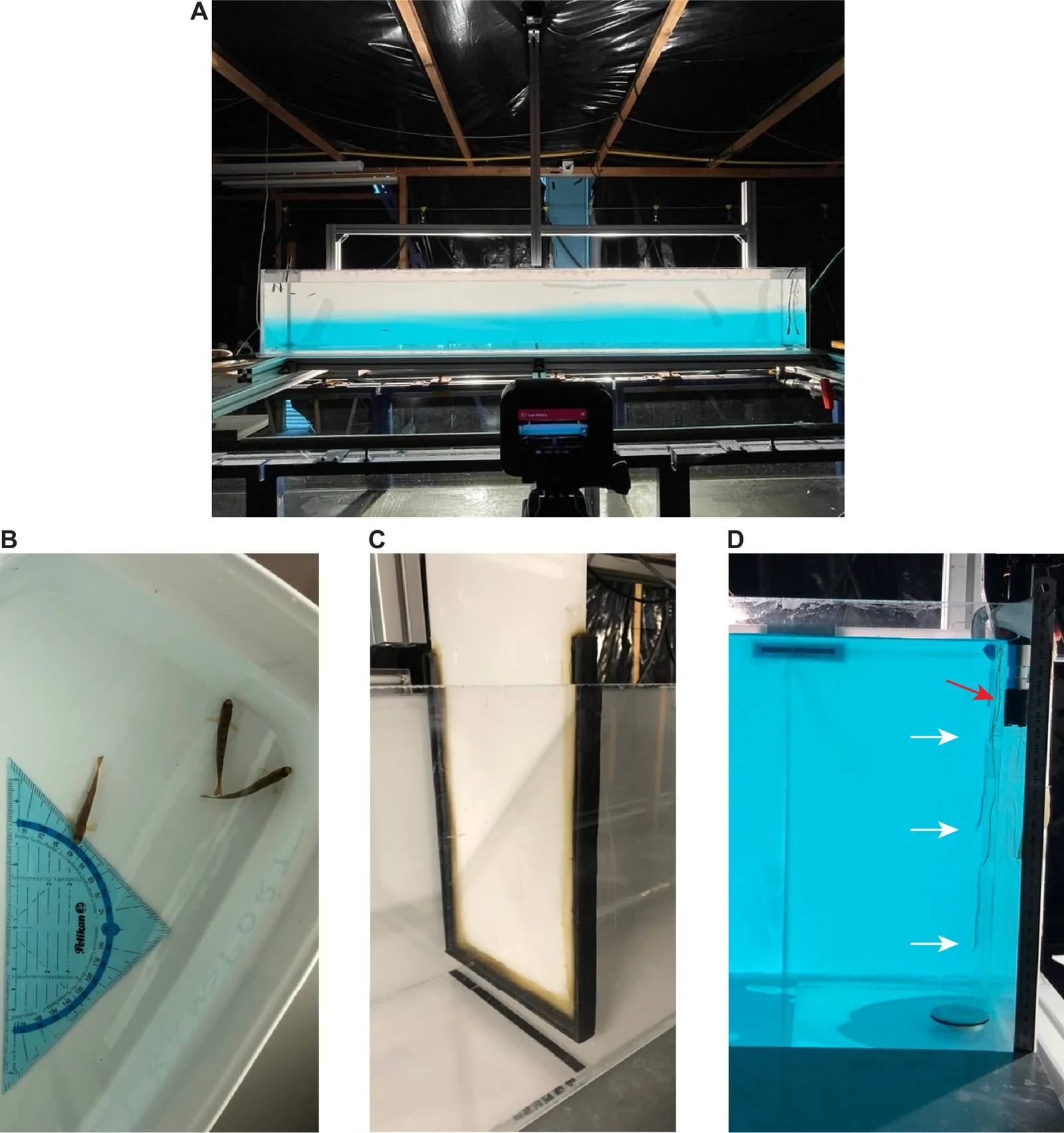
Experimental setup and animals. (**A**) Photograph showing a side-view of the experimental tank in phase 2 (approximately 10 min after removal of the central gate) with four individual fish. The camera used for imaging is visible in the foreground. The dark casing obstructs the entry of natural light. Experimental animals (**B**); central gate to partition the tank; (**C**) right side of the tank with three temperature probes (white arrows) and dissolved oxygen probe (red arrow) (**D**).

**Figure 1—figure supplement 2.**
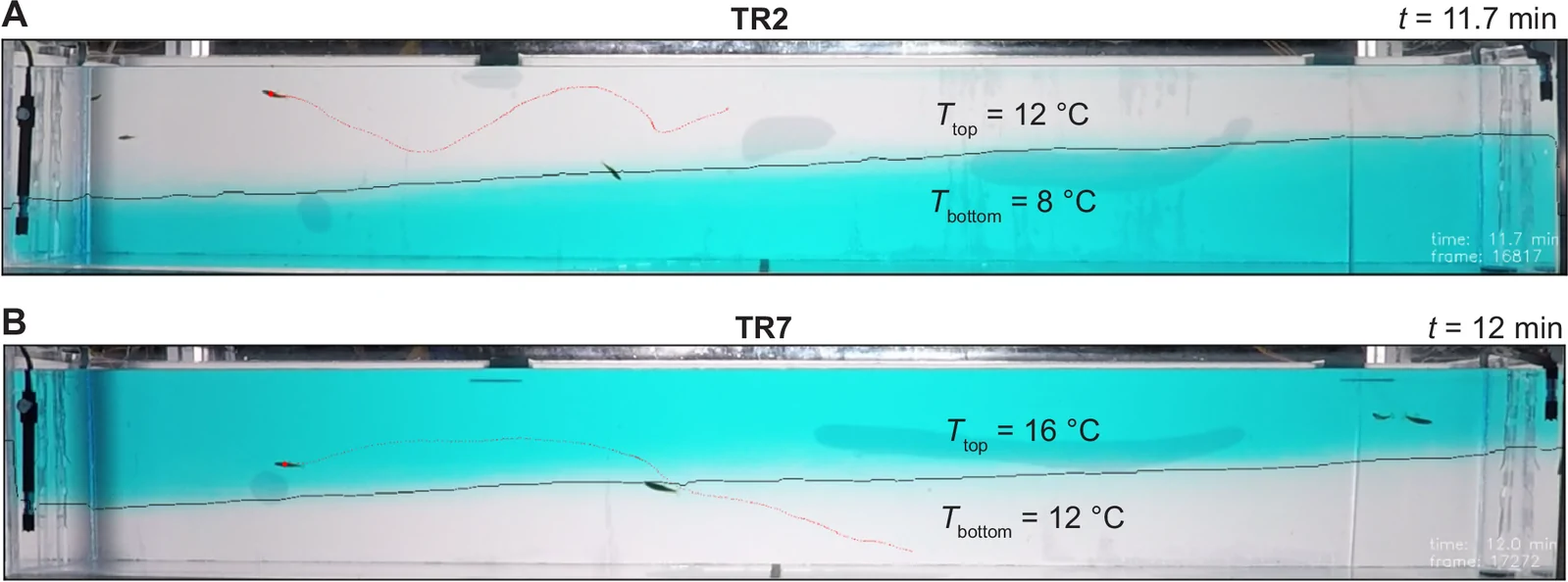
Side view of experimental setup for cold (**A**) and warm (**B**) treatment (TR2 and TR7). The thermal interface line (black) was derived using a custom-made image analysis pipeline.

**Figure 1—figure supplement 3.**
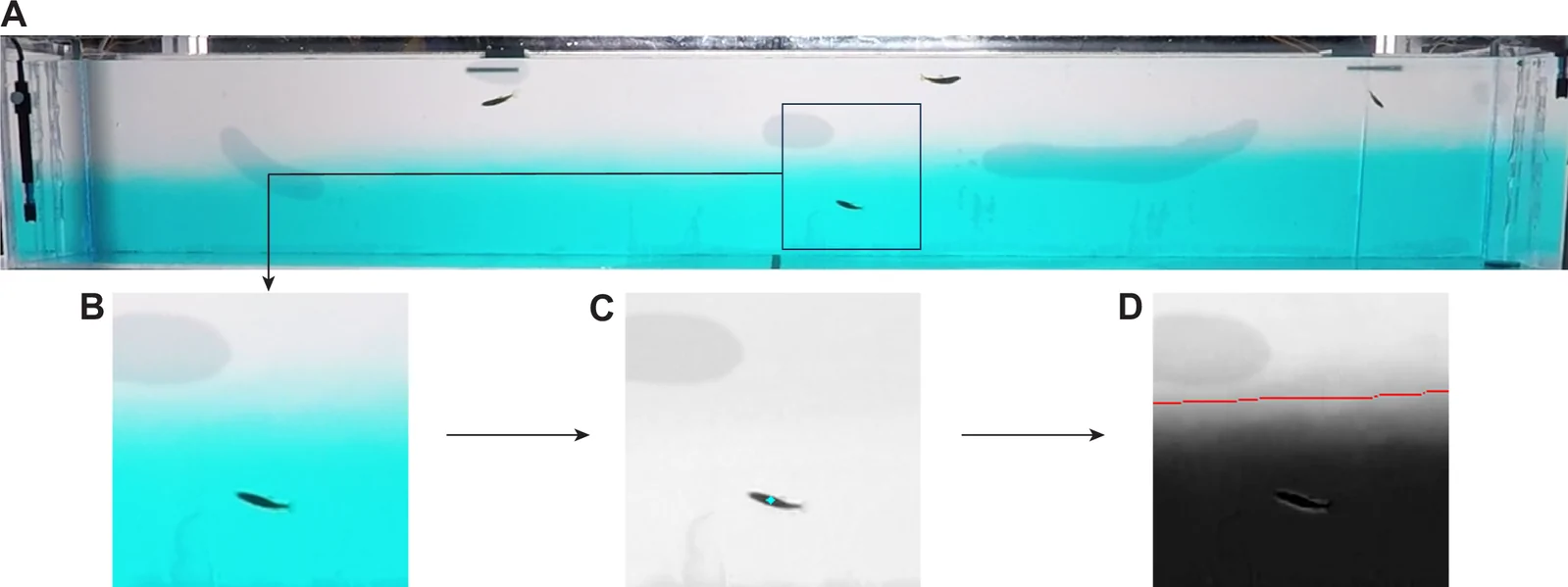
Image processing steps to derive fish position and thermal interface. (**A**) Rotated and cropped RGB image of the side camera (GoPro hero7). (**B**) Subsection of RGB image. (**C**) Red channel. The turquoise dot indicates the fish’s center of gravity for this time instance, derived from tracking in TRex. (**D**) Green channel. Red line indicates the thermal interface, derived by custom-made Python scripts.

**Figure 1—figure supplement 4.**
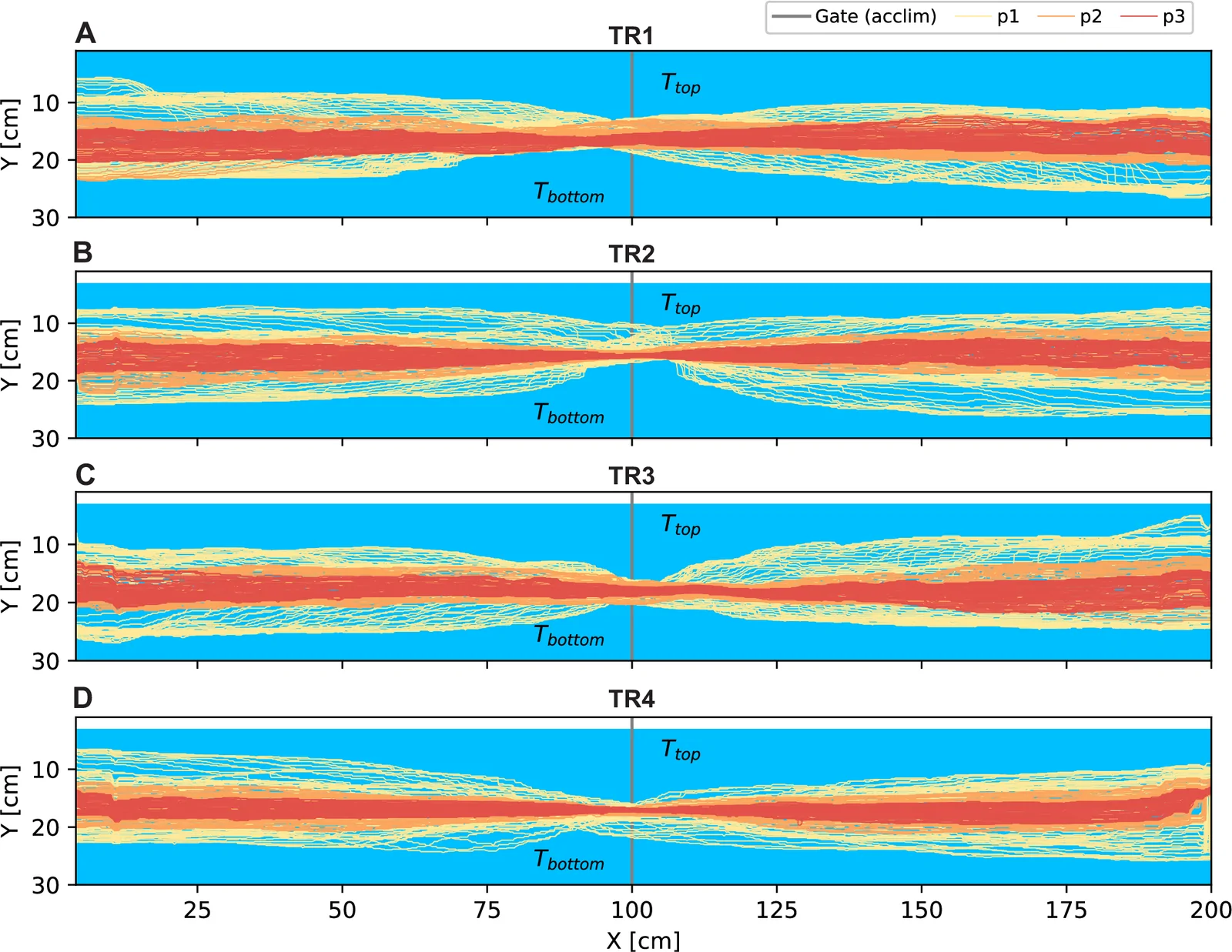
Movement of thermal interface. Transient movement of the thermal interface/line during experimental phases (p1, p2, and p3) in cold water Treatments (TR1-TR4, **A-D**). Colored lines depict time steps of 10 s. The water surface is located at Y = 4 cm. The instantaneous position of the thermal interface is shown for each experimental phase (acclim., p1, p2, and p3) at a reduced temporal resolution of 0.2 Hz (the temporal resolution in the experiments was 24 Hz). The interface was approximated as a line ($\mathrm{X},\mathrm{Y}$) for each frame. During the acclimation period (acclim: grey dots) a sealed gate was located at X = 100 cm. The interface is colored by the respective temporal phase of the experiment (see **A**).

**Figure 2.**
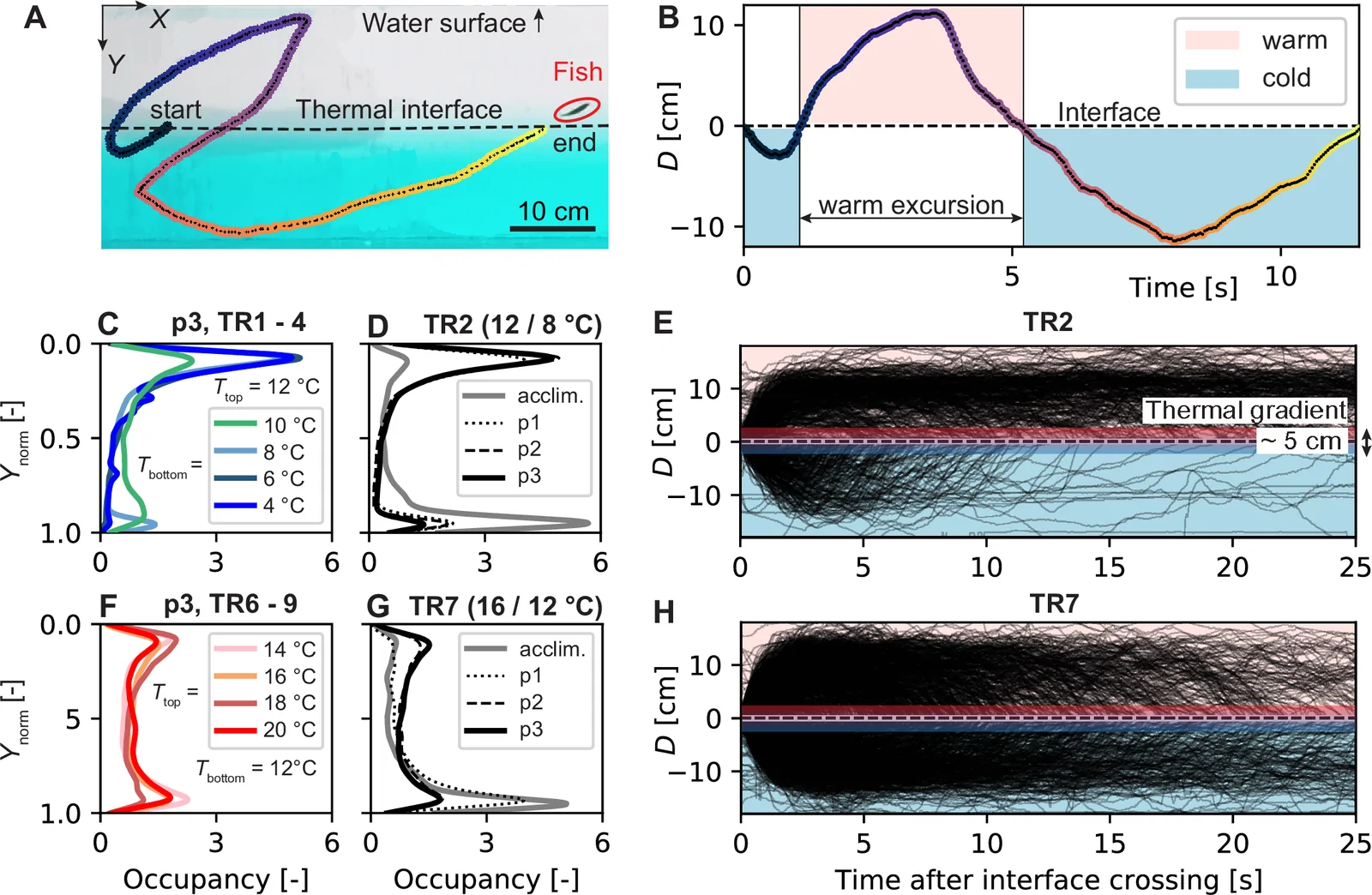
Fish avoid the cold, but not the warm (see also Figure 1—figure supplements 1–3). (**A**) Side-view of the (two-dimensional) swimming trajectory of a fish (colored line) in relation to the thermal interface (dashed black line). Black dots along the trajectory indicate the center of gravity of the fish during an example segment of 12 s. Colors indicate elapsed time, from blue to yellow. (**B**) Definition of warm- and cold-water excursions within a trajectory based on the fish’s instantaneous vertical distance to the thermal interface, $\mathrm{D}\left (\mathrm{t}\right)$. An excursion starts and ends when the fish crosses the thermal interface. (**C, F**) Probability density functions of normalized fish depth $\mathrm{Y}_{\mathrm{norm}}$ during the final 6 min (p3) of the experiments for all cold-water (TR1–TR4, **C**) and all warm-water (TR6–TR9, **F**) treatments. Data were aggregated at the treatment level, hence each line represents data from 20 fish, tested in groups of four individuals in five separate experiments. $\mathrm{T}_{\mathrm{bottom}}$ and $\mathrm{T}_{\mathrm{top}}$ indicate the water temperature below and above the thermal interface. The depth was normalized so that $\mathrm{Y}_{\mathrm{norm}}=0$ is the water surface and $\mathrm{Y}_{\mathrm{norm}}=1$ indicates the bottom of the tank. (**D, G**) Probability density functions of fish depth for TR2 (**D**) and TR7 (**G**), separated according to the four phases of analysis (acclimation phase and experimental phases p1–p3; see Figure 1D). Data were aggregated at the treatment level as in C and F. Data for other treatments are shown in Figure 2—figure supplement 2. (**E, H**) Vertical distance to the interface, (**D**) as a function of time for each cold-water (light blue shaded region) and warm-water excursion (light pink shaded region) for TR2 ($\mathrm{N}=980$) (**E**) and TR7 ($\mathrm{N}=430$) (**H**) aggregated for all three experimental phases (p1–p3). $\mathrm{N}$ is the total number of cold-water excursions performed by the 20 fish. The vertical extent of the thermal gradient thickness is indicated as blue and red (see Methods). Data for other treatments are shown in Appendix 8—figures 2 and 3. For a list of treatments, see Appendix 1—table 1.

The lateral view of the entire tank was continuously recorded for 35 min with a camera at 24 frames/s, starting 4 min after releasing the fish. Using image analysis, we obtained the two-dimensional projection of the trajectory of each fish over the entire duration of the experiment (Figure 1C), while simultaneously tracking the instantaneous location of the thermal interface (Figure 1—figure supplement 2). In this manner, we determined the water temperature experienced by each fish at each point in time. Trajectories were analyzed for the last 6 min of the acclimation period and for the 18-min treatment period, which we divided into three phases of 6 min each (treatment phases p1, p2, and p3; Figure 1D). The depth occupancy was computed from the vertical position of the fish $Y\left (t\right)$ and normalized to the range $Y_{\mathrm{norm}} = 0-1$, with 1 being the maximum depth (28 cm) of the tank. To quantitatively analyze fish movement behavior in relation to the thermal interface, each trajectory was binarized in time based on whether at a given instant a fish was in the warm or the cold water, as determined from the instantaneous vertical distance to the thermal interface, $D\left (t\right)$ (Figure 2A and B*;* Appendix 13—Videos 1 and 3; *Methods*).

**Figure 2—figure supplement 1.**
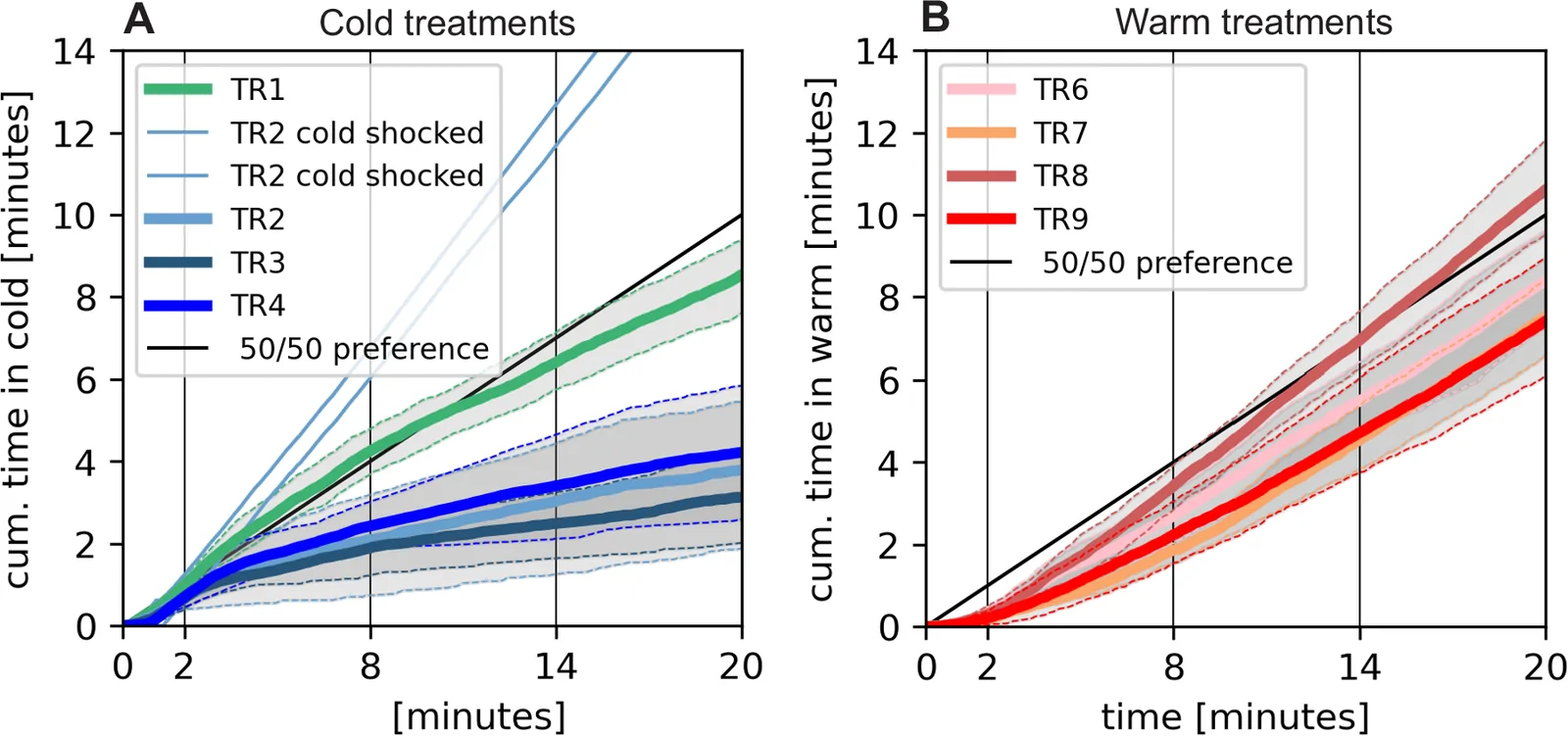
Cumulative time spent in incoming cold water (**A**) and warm water (**B**). Thick lines represent the averaged cumulative time spent in cold water on the replicate level. Thin lines depict the 84th and 16th percentiles across experimental replicates. Two cold shocked individuals (in TR2) were excluded from the average as outliers. Fish displayed opposing responses to incoming warm and cold water over time. Namely, fish decreased the occupancy of cold water, while in warm treatments fish enhanced occupancy of warm water (see also Figure 2—figure supplement 3).

**Figure 2—figure supplement 2.**
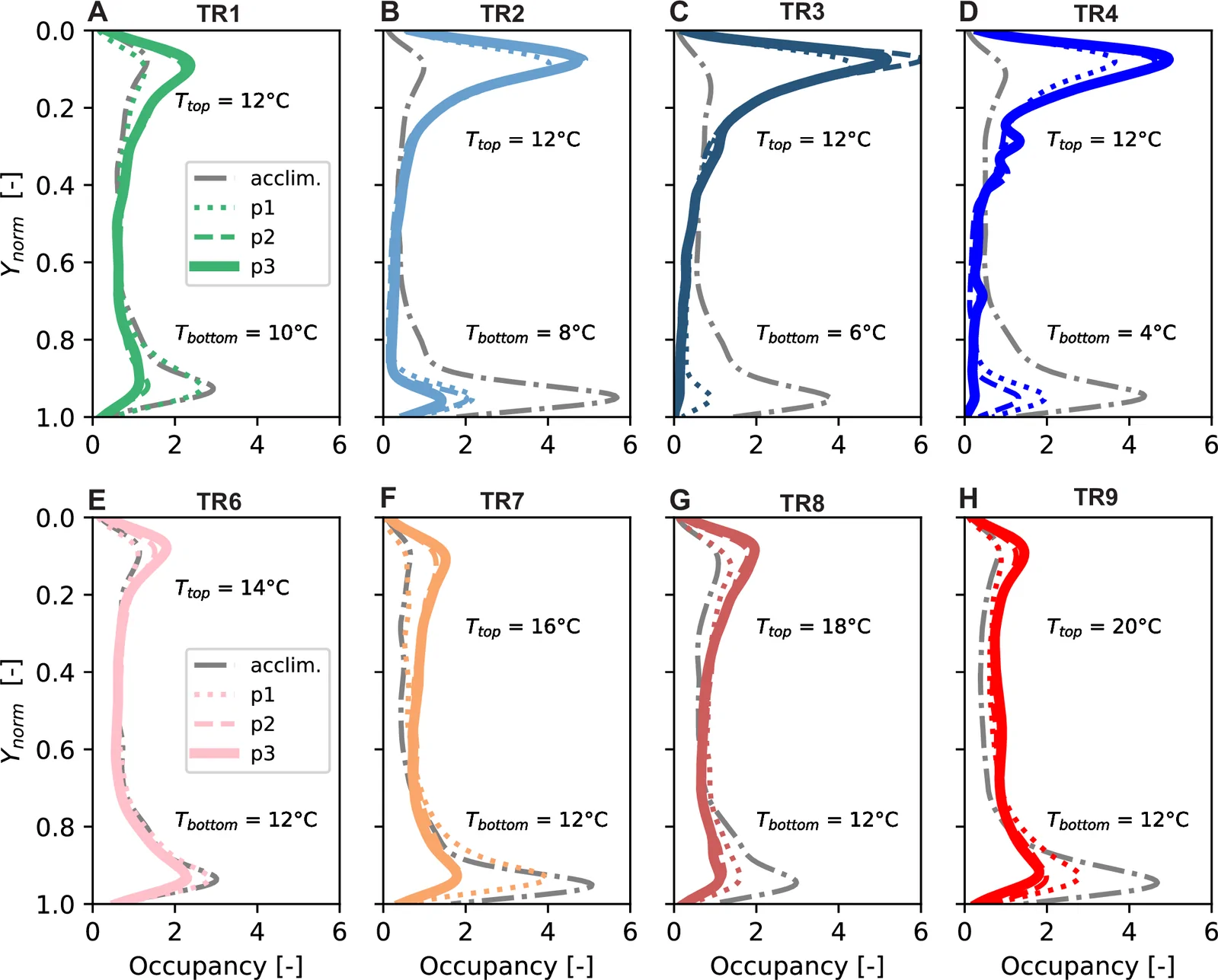
Vertical occupation. Probability density functions of fish depth for all cold-water (TR1–TR4, **A–D**) and all warm-water (TR6–TR9, **E–H**) treatments ($\mathrm{N}=20$ fish in each case), separated according to the four phases of analysis (acclimation, and the three experimental phases p1–p3). The data were aggregated at the treatment level; hence each line represents data from 20 fish, tested in groups of four individuals in five separate experiments. $\mathrm{T}_{\mathrm{b}\mathrm{o}\mathrm{t}\mathrm{t}\mathrm{o}\mathrm{m}}$ and $\mathrm{T}_{\mathrm{t}\mathrm{o}\mathrm{p}}$ indicate the water temperature below and above the thermal interface. The depth was normalized so that $\mathrm{Y}_{\mathrm{norm}}=0$ is the water surface and $\mathrm{Y}_{\mathrm{norm}}=1$ indicates the bottom of the tank.

**Figure 2—figure supplement 3.**
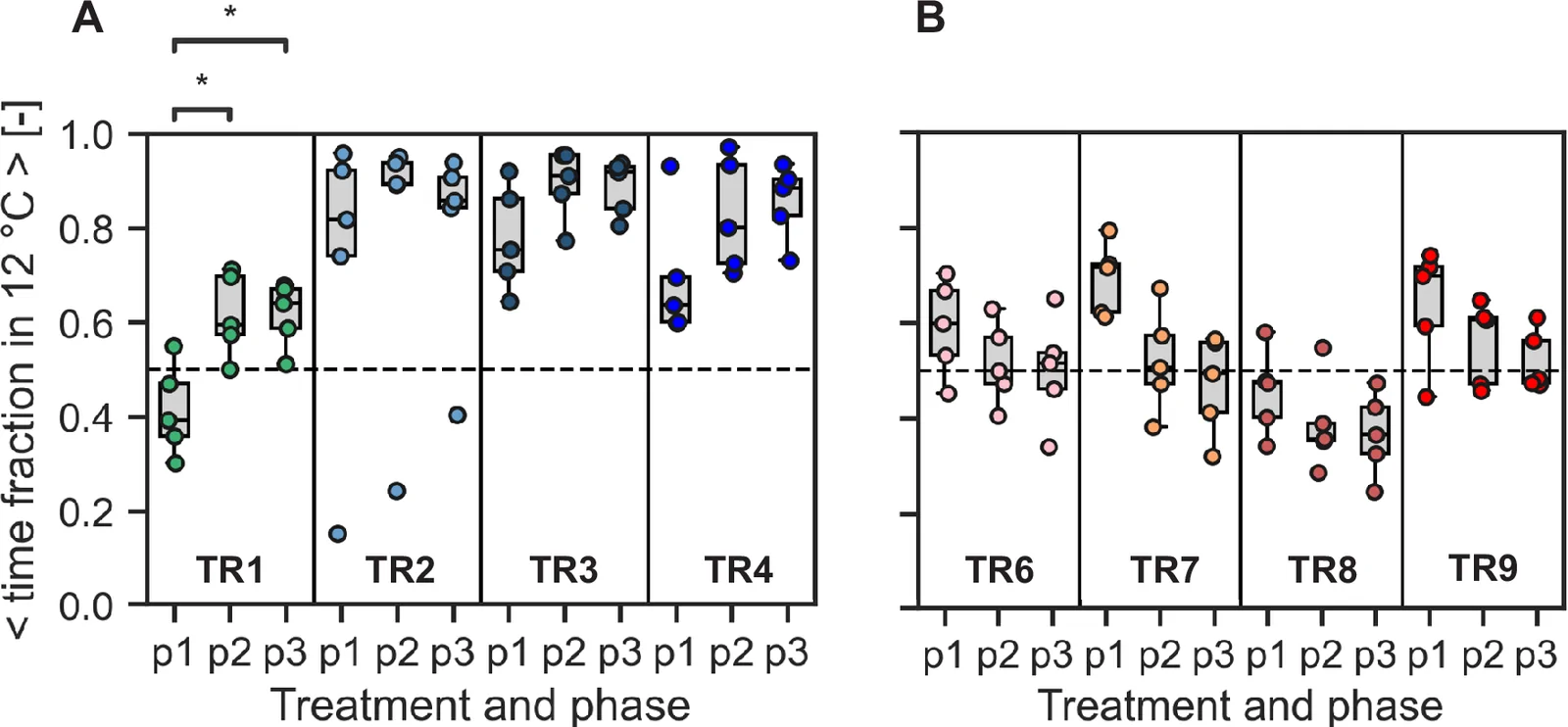
Time spent in acclimation temperature. The average time fraction spent in 12 °C was calculated for each fish and phase (p1, p2, and p3) as $\mathrm{T}_{\mathrm{cold}}/6$ min for incoming cold water (TR1–TR4, **A**) and incoming warm water (TR6–TR9, **B**). Each dot represents the average of this quantity over four individuals that were tested simultaneously within one experimental replicate. The Friedman-test suggests that there are significant temporal differences within TR1 (p = 0.022; Appendix 8—table 1). Significance levels are indicated above the plot (Dunn-test: *: $0.01< \mathrm{p}\leq 0.05$).

### Trout parr avoid cold water by biasing their vertical turns and do not avoid warm water

Fish showed a clear thermotactic avoidance behavior of incoming cold water for $T_{\mathrm{bottom}}\leq {8\,}^{\circ} {\rm C}$. During phase p3 in the three coldest treatments, TR2 ($T_{\mathrm{bottom}}={8\,}^{\circ} {\rm C}$), TR3 ($T_{\mathrm{bottom}}={6 \,}^{\circ} {\rm C}$), and TR4 ($T_{\mathrm{bottom}}={4 \,}^{\circ} {\rm C}$), fish predominantly occupied the upper half of the tank ($Y_{\mathrm{norm}} = 0-0.5$) and avoided the colder water in the bottom half (Figure 2C; see also Appendix 5—figure 1). From phase p1 to p3, fish spent 75–95% of the time above the thermal interface (Figure 2—figure supplement 3A). This represents a substantial change of the preferential depth occupancy compared to the acclimation phase, in which fish spent 50–90% of the time in the lower half of the tank ($Y_{\mathrm{norm}}=0.5-1$; Appendix 5—figure 1). The response was rapid, with avoidance of the cold water being apparent already in phase p1 and persisting through to the end of the experiment (TR2 in Figure 2D; all cold-water treatments in Figure 2—figure supplement 2A-D). In contrast, incoming cold water in TR1 ($T_{\mathrm{bottom}}= {10\,}^{\circ} {\rm C}$) – that is only 2 °C colder than the acclimation temperature – did not trigger an avoidance behavior: the depth occupancy showed a bimodal distribution with peaks in both the warmer and colder water (Figure 2C).

Fish did not avoid warmer water in any of the warm-water treatments (TR6–TR9). Instead, fish transitioned to a bimodal depth occupancy, with comparable time spent above and below the interface (Figure 2F; Figure 2—figure supplement 3B). This bimodal behavior was especially pronounced in the later phases, p2 and p3 (TR7; Figure 2G*;* all warm-water treatments in Figure 2—figure supplement 2E-H), whereas in phase p1, the depth occupancy was similar to that exhibited during the acclimation period, with 50–70% of the time spent in the lower, colder water (Appendix 5—figure 2). Thus, over time, fish increasingly occupied warmer water across all warm-water treatments, reducing the fraction of time spent below the interface during phase p3 to 30–50% (Appendix 9—figure 4B, Appendix 8—figure 4).

Further analysis of the response to cold water treatments revealed a strong asymmetry between warm-water and cold-water excursions, defined as the periods of time during which a fish was above or below the thermal interface, respectively. This asymmetry was present in treatments with $T_{\mathrm{bottom}}\leq {8\,}^{\circ} {\rm C}$: TR2 (Figure 2E), TR3, and TR4 (Appendix 8—figure 2C and D), but not TR1 (Appendix 8—figure 2A). In cold treatments TR2, TR3, and TR4, cold-water excursions were considerably shorter (91–94% shorter than 10 s) than warm-water excursions (38–49% shorter than 10 s; Appendix 8—table 7). In contrast, during all warm treatments, warm and cold excursions had similar durations (Figure 2H, Appendix 8—figure 3).

**Figure 3.**
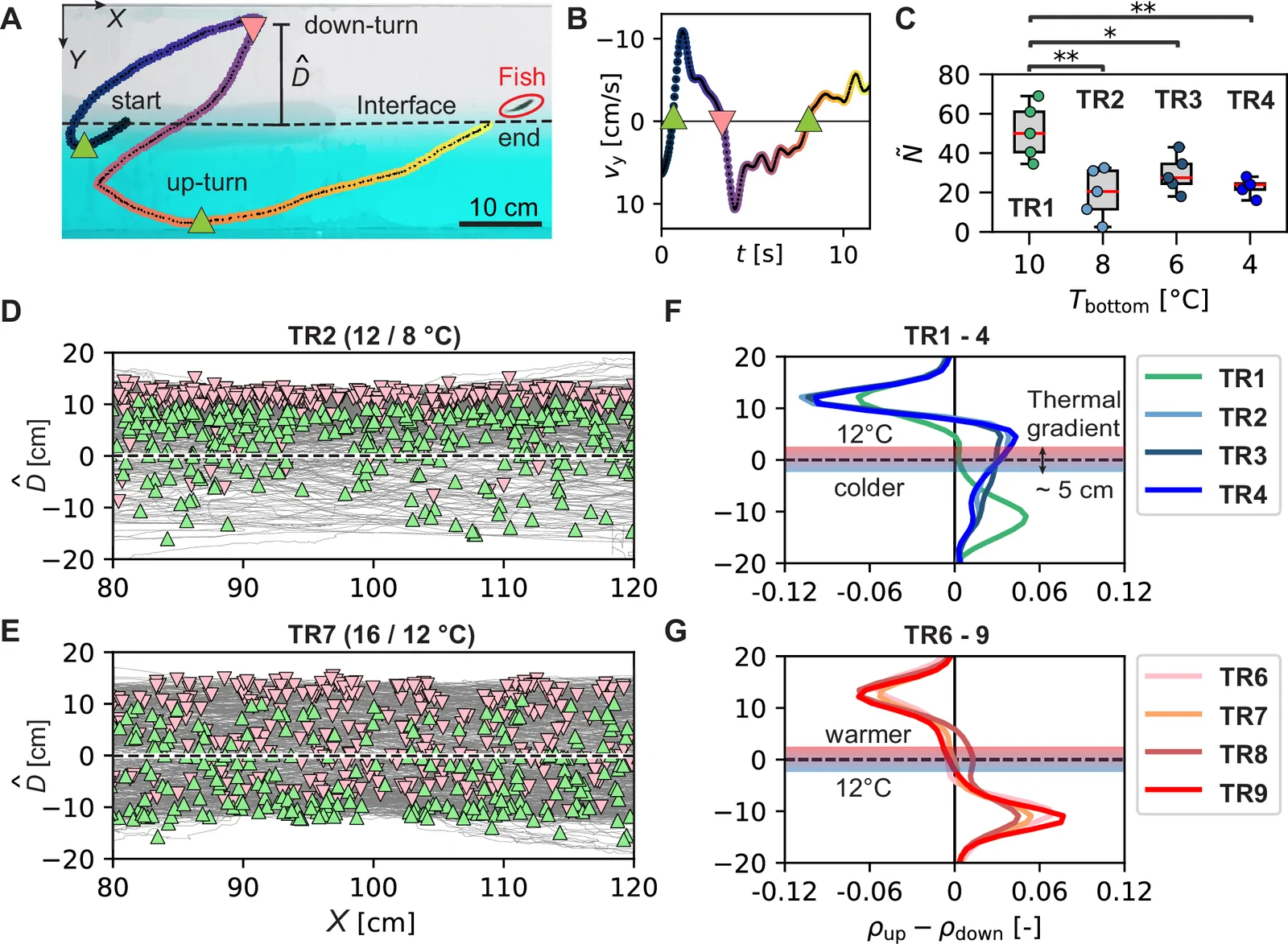
Fish avoid cold water by directional upward turning. (**A**) Example trajectory (12 s) with identified vertical turning points (pink triangles, downward; green triangles, upward). (**B**) Turning points were identified as sign changes of the time series of the vertical velocity component, $\mathrm{v}_{\mathrm{y}}\left (\mathrm{t}\right)$. The same trajectory as in A is shown. (**C**) Boxplots showing the number of cold-water excursions for each cold temperature treatment. Each dot represents the median number of cold-water excursions $\overset{\sim }{\mathrm{N}}$ of four individuals tested simultaneously within one experiment. Red lines indicate the median of five experiments. Whiskers extend to the full range of observations. The temperature treatments TR2 and TR4 differ significantly (ANOVA, p < 0.001), with pairwise t-test results as indicated (* $0.01< {p}\leq 0.05$; ** $0.001< {p}\leq 0.01$, see also Appendix 8—tables 4 and 5). (**D, E**) Swimming trajectories (gray lines) and vertical turning points (color-coded as in panel A) plotted with respect to the vertical distance $\hat {\mathrm{D}}$ to the thermal interface (black dashed line) for cold-water treatment TR2 (**D**) and warm-water treatment TR7 (**E**). The trajectories shown are from the central region ($80< {X}< 120\mathrm{\ }\mathrm{cm}$) of the tank, for 20 fish (5x4 fish per experiment). Turning points were filtered based on a vertical displacement threshold of dz >3 cm to detect turning activity that resulted in significant vertical displacement (Appendix 9—figure 2). Data for other treatments are shown in Appendix 9—figures 3 and 4. (**F, G**) Difference of probability density distributions of upward turns ($\mathrm{\rho }_{\mathrm{up}}$) and downward turns ($\mathrm{\rho }_{\mathrm{down}}$) for the entire width of the tank ($0< {X}< 200\mathrm{\ }\mathrm{cm}$) as a function of the distance from the thermal interface (located at $\hat{\mathrm{D}}\mathrm{\ }=\mathrm{\ }0$), for all cold (**F**) and warm treatments (**G**). The same y-axes as in panels D and E apply. The tank bottom is located at around $-10\mathrm{\ }\mathrm{cm}> \hat{{D}}> -20\mathrm{\ }\mathrm{cm}$, while the water surface is located at around $10\mathrm{\ }\mathrm{cm}< \hat {{D}}< 20\mathrm{\ }\mathrm{cm}$ (the variability being related to the initial period of time in which the thermal interface oscillates). The underlying normalized histograms are in Appendix 9—figure 2C and E. The vertical extent of the thermal gradient thickness is indicated as blue and red (see Methods).

To determine the behavioral mechanism underlying cold-water avoidance, we analyzed the fine-scale features of the fish trajectories as a function of the position of the fish relative to the thermal interface. We identified upward and downward turning events within each trajectory, based on when the vertical velocity component $v_{y}$ changed sign (Figure 3A and B*; Methods*; Appendix 9—figure 1). For each turning event, we computed the corresponding vertical distance $\hat {D}$ to the thermal interface. We also quantified the median number of cold-water excursions $\overset{\sim }{N}$ of the four fish within each experimental replicate. We found that fish reduced their tendency to cross the thermal interface and rapidly redirected their swimming when in the colder water. In cold-water treatments TR2–TR4 ($T_{\mathrm{bottom}}\leq {8\,}^{\circ} {\rm C}$), the median number of interface crossings was significantly lower than in TR1 ($T_{\mathrm{bottom}}= {10 \,}^{\circ} {\rm C}$; ANOVA and post hoc pairwise *t*-tests – p < 0.01, Figure 3C). In TR2-TR4, the probability of upward turning $\rho _{\mathrm{up}}$ exceeded the probability of downward turning $\rho _{\mathrm{down}}$ for the entire region below the interface ($-20\ \mathrm{cm}\ < \ \hat {D}\ < \ 0\ \mathrm{cm}$; Figure 3D and F*,* Appendix 9—figure 3). This difference in probabilities ($\rho _{\mathrm{up}}-\rho _{\mathrm{down}}$) was particularly large in the region immediately above and below the interface ($-5\ \mathrm{cm}< \hat {D}\ < \ 5\ \mathrm{cm}$; Figure 3F) and is a hallmark of a thermotactic behavior.

This thermotactic avoidance behavior was absent in TR1 and all warm treatments, where the probabilities of upward and downward turning were similar ($\rho _{\mathrm{up}}-\rho _{\mathrm{down}}\approx 0$) close to the interface ($-5\ \mathrm{cm}\ < \ \hat {D}\ < \ 5\ \mathrm{cm}$) (Figure 3E and G). In these treatments, the probability of upward turning peaked below the thermal interface, close to the bottom of the tank ($-15\ \mathrm{cm}\ < \ \hat {D}\ < \ -10\ \mathrm{cm}$; Figure 3G), while the probability of downward turning peaked above the thermal interface, close to the water surface ($10\ \mathrm{cm}\ < \ \hat {D}\ < \ 15\ \mathrm{cm}$; Figure 3E, G, Appendix 9—figure 4 for TR6–TR9).

### Fish modulate the duration and depth of cold-water excursions as a function of cold-water temperature

**Figure 4.**
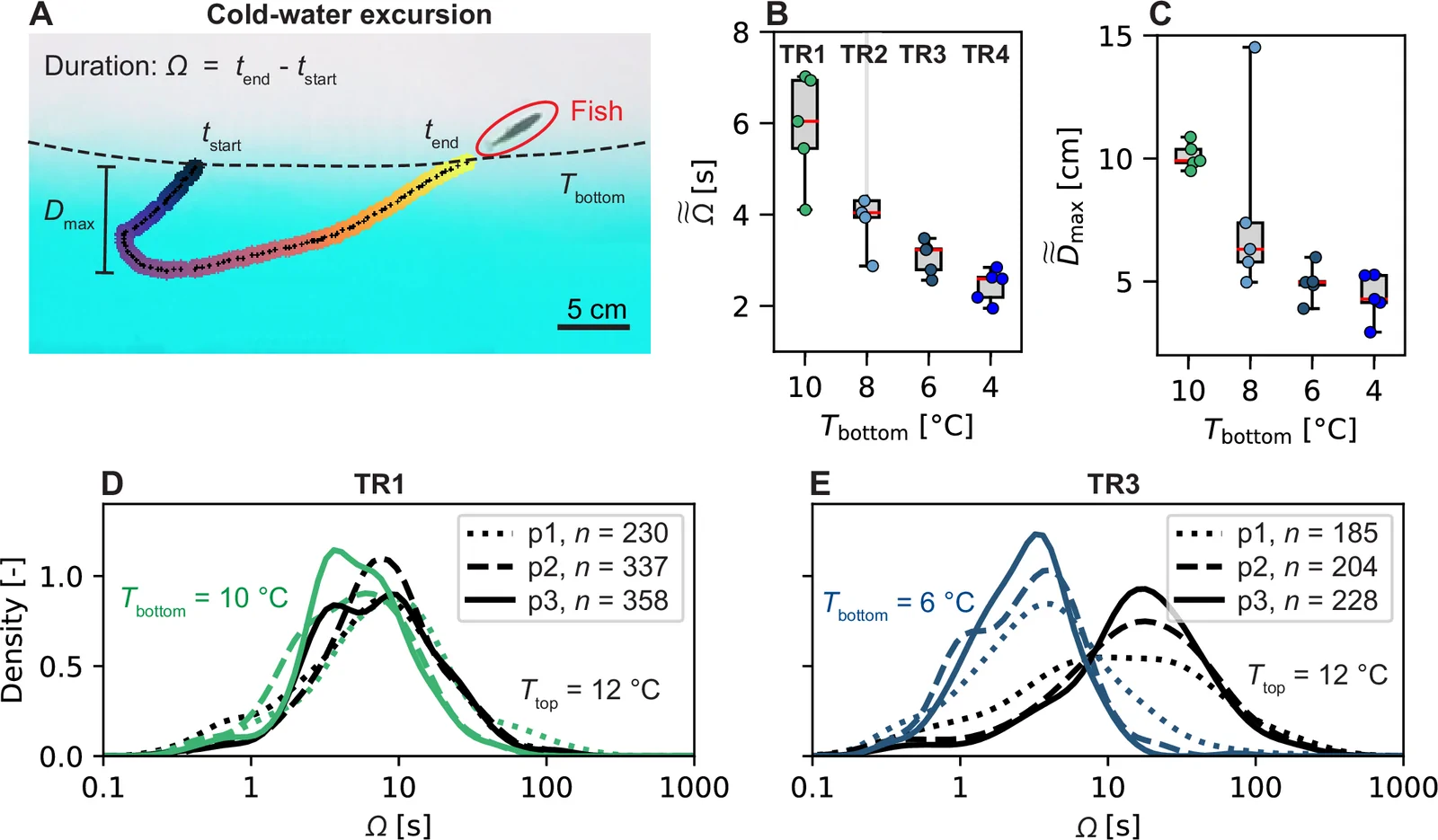
Fish reduce the duration and depth of cold-water excursions in larger measure the colder the water temperature below the thermal interface. (**A**) Fish perform cold-water excursions in which they cross the thermal interface and swim into colder water, then turn upward to return to the warmer water. Individual cold excursions were characterized by their duration, $\mathrm{\Omega }$, and the maximum vertical distance to the thermal interface, $\mathrm{D}_{\max }$. (**B**) Fish exhibit shorter durations of cold-water excursions when encountering lower water temperatures below the interface ($\mathrm{T}_{\mathrm{bottom}}$). The decreasing trend was statistically significant (Mann–Kendall test: for $\overset{\sim }{\overset{\sim }{\mathrm{\Omega }}}$, $\mathrm{p}\mathrm{\, }< \mathrm{\, }0.001$; for $\overset{\sim }{\overset{\sim }{\mathrm{D}}}_{\max }$, $\mathrm{p}\mathrm{\, }< \mathrm{\, }0.001$; Appendix 9—table 1). Boxplots show the median duration of cold-water excursions for fish groups across all phases (p1, p2, and p3) within each treatment. Points represent the median of a single experiment. TR2, one outlier not shown (out of range). (**C**) For colder water temperatures, fish penetrate less deeply into the cold lower water. The decreasing trend was statistically significant (Mann–Kendall test: $\overset{\sim }{\overset{\sim }{\mathrm{D}}}_{\max }$, $\mathrm{p}\mathrm{\, }< \mathrm{\, }0.001$; Appendix 9—table 2). Underlying distributions and histograms are shown in Appendix 8—figure 5. Equivalent plots for warm treatments are shown in Appendix 8—figure 6A and B. (**D, E**) Probability density distributions of the durations $\mathrm{\Omega }$ of warm- and cold-water excursions for TR1 (**D**) and TR3 (**E**), respectively. The distributions of the warm- and cold-water excursion durations become increasingly distinct over exposure time for TR3 but not TR1. Excursions at 12 °C (above the interface) are shown in black, while excursions in the lower, colder water are depicted in the respective color. Treatment phases are indicated by line type (p1–p3). Values of $\mathrm{n}$ indicate the total number of excursions of each type within that phase for 20 fish (as the excursions alternate, $\mathrm{n}$ is equal for cold and warm excursions). Underlying histograms of D and E and other treatments are shown in Figure 4—figure supplements 1 and 2.

In cold treatments, fish did not exclusively stay within the upper warmer water layer but engaged in occasional exploratory behavior of the underlying, colder water. In particular, fish did not reduce the frequency of cold-water excursions over time (Appendix 8—figure 4A). During these excursions, they crossed the interface downwards and thus experienced a sudden decrease in temperature. We found that fish modulated these cold-water excursions as a function of the temperature of the colder water. The duration Ω (s) and depth $D_{\max }$ (the maximum vertical distance below the thermal interface) of cold-water excursions both decreased (Figure 4A) with decreasing temperature of the lower layer ($T_{\mathrm{bottom}}$). This trend was statistically significant for both the median duration $\overset{\sim }{\overset{\sim }{\Omega }}$ (Figure 4B) and the median depth $\overset{\sim }{\overset{\sim }{D}}_{\max }$ (Figure 4C) across treatments (Mann–Kendall test, p<0.001, Appendix 9—tables 1 and 2). As $T_{\mathrm{bottom}}$ decreased from 10 °C to 4 °C (TR1 to TR4), the median excursion duration decreased by 57% (from $\overset{\sim }{\overset{\sim }{\Omega }}= 6\ \mathrm{s}$ to $\overset{\sim }{\overset{\sim }{\Omega }}= 2.6\ \mathrm{s}$) and the median excursion depth decreased by 57% (from $\overset{\sim }{\overset{\sim }{D}}_{\max }= 9.9\ \mathrm{cm}$ to $\overset{\sim }{\overset{\sim }{D}}_{\max }= 4.3\ \mathrm{cm}$, Appendix 9—table 1).

**Figure 4—figure supplement 1.**
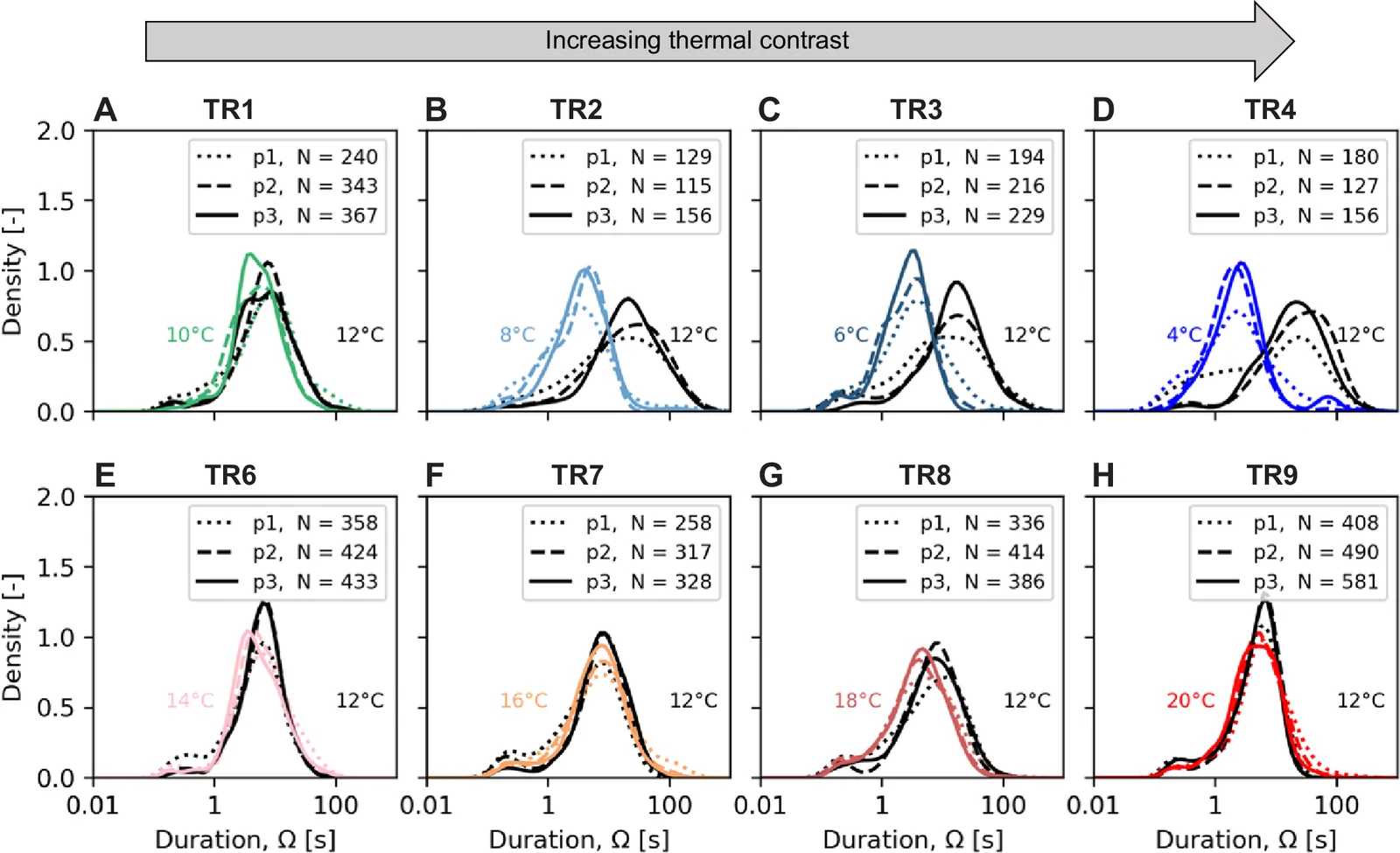
Probability density function of the duration of warm- and cold-water excursions for cold (**A–D**) and warm treatments (**E–H**) and across treatment phases (p1, p2, and p3). As a reference for comparison, black lines depict trajectory excursions in 12 °C. Colored lines show respective distributions in treatment water temperatures below (**A–D**) or above the thermal interface (**E–H**).

**Figure 4—figure supplement 2.**
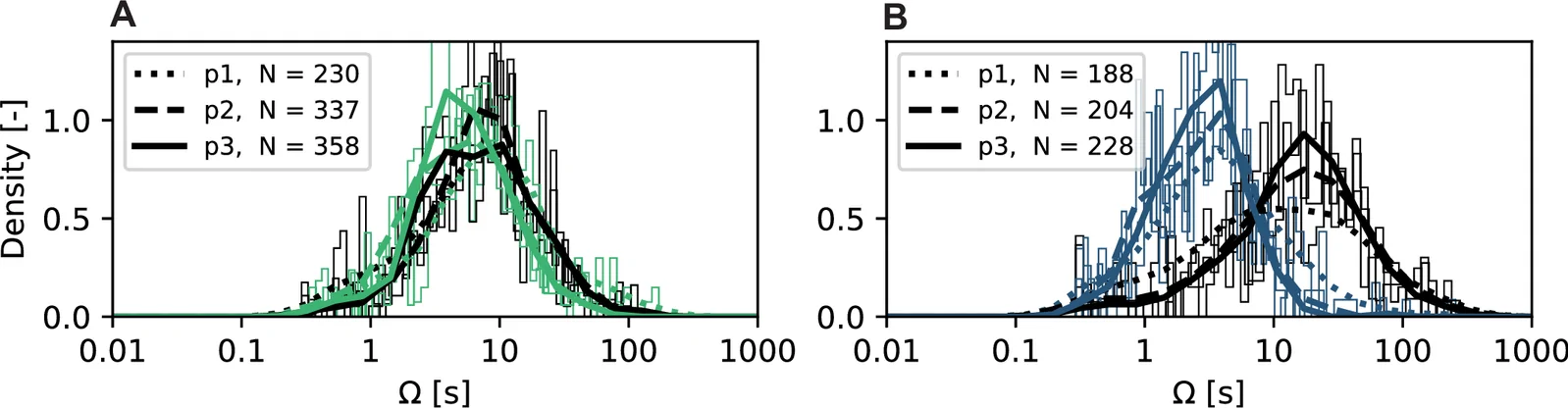
Underlying probability density distributions of the durations $\mathrm{\Omega }$ of warm- and cold-water excursions for TR1 (**A**) and TR3 (**B**), respectively. The distributions of the warm- and cold-water excursion durations become increasingly distinct over exposure time for TR3 but not TR1. Excursions at 12 °C (above the interface) are shown in black, whereas excursions in the lower, colder water are depicted in the respective color (green: $\mathrm{T}_{\mathrm{b}\mathrm{o}\mathrm{t}\mathrm{t}\mathrm{o}\mathrm{m}}={10\,}^{\circ} {\rm C}$; blue: $\mathrm{T}_{\mathrm{b}\mathrm{o}\mathrm{t}\mathrm{t}\mathrm{o}\mathrm{m}}={6\,}^{\circ} {\rm C}$). Treatment phases are indicated by line type (p1–p3). Values of $\mathrm{N}$ indicate the total number of excursions of each type within that phase for 20 fish (as the excursions alternate, $\mathrm{N}$ is equal for cold and warm excursions). Underlying distributions are shown as histograms.

To understand whether this exploratory behavior of the colder water changed over the 18 min duration of the experiments, we analyzed the distribution of the excursion duration Ω of warm- and cold-water excursions separately for each phase (p1, p2, and p3). For TR1 ($T_{\mathrm{bottom}}={10\,}^{\circ} {\rm C} $, $T_{\mathrm{top}}={12\,} ^{\circ} {\rm C}$), cold and warm water excursions showed almost identical distributions of their durations, and the distributions changed little over time across the three phases ($\overset{\sim }{\Omega }_{\mathrm{warm}}= 6.7\ \mathrm{s},\ 7.5\ \mathrm{s},\ 7.3\ \mathrm{s}$ and $\overset{\sim }{\Omega }_{\mathrm{cold}}=8.4\ \mathrm{s},\ 4.9\ \mathrm{s},\ 5.1\ \mathrm{s}$, for p1, p2, and p3 respectively; Figure 4D). The same conclusions apply to all warm treatments (TR6–TR9; Figure 4—figure supplement 1E-H). In stark contrast, in cold-treatment TR3, the distributions of the durations of cold-water excursions (into $T_{\mathrm{bottom}}= {6\,}^{\circ} {\rm C}$) and warm-water excursions (into $T_{\mathrm{top}}={12\,}^{\circ} {\rm C}$) are clearly distinct already in phase p1, and this difference increases in p2 and p3 ($\overset{\sim }{\Omega }_{\mathrm{warm}} =9.8\ \mathrm{s},\ 15.5\ \mathrm{s},\ 15.8\ \mathrm{s}$ and $\overset{\sim }{\Omega }_{\mathrm{cold}}=3.7\ \mathrm{s},\ 2.8\ \mathrm{s},\ 2.8\ \mathrm{s}$ for p1, p2, and p3, respectively; Figure 4E). In particular, short warm-water excursions ($0.1\ \mathrm{s}< \Omega < 10\ \mathrm{s}$) and long cold-water excursions ($10\ \mathrm{s} < \Omega < 100\ \mathrm{s}$) became less frequent over time. A similar pattern is observed in TR2 and TR4 (Figure 4—figure supplement 1B, D).

### Fish swimming speed increases during short-term exposure to cold water

**Figure 5.**
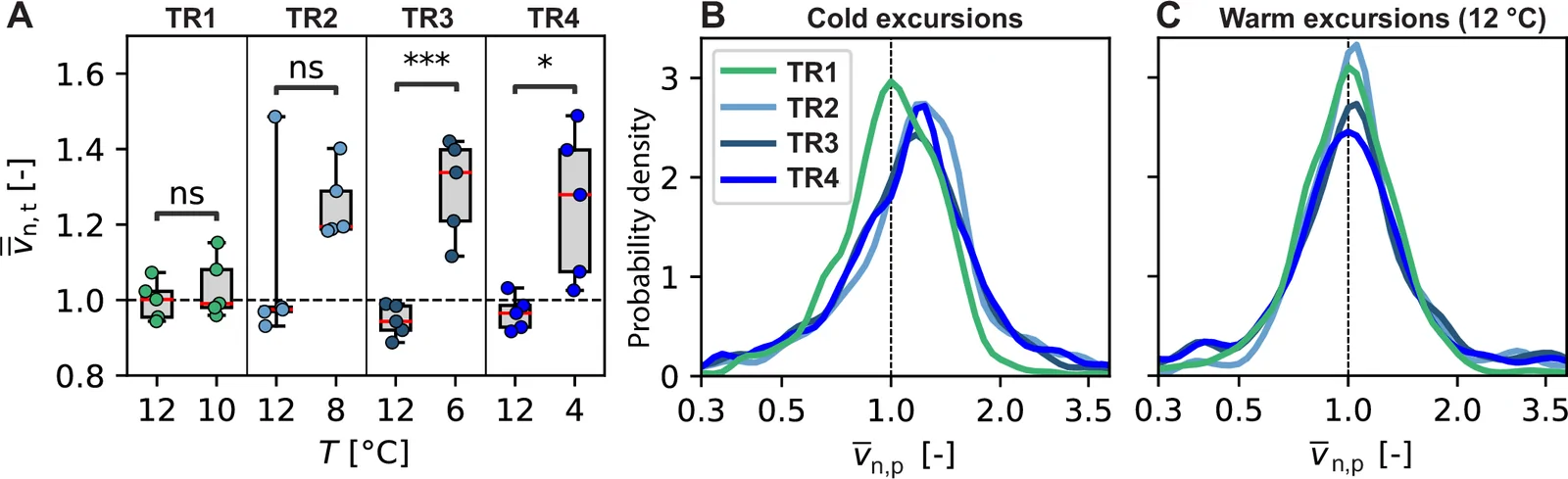
Fish swim more rapidly in the cold. (**A**) Mean swimming speed when above (in warmer water) and below (in colder water) the thermal interface. Speed was standardized by the average swimming speed of each individual during the treatment phases (p1–p3). Data were aggregated at the replicate level; hence ${\bar{\bar{\mathrm{v}}}}{\phantom{\mathrm{v}}}_{\mathrm{n},\mathrm{t}}$ represents the average response of the fish group (four fish) within one replicate. Symbols represent the results of pairwise t-tests (ns, $\mathrm{p}\mathrm{\, }> \mathrm{\, }0.05$; *, $0.01\mathrm{\ }< \mathrm{\, }\mathrm{p}\mathrm{\, }\leq \mathrm{\, }0.05$; ***, $0.0001\mathrm{\, }< \mathrm{\, }\mathrm{p}\mathrm{\, }\leq \mathrm{\, }0.001$, see also Appendix 4—table 3). Whiskers extend to the full range of observations and red lines indicate the median. The equivalent data for warm treatments are shown in Figure 5—figure supplement 1 and absolute swimming speeds are shown in Appendix 4—figure 2. (**B, C**) Probability density functions of averaged swimming speed during cold-water excursions (**B**) and warm-water excursions (**C**) in cold treatments (TR1–TR4). Speed was standardized by the average swimming speed of each individual within each phase and $\overset{-}{\mathrm{v}}_{\mathrm{n},\mathrm{p}}$ represents the average of this quantity over the duration of each individual excursion. The total numbers of cold-water excursions are TR1, $\mathrm{n}=889$; TR2, $\mathrm{n}=389$; TR3, $\mathrm{n}=611$; and TR4, $\mathrm{n}=452$; with identical numbers of warm-water excursions for each treatment. A kernel density estimator was fitted to the normalized histogram after $\log _{10}$-transforming the data. Equivalent Figures for warm treatments are shown in Figure 5—figure supplement 2. Excursion durations were scattered against average normed swimming speed (see Appendix 11—figure 1).

In addition to displaying a thermotactic behavior in modulating their turning events upon experiencing temperature gradients, fish further reduced cold-water exposure through a thermokinetic behavior, by increasing their swimming speed when in cold water. For $T_{\mathrm{bottom}} \leq {6\,}^{\circ} {\rm C}$ (TR3 and TR4), the swimming speed normalized by the mean speed of each fish during the entire treatment phase, $\bar{\bar {v}}_{n,t}$ (*Methods*), was significantly greater (44% in TR3; 33% in TR4) when fish were located below compared to above the thermal interface (*t*-test, p < 0.0001 and p < 0.05, respectively, Figure 5A*,* Appendix 4—tables 1–4). For TR2 ($T_{\mathrm{bottom}}={8\,}^{\circ} {\rm C}$), a similar increase (22%) was observed, but was not statistically significant due to outliers (likely cold-shocked fish) in one replicate (Figure 5A). In contrast, in TR1 the swimming speed was similar during cold-water ($T_{\mathrm{bottom}}={10\,}^{\circ} {\rm C}$) and warm-water ($T_{\mathrm{top}} = {12\,}^{\circ} {\rm C}$) excursions (Figure 5A).

**Figure 5—figure supplement 1.**
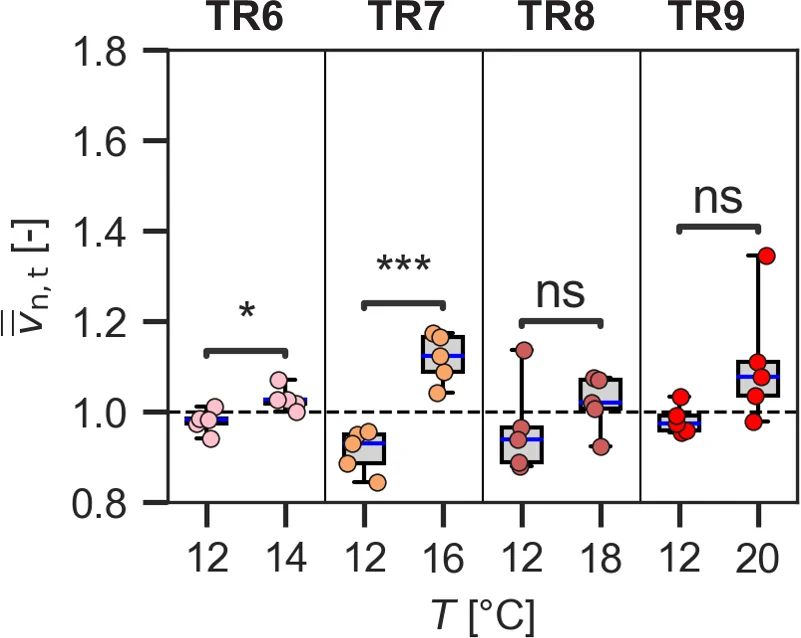
Mean swimming speed when above (in warmer water) and below (in colder water) the thermal interface. Speed was standardized by the average swimming speed of each individual during the treatment phases (p1–p3). Data were aggregated at the replicate level; hence ${\bar{\bar{\mathrm{v}}}}_{\mathrm{n},\mathrm{t}}$ represents the average response of the fish group (four fish) within one replicate. Symbols represent the results of t-tests (ns, $\mathrm{p}> 0.05$; *, $0.01< \mathrm{p}\leq 0.05$; ***, $0.0001< \mathrm{p}\leq 0.001$, see also Appendix 4—table 4). Whiskers extend to the full range of observations and blue lines indicate the median.

**Figure 5—figure supplement 2.**
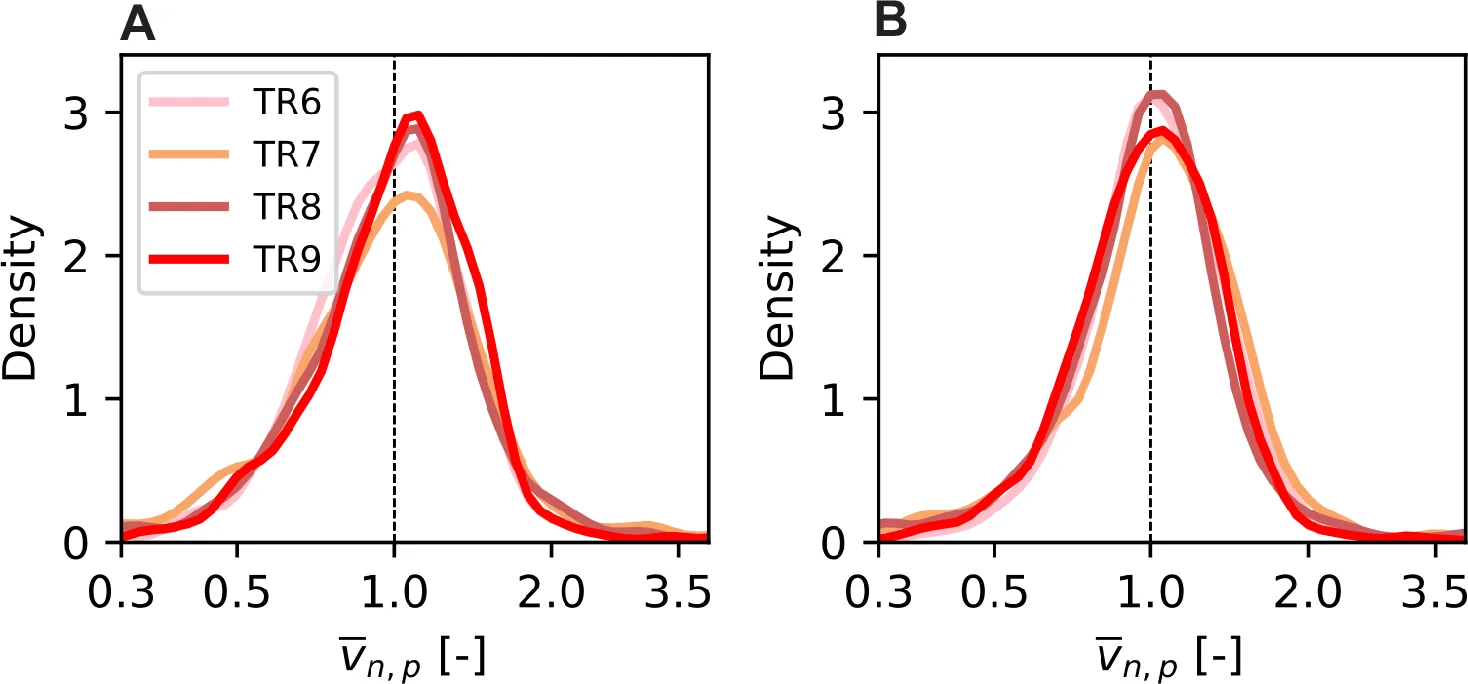
Averaged phase normalized swimming speed during warm water excursions (**A**) and cold-water excursions (**B**) in warm treatments (TR6, TR7, TR8, and TR9). Data has been transformed to $\mathrm{l}\mathrm{o}\mathrm{g}_{10}$ and a kernel density estimator was fitted to the normalized histogram.

Swimming speed increased over time (from p1 to p3) across all treatments (Appendix 4—figure 2). To account for this temporal trend in the analysis of cold-water excursions, we further normalized the swimming speed by the average speed of that fish during the corresponding phase, $v_{n,p}$. To give each excursion the same weight, independently of its duration, we further averaged that quantity for each excursion, $\bar{v}_{n,p}$. Notably, the distribution of average swimming speeds during cold-water excursions in cold treatments with $T_{\mathrm{bottom}}\ \leq \ {8\,}^{\circ} {\rm C}$ (TR2–TR4) is clearly skewed toward values larger than $\bar{v}_{n,p}= 1$. This indicates that the swimming speed was above average during the majority of cold-water excursions (Figure 5B), indicating the presence of a thermokinetic response. In contrast, for TR1 ($T_{\mathrm{bottom}}={10\,}^{\circ} {\rm C}$) the speed during cold-water excursions peaks at $\bar{v}_{n,p} = 1$ (Figure 5B), similar to the warm-water excursions across all cold-water treatments (Figure 5C), indicating the absence of a thermokinetic response in these cases. This observation was further supported by more frequently detected high absolute swimming speeds (>20 cm/s) during cold-water occupation in TR2–TR4 (Appendix 10—figure 1). These findings demonstrate that fish exhibit an increase in swimming speed during excursions in cold water ($T_{\mathrm{bottom}}\leq {8\,}^{\circ} {\rm C}$), indicative of a rapid and transient thermokinetic response, on a timescale of seconds, to cold-water exposure.

## Discussion

Using a novel experimental design, we showed that trout parr acclimated to 12 °C display within minutes a strong avoidance of incoming cold water ($T_{\mathrm{bottom}}={8,\ 6,\ 4\,}^{\circ} {\rm C}$) through a combination of thermotactic and thermokinetic behavior, whereas they do not avoid incoming warm water ($T_{\mathrm{top}}= 14,\, 16,\, 18,\, {20\,}^{\circ} {\text {C}}$). Other fish species have been shown to avoid unfavorable thermal conditions, although often over timescales of hours to days (Berman and Quinn, 1991; Magnuson et al., 1979; Sutton et al., 2007). For example, vertical relocation over timescales of hours has been observed for marine sablefish, *Anoplopoma fimbria*, acclimated to 12 °C and exposed to 2–8 °C (Sogard and Olla, 1998).

Our observations revealed that the avoidance of cold water was mediated by two distinct behavioral responses: fish biased their vertical turns, which enabled them to redirect their swimming out of the colder water, and increased their swimming speed, which allowed them to reduce the time spent in the colder water. Cold-water excursions were shorter in duration and shallower as the temperature of the cold water decreased. Larval zebrafish have been found to exhibit thermotactic behavior similar in some respects to that of trout in our experiments, biasing both turn magnitude and turn direction to swim toward warmer water (Gillooly et al., 2001). However, in contrast to our observation of increased swimming speeds in colder water, swimming activity of larval zebrafish decreased in cold water (Le Goc et al., 2021). Furthermore, to guide their movement, zebrafish have recently been found to integrate temperature information over a time window of 0.4 s preceding the next swimming bout (Haesemeyer et al., 2015). The duration of cold-water excursions of trout in our experiments was of a similar order of magnitude ($0.1\ \mathrm{s} < \Omega < 10\ \mathrm{s}$), suggesting that thermal sensing in trout may occur over similar timescales to zebrafish.

Surprisingly, no superficial cold receptor has yet been identified in trout skin (Ashley et al., 2007) or in fish more broadly (Haesemeyer, 2020), and recent studies suggest that fish may instead respond to temporal changes of their internal body temperature (Pépino et al., 2015). To investigate how body temperature might trigger behavioral responses, we used a heat transfer model for fish (Stevens and Sutterlin, 1976) to estimate the decrease in internal body temperature $T_{\mathrm{body}}$ over the duration of cold-water excursions (see Methods). We found that the median body temperature $T_{\mathrm{body}}$ during cold-water excursions decreased by only 0.2 °C in TR1 up to 0.5 °C in TR4 (Appendix 12—figure 1). This suggests that the avoidance response could have been triggered by a small decrease in internal body temperature, in the order of 0.2–0.5 °C. Further, this analysis indicates that by reducing the duration of cold-water excursions, fish maintained body temperatures within the range 11.5–12 °C, ultimately preventing body cooling and the need to acclimate physiologically to the colder temperature.

Our results suggest that the observed thermoregulation behavior in warm-water treatments is a strategy to gradually increase body temperature. When the temperature of the incoming water was higher than 12 °C (TR6–TR9), fish exhibited a bimodal distribution of depth occupancy that was distinctly different from the acclimation phase and the cold-water treatments. This bimodal distribution was not due to variability in the temperature preference among individual fish, but rather to individual fish periodically moving between warmer and colder regions (Figure 2—figure supplement 3B). Interestingly, fish gradually increased the occupancy of warmer water over time (Figure 2—figure supplement 1B). In contrast to the cold treatments, no avoidance response was observed at the thermal interface in warm treatments. We propose that this periodic relocation between warmer and colder regions is a behavioral strategy to slowly elevate body temperature while avoiding rapid and drastic body warming to 14–20 °C (TR6–TR9). It has indeed been shown theoretically that frequent transitions between cold- and warm-water regions that are each, per se, thermally suboptimal, could allow fish to maintain body temperatures between the available extremes while avoiding excessive heating or cooling (Pépino et al., 2015). Our observations resemble this mode of behavioral thermoregulation, in which fish progressively favor warmer regions within a heterothermal environment. However, additional experimental evidence is required to determine the mechanisms underlying this behavior.

We conclude from the behavior of fish when warmer water was available that their acute thermal preferendum exceeded 12 °C, departing from the acclimation temperature we had chosen based on the thermal preferendum for trout reported in literature (Reynolds and Casterlin, 1979b). Indeed, the thermal biology for any fish species is more complex than a single, static thermal preferendum. Many internal and external factors, such as hypoxia, satiation, time of day, and life stage (Haesemeyer, 2020), can influence the temperature preference of fish. For example, the level of satiation can have an impact because when fish are well fed, their growth rate increases with body temperature as metabolic performance increases (Gillooly et al., 2001). This modifies the preferred temperature, as observed in Bear Lake sculpin (*Cottus extensus*) that ascend into warmer water after feeding to stimulate digestion and thereby achieve a threefold higher growth rate (Wurtsbaugh and Neverman, 1988). In contrast, field studies with adult fish have observed movement from warm to cold water in summer (Chiaramonte et al., 2016; Goniea et al., 2006), allowing fish to lower their metabolic rate, likely in an effort to conserve energy (Brett, 1971; McLaren, 1963). We propose that the behavior of trout parr upon exposure to warmer water in our experiments served to achieve a higher body temperature to ultimately increase growth rate, which is critical for this life stage (Elliott and Hurley, 1995; Forseth et al., 2009). Indeed, growth experiments on brown trout populations have shown that optimal growth temperatures can range between 15 and 19 °C, depending on the stream of origin (Forseth et al., 2009).

The behavioral thermoregulation exhibited by trout in our experiments also included a strong thermokinesis response. Fish rapidly increased swimming speed while in colder water, on a timescale of seconds during individual cold-water excursions (in cold treatments TR2 -TR4). This contrasts with the established paradigm for thermokinesis in fish. For a variety of species including trout (Kieffer and Tufts, 1998), swimming speed was found to decrease with decreasing water temperature. This is thought to be a physiological effect, as a lower temperature results in a lower metabolic rate and locomotory capacity due to decreased contractile rates in the red and white swimming muscles and the heart (Vornanen, 1994). This type of thermokinetic response cannot serve to avoid cold water.

Our results instead suggest that trout parr employ a very different form of thermokinesis. Our analysis showed that increasing swimming speed during cold-water excursions allowed fish to shorten the exposure to cold water. This conclusion is supported by our model predictions that body temperature during cold-water excursions remained above 11.5 °C. Anecdotal observations from our experiments suggest that prolonged exposure to cold water (in the order of minutes) can nonetheless reduce swimming speed. Two individual fish in TR2 ($T_{\mathrm{bottom}} = {8\,}^{\circ} {\rm C}$) appear to have become trapped within the cold water (Figure 2—figure supplement 1A) and displayed reduced swimming speeds, in line with previously described deactivation upon cold-water exposure (Steiner and Olla, 1985; Taugbøl et al., 2019).

Taken together, these observations suggest that the thermokinetic response to cold water is timescale dependent. If the exposure to cold water is short (seconds), fish accelerate to escape the cold. If exposure is longer (minutes), however, fish slow down, likely due to a reduction in metabolic rate. Importantly, this timescale dependence implies that long-term (hours) measurements of behavior at constant water temperatures cannot be used to model fish movement response to thermal gradients occurring over shorter timescales. Our findings may help explain why recent modeling of fish behavior in heterothermal environments could not reproduce the observed avoidance of cold water in stationary thermal gradients (Le Goc et al., 2021).

Despite the avoidance response to cold water, fish engaged in repeated cold-water excursions, potentially reflecting a behavioral strategy to map the thermal environment. This pattern may also reflect an inherent tendency to occupy the lower part of the tank, as observed during homogeneous temperature of 12 °C during the acclimation phase. However, over time, the frequency of longer cold-excursions (>10 s) diminished, reflecting a modulation of this exploratory behavior. While most studies have associated thermoregulation with sustained relocation from colder to warmer water or vice versa (Amat-Trigo et al., 2023), it has been suggested that short excursions into water of a different temperature might be a thermoregulatory behavior that allows fish to exploit resources in suboptimal thermal regions within a heterothermal environment (Pépino et al., 2015). For example, by keeping excursions into cold water short, hammerhead sharks can access deep-sea foraging habitats without critically cooling their body temperature (Royer et al., 2023).

Our findings show that fish were able to perform upward turns while still located above the thermal interface and that is, before actually sampling the cold water below the interface. In fact, our simulation of the vertical extent of the thermal gradient revealed that a substantial fraction of upward turns occurred before fish encountered the gradient itself—that is, prior to any sensory detection of the temperature change (Appendix 9—figure 5). This finding may be evidence of associative learning, whereby fish used information on the presence of colder water at depth obtained at prior times. While the current data do not provide conclusive evidence in this regard, they prompt the possibility that, rather than responding solely to immediate thermal cues, fish use spatial memory or associative learning to anticipate the location of colder water based on prior experience. Indeed, fish are able to perform associative learning based on non-visual cues de A Moura and Luchiari, 2016; create mental maps of their surroundings Holbrook and Burt de Perera, 2011; and retain memory for hours (de P Cognato et al., 2012), days (Tarrant, 1966), and months (Triki and Bshary, 2019; Zion et al., 2012).

Determining the mechanisms underlying fish behavior in heterothermal environments is challenging because thermokinesis and thermotaxis may co-exist and play out across different spatio-temporal scales. In rivers and lakes, heterothermic environments form naturally and persist over a variety of spatial and temporal scales (Torgersen et al., 1999). Anthropogenic warm- and cold-water releases expose fish to rapid (minutes) thermal changes, known as thermopeaking (Antonetti et al., 2023), which can affect river sections hundreds of kilometers downstream (Toffolon et al., 2010). Here, we mimicked such rapid thermal changes for environmentally relevant thermal contrasts of 2–8 °C using a lock-release setup. We have demonstrated that trout use a combination of thermotaxis (modulation of turning behavior) and thermokinesis (modulation of swimming speed) to avoid cold water, while occasionally still exploring it through brief cold-water excursions. In contrast, we found that for incoming warm water, fish explore both warm- and cold-water regions and do not display any thermotactic avoidance.

While limited to laboratory conditions, these observations provide a framework for interpreting the thermal behavior of fish in nature. For example, a recent study observed a significant increase in downstream displacement for early life stages of a cypriniform fish in response to cold-water thermopeaking (Mameri et al., 2023). The authors hypothesized that this response was caused by reduced swimming capacity and cold shock. Our findings suggest that the observed downstream displacement could alternatively have been caused by an avoidance response at the interface with cold water. However, a generalization of our observations to horizontally oriented thermal gradients remains elusive. Our results are inherently tied to the vertical stratification created in our experiments. As warm water was always positioned above and cold water below, we could not control for the effect of vertical position (i.e. we could not do cold over warm layer experiments). This limits our ability to directly compare our findings to those obtained from horizontally oriented thermal gradients. On the other hand, the case we addressed is of direct environmental relevance, as natural waters often experience vertical thermal stratification.

By capturing the multifaceted response of fish to thermal change, our results may help to predict the larger-scale distribution of species and their response to unnatural disturbances, and thus inform environmental conservation policies. Our study also highlights the importance of deepening our understanding of the fundamental behavioral mechanisms of fish to alterations in their environment, particularly anthropogenic ones. Ultimately, the large-scale distribution of an organism results from the combined effects of responses to countless stimuli on the individual scale. Our experimental setup provides a platform to investigate these stimuli through a reductionist approach, and a blueprint for how to investigate responses to other stimuli such as chemicals or salinity for diverse aquatic organisms.

## Methods

### Experimental animals

A total of 180 hatchery-sourced trout (standard length 3.6±0.5 cm) were used for experimentation, and each individual was used in a single experiment only. Fish were acclimatized to the housing facility ($T_{\mathrm{water}} ={12\,}^{\circ} {\rm C}$) for a minimum of 24 hr prior to experimentation and kept for a maximum of 5 days.

Fish originated from wild mature trout, artificially spawned in a cantonal hatchery (Flüelen, canton Uri, Switzerland) in December 2020. Fertilized eggs were transported (35 min by car) in 200 mL plastic containers to the Swiss Federal Institute of Aquatic Science and Technology (Eawag, Kastanienbaum), where they were transferred to temperature-controlled incubators. The water temperature during transport and incubation was maintained at 3 ± 0.5 °C. The eggs were incubated for 3 months until hatching. After hatching, the water temperature in the incubators was incrementally increased to 5 ± 0.5 °C to promote growth of the alevins. After yolk-sac absorption, trout were fed with half a teaspoon of *Artemia* and chironomid larvae per 50 fish once per day for 1 month and then placed in a covered outdoor housing tank, with water temperature ranging from 8 to 11 °C. All incubators and housing tanks were fed by lake water (Lake Lucerne) in a flow-through system.

When fish had developed to the parr stage (Armstrong and Nislow, 2006), they were transported (65 min by car) in an aerated, temperature-controlled (11–12 °C) tank (60 L) to ETH Zurich (ETHZ), where they were acclimatized to the housing temperature of 12 ± 0.3 °C. Flow-through tap water continuously replenished the housing tank, water quality parameters (pH = 8–9, NH_4_ <0.04 ppm, NO_3_ <1 ppm, NO_2_ <0.04 ppm) were monitored twice per day, and the fish were fed in the evenings with frozen bloodworms (1.5% of body weight). Experimental procedures and husbandry facility were approved by the Zurich veterinary office (license no. 30997; housing facility no. 182). All fish were euthanized post-experimentation.

### Experimental setup

Experiments were conducted during daytime (09:00–17:00) in July 2021 at ETH Zurich. We performed experiments using an acrylic tank (dimensions 200 cm × 20 cm×30 cm, length ×width × height; Figure 1A), with a removable opaque vertical gate (polymethyl methacrylate, PMMA, white 60% transparency) separating the tank into two compartments of equal dimensions (Figure 1A, Figure 1—figure supplement 1). Three temperature probes (resistance thermoelement, type K-3895, Adafruit) were installed at three depths (4.5 cm, 14 cm, and 23.5 cm) in each compartment of the tank. A dissolved oxygen (DO) probe (WTW CellOx 325, galvanic, membrane based) was placed on each side wall of the tank (one at a depth of 4.5 cm and one at 23.5 cm, Figure 1A). Probes were synchronized and provided continuous measurements at a sampling rate of 0.2 Hz (Appendix 1—figures 2 and 3). At the beginning of an experiment, each compartment of the tank was filled with water to a depth of 28 cm (56 L per side). The initial water temperature on the left side was always 12 °C; water temperature on the right side varied according to treatment. Water on the right side was dyed blue by the addition of 16.66 mg pre-dissolved Patent Blue V sodium salt (C_27_H_31_N_2_NaO_7_S_2_, Sigma-Aldrich; final concentration in half tank 0.29 mg/L) to provide a visual contrast and allow visualization of the thermal interface during the experiment (Appendix 1—figure 1). Prior to the experiment, the temperature on each side was adjusted by adding ice or stirring with a heater until it reached the desired temperature homogeneously across depth. Throughout the experiments, the DO concentration remained within the range 7–10 mg/L. The tank was sheathed with white diffusive material (PMMA, white 60% transparency) and homogeneously illuminated with white LED light (4000 Kelvin) from all sides except the top and front of the tank. This homogeneous illumination and tank sheathing was necessary to provide sufficient contrast for image-based tracking of fish.

### Experimental procedure

Each experiment consisted of four fish exposed to a temperature treatment. In total, we conducted five experimental replicates per treatment. Treatments were defined based on the initial water temperature in the right compartment of the tank (values of 4–20 °C, in 2 °C increments; Appendix 1—table 1). Therefore, for all treatments, fish were initially located and acclimatized to 12 °C (the *thermal preferendum* of adult trout reported in the literature Reynolds and Casterlin, 1979b) and exposed to a thermal interface toward warmer water on the top or colder water on the bottom. All water temperatures were within the thermal tolerance range of the species (0–25 °C; Elliott and Elliott, 2010) and specifically within the thermal range in which trout show positive somatic growth rates (3.6–19.5 °C in Elliott and Hurley, 1995; approximately 5–23 °C in Forseth et al., 2009). After 15 min acclimation to 12 °C (Figure 1A), the central gate was removed, triggering gravity currents (Wood, 1970) caused by the density difference resulting from the water temperature difference between the left and right compartments of the tank (Appendix 1—figure 1). During the treatment phase, a sharp thermal gradient (i.e. a thermal interface) spanned the entire width of the tank and gradually stabilized at the half-tank depth such that its temporal vertical movement reduced to ~5 cm (Figure 1—figure supplement 4). The interface could be visualized throughout experiments due to the blue dye in the water that was initially well mixed into the right compartment of the tank (see Figure 1A and B*,* Appendix 13—Videos 1–4, and Figure 1—figure supplement 2).

Each experiment consisted of a 15-min acclimation period followed by a 20-min treatment period (Figure 1D). A group of four fish was released into the left compartment of the tank containing water at 12 °C (identical to the husbandry temperature). After 3 min, cameras were started, initiating the acclimation phase. After 15 min, the lock was gently removed by hand (~3 s), exposing fish to the gravity current. For the next 20 min, fish were exposed to the heterothermal environment throughout the tank. After each experiment, fish were captured and transferred to a separate compartment of the husbandry facility. To capture temporal trends in the behavioral response, we compared four 6-min periods (acclimation, p1, p2, and p3, Figure 1D). During the treatment period, fish could choose to occupy lower, colder water or upper, warmer water. The final 6 min of the acclimation phase provided a reference for the absence of thermal contrasts. Phase 1 (p1) started 2 min after the lock was removed and the thermal interface had already occupied the entire width of the tank (Figure 1D). Phase 2 and phase 3 (p2 and p3) were the consecutive 6 min periods, during which the vertical movement of the thermal interface line diminished (Appendix 1—figure 1).

### Gravity currents

A gravity current is the flow of one fluid within another caused by the density difference between the fluids (Simpson, 1982). In our setup, this density difference was caused by the different water temperatures which defined our treatments (Appendix 1—table 1). The treatment phase of the experiment was initiated with the slow removal (~3 s) of the vertical lock separating the two compartments of the tank, followed by initial gravity currents (Shin et al., 2004) and the formation of a horizontal thermal interface (Appendix 1—figure 1). The symmetric warm and cold water currents traveled at similar speeds but in opposite directions (Shin et al., 2004; Appendix 13—Videos 1 and 3). Water from the colder compartment traveled along the tank bottom and remained sharply separated from the warm water above it. For similar thermal contrasts, it has been shown that despite some mixing, the interface between the two counter-flowing layers is stable if the density ratio $\rho _{\mathrm{left}}/\rho _{\mathrm{right}}$ is close to unity (Shin et al., 2004), which was the case for all our treatments (Appendix 1—table 1). Once the interface was established, the temperature contrast would smear out due to thermal diffusion. We simulated numerically (in Python) the thermal diffusion for the strongest thermal contrast (d*T* = 8 °C) and found that over the duration of an experiment (20 min), an initially sharp thermal interface (gradient thickness = 0) evolves into a gradient of thickness of ~4–6 cm (Appendix 7—figure 2C). The density ratio between colder and warmer fluid was close to unity, resulting in slowly creeping currents with maximum velocities of ~0.8–1.6 cm/s during the initial 2 min after gate removal. To eliminate the effects of flow on fish behavior, this period was excluded from the data analysis. After 1–2 min, the currents impounded at the side walls and the thermal interface extended over the entire width of the tank. Vertical movement of the interface (~0.1 cm/s) was much slower than fish swimming speeds (4–6 cm/s; Appendix 4—figure 2, Appendix 13—Videos 1–4) and over time, the thermal interface stabilized at the half depth of the tank (Figure 1—figure supplement 4).

### Imaging and tracking of fish

To allow simultaneous tracking of fish behavior and the position of the thermal interface, experiments were continually filmed using a video camera (GoPro hero7) mounted at a distance of 1.8 m from the front of the tank, recording color images at a resolution of 9.2 pixel/cm. The camera setting ‘linear field of view’ was selected to eliminate fish-eye effect typically captured by the camera's wide-angle lens, without compromising image quality.

We analyzed 26.3 hr of video recordings (45 experimental replicates) and obtained individual movement trajectories at a temporal resolution of 24 fps (Appendix 3—figures 1 and 2) and an average tracking efficiency of 99% across all analyzed videos (Appendix 2—figure 1; Appendix 13—Videos 1 and 3). Each video was cropped using custom-made Python scripts, and the start of the treatment phase was set at the time when the vertical gate detached from the water surface. As the blue dye was not visible in the red channel of the RGB image (Figure 1—figure supplement 3C), fish tracking was performed on the red channel using the open-source software TRex (Walter and Couzin, 2021). Light conditions and dye concentration were identical for all experiments, allowing the use of an optimized set of tracking parameters (Appendix 2—table 1). Due to the low density of fish in the tank, occlusions (in which two fish temporarily merge into one object) rarely occurred; however, the automated tracking procedure was reviewed for all videos. In difficult cases (e.g. when one individual was occluded by another moving in the same direction), individual identities were manually assigned based on simultaneous recording from a camera positioned above the tank.

### Visualization and evolution of the thermal interface

The dye used to visualize the thermal interface, Patent Blue V, is a non-toxic dye (Johnston et al., 1999) that has previously been injected without side effects into the dorsal fin of experimental fish at concentrations of up to 300 mg/mL (Bayani et al., 2017). However, to confirm the absence of potential side effects of the dye on fish behavior, five control experiments to TR3 ($T_{\mathrm{bottom}}= {6\,}^{\circ}{\text {C}}$) were performed without the addition of the dye. In the comparison of results from these control experiments with TR3, no statistically significant difference in vertical occupancy was detected (Appendix 6—figures 1 and 2). We therefore conclude that the presence of the dye did not affect fish swimming behavior. Furthermore, because the dye concentration and resulting color intensity were consistent across all treatments, the dye did not act as a confounding variable in our statistical comparisons.

Locating the thermal interface was central to relating fish movements to their immediate temperature environment. For each time instance (frame), a line, representing the thermal interface, was derived from the visual signal of the dye. To do this, an in-house image analysis pipeline was developed in Python OpenCV (Bradski, 2000). The pipeline consisted of background subtraction, thresholding, dilation, erosion, and finally the abstraction of the interface line $y_{I}\left (x,t\right)$ (python functions: cv2.threshold, cv2.dilate, cv2.erode, cv2.Laplacian; Figure 1—figure supplement 3, Appendix 7—figure 1). Our analysis assumes that over the duration of p1+p2+p3=18 min, the abstracted color boundary is representative of the location of the thermal contrast. We thus assume that the molecular diffusion driving heat exchange and molecular diffusion of the dye acted on similar time-scales.

### Characterization of the thermal gradient

Due to the combined effects of turbulence and thermal diffusion, the thermal gradient gradually weakens and expands over time. To quantify its vertical extent, we simulate the evolution of the gravity current over a 20-min period following its release. To account for transient mixing during the initial movement of the gravity current (within the first 2 min after removing the gate), we adopt a phenomenological approach to estimate the gradient thickness after the gravity current first hits the side wall of the tank. Then we predict the effect of thermal diffusion in a numerical simulation. This approach accounts for transient mixing in the initial, more turbulent phase, and provides a realistic estimation of the subsequent evolution of the thermal gradient by diffusion.

First, we introduce two types of interface thickness: the enstrophy thickness ($\delta _{\upsilon }$) and the temperature thickness ($\delta _{\kappa }$). From the thermal diffusivity ($\kappa = 1.4\ \cdot 10^{-7}\ m^{2}/s$) and the momentum diffusivity (i.e. kinematic viscosity) of water ($\nu = 10^{-6}\ \ m^{2}/s$), we obtain the Prandtl number, $Pr=\nu /\kappa =7.14$. In turbulence, the enstrophy thickness can be approximated (Holzner and Lüthi, 2011) by $\delta _{\upsilon } =10\eta $, where $\eta =\left (\frac{\nu ^{3}}{\epsilon }\right)^{1/4}$ is the Kolmogorov length scale and ϵ is the turbulent energy dissipation rate. Thus, the thickness of the thermal interface is given by $\delta _{\kappa }\backsimeq \delta _{\upsilon }/\sqrt{Pr}$ (Batchelor, 1959), which in our case amounts to $\delta _{\kappa }=3.7\eta $.

The Kolmogorov length can be estimated from large-scale balances as follows. The large-scale velocity can be estimated as $u_{0}= \sqrt{h_{0}g\Delta \rho /\rho }$, where g is the gravitational acceleration, ρ is the mean water density, $\Delta \rho $ is the density difference, and $h_{0}$ corresponds to half of the total water depth (Shin et al., 2004). Note that this scaling assumes that the large-scale fluid motion is independent of viscosity. To verify this assumption, we measured the propagation speed of the gravity current $u_{p}$ in our experimental videos for each cold-water treatment (TR1–TR4) and plotted it against the theoretical propagation velocity $u_{0}$ (Appendix 7—figure 2A). The good fit between the theoretical and observed values ($R^{2}= 0.96$) confirms that the flow is indeed large-scale dominated. Therefore, the Kolmogorov length η can be estimated by using the production-dissipation balance, $\epsilon =Cu_{p}^{3}/h_{0}$, for each temperature contrast in the cold-water treatments (TR1–TR4), where C is a non-dimensional constant of order unity that can vary among different flow conditions.

Assuming C=1, we calculated the turbulent energy dissipation rate and, subsequently, the Kolmogorov length for each cold-water treatment (Appendix 7—figure 2B). Across our treatments, we found a maximum estimated Kolmogorov length of $\eta \approx \, 0.76\, \mathrm{mm}$ in TR1 (Appendix 7—figure 2B), indicating that turbulence caused the thermal interface thickness to reach $\delta _{\kappa }=3.7\, \eta =2.8\, \mathrm{mm}$. To account for turbulence, we initialized thermal diffusion simulations by using this value of $\delta _{\kappa }$ as the initial interface thickness, and we ran a numerical simulation of thermal diffusion for $t=20\ \min $. After 20 min, the interface thickness reached a maximum of ∼4 cm in TR1 and ∼6 cm in TR4, depending on the thermal contrast ∆T (Appendix 7—figure 2C).

### Identification of time spent in colder and warmer regions

The interface line was used to differentiate whether fish were located in the warmer water above or the colder water below. To do this, we derived the vertical distance from the thermal interface $D\left (t\right)$

(1) $$\displaystyle D\left (t\right) = y_{I}\left (x\left (t\right),\ t\right)- y\left (t\right),$$

where $y\left (t\right)$ is the vertical position of an individual fish and $y_{I}\left (x\left (t\right),t\right)$ is the vertical position of the interface line at the lateral position of the fish $x\left (t\right)$ (Appendix 8—figure 1). Evaluating the sign of $D\left (t\right)$ reveals whether a fish was located above or below the thermal interface (Figure 2A and B).

### Cold- and warm-water excursions

Evaluating the sign of $D\left (t\right)$ allowed the individual fish trajectories to be partitioned into periods of cold- and warm-water occupation (i.e. excursions; Figure 2B). We counted the number of cold- and warm-water excursions ($N_{\mathrm{warm}}$ and $N_{\mathrm{cold}}$), which represents the number of times each fish crossed the thermal interface in one direction ($N_{\mathrm{crossings}}$), and for each excursion, we quantified the duration Ω, and the maximum distance to the interface line ($D_{\max }$). Excursions can be interpreted as periods of self-inflicted exposure to the cold or warm water.

We derived the duration Ω of individual cold and warm water excursions as

(2) $$\displaystyle \Omega _{\mathrm{warm}} = \int \ \left (D_{\mathrm{fish}}\left (t\right) > 0\right)\ dt$$

(3) $$\displaystyle \Omega _{\mathrm{cold}}=\int \left (D_{\mathrm{fish}}\left (t\right)< 0\right)dt,$$

and the maximum excursion depth during cold excursions as

(4) $$\displaystyle D_{\max } =\max |\left (D_{\mathrm{fish}}\left (t\right)< 0\right)|$$

### Time spent in cold and warm regions

We calculated the total time spent above and below the interface as

(5) $$\displaystyle  t_{\mathrm{warm}} = \sum \Omega _{\mathrm{warm}}$$

(6) $$\displaystyle  t_{\mathrm{cold}}=\sum \Omega _{\mathrm{cold}}$$

For cold treatments, the time $t_{\mathrm{warm}}$ represents the time an individual spent above the thermal interface (at 12 °C, which is equal to the acclimation and housing temperature), and $t_{\mathrm{cold}}$ the time spent in the respective treatment temperature (4, 6, 8, or 10 °C). In the warm treatments, the time $t_{\mathrm{cold}}$ represents the time the individual spent at 12 °C, and $t_{\mathrm{warm}}$ the time spent in the respective treatment temperature (14, 16, 18, or 20 °C).

### Body cooling during cold-water excursions

To estimate fish body cooling during cold-water excursions in TR1–TR4, we applied a heat transfer model and calculated the fish’s body temperature after each cold-water excursion (Pépino et al., 2015). Firstly, the heat transfer rate k for a fish with mass $m_{\mathrm{body}} = 4\ \mathrm{g}$ was estimated based on the parameters a=2.267 and b=−0.329 (Appendix 12—table 1):

(7) $$\displaystyle k = b* m_{\mathrm{body}}^{b} = 1.37\ min ^{-1}$$

We then assumed an initial body temperature of $T_{b} = 12 ^\circ \text {C}$ (corresponding to the fish residing above gradient for a prolonged period of time) and simulated body cooling during measured durations Ω of excursions into colder water with temperature $T_{a}$. Thereby, the rate of change of fish body temperature is given by:

(8) $$\displaystyle \frac{dT_{b}}{dt}=k*\left (T_{a}-T_{b}\right).$$

Thus, assuming the cold-water temperature to be a single, constant value (i.e. neglecting the effect of the thermal gradient), the fish body temperature after a cold-water excursion with duration Ω is:

(9) $$\displaystyle T_{b}\left (t=\Omega \right)=\left (T_{b}\left (t=0\right)-T_{a}\right)\ e^{-k\Omega }+T_{a}.$$

Based on the measured excursion durations in TR1–TR4 (Figure 4D and E and Appendix 8—figure 2A-D), Equation 9 allowed us to estimate the median body temperature ($T_{b}$) change during cold-water excursions (Appendix 12—figure 1).

### Vertical turning points

Vertical turning points were obtained by fitting a fifth-order polynomial to the vertical position signal $Y\left (t\right)$ of each trajectory within a moving window of 2.1 s (50 frames; using the Python script scipy.signal.savgol_filter, Press and Teukolsky, 1990). This procedure smoothed the vertical position, reducing the noise produced by small-scale variation. We then detected upward turns as local minima and downward turns as local maxima in this smoothed signal (Appendix 9—figure 1). To focus on turning points that resulted in considerable vertical displacements, we eliminated upward- or downward-turn events if a subsequent turn occurred within a vertical distance of d*y* ≤3 cm (Appendix 9—figure 2).

### Swimming speed

The normalized swimming speed for each individual was derived by dividing the instantaneous swimming speed by the average swimming speed of the individual during (i) the entire treatment period of 18 min ($v_{n,t}$) or (ii) each phase separately ($v_{n,p}$), as described within individual figure captions.

Upon visual inspection, the individual swimming trajectories seemed reasonably smooth (Appendix 4—figure 1A); however, unrealistic fluctuations appear in their first-order derivatives (i.e. fish swimming speed; Appendix 4—figure 1C and D). Before calculating swimming speeds, we therefore fitted a third-order polynomial to the trajectories obtained from ‘TRex’ (Walter and Couzin, 2021), using a fitted to the x,y coordinates. For each time step, the polynomial was fitted to a temporal window of 21 measurements (1.4 s) following a procedure described by Lüthi, 2002 (Appendix 4—figure 1B-E, Appendix 13—Video 5). Further, data gaps (arising mainly from fish occlusions) were filled using temporal linear interpolation, and an upper velocity threshold of 100 cm/s (i.e. an unrealistically high speed of approximately 3× burst swimming) was used to discard unrealistically high values.

### Statistical analysis

The number of replicates used in this study reflects a balance between statistical rigor and the ethical imperative to minimize the use of animals in experimentation. Regarding statistical power, our design (five replicates with groups of four fish each) is consistent with similar studies and represents an adequate sample size.

To compare behavioral responses between treatments, all analyses considered the mean or median values for each group of four individuals within each replicate, accounting for dependency between individuals. Therefore, to compare the number (Figure 3C) of cold-water excursions across cold-water treatments, one-way ANOVA was used (Appendix 8—tables 4 and 5). Post-hoc pairwise *t*-tests were used to assess differences between treatments (Appendix 4—tables 3 and 4, Appendix 8—table 6).

To determine whether the duration (Figure 4B) and depth (Figure 4C) of cold-water excursions followed a trend across treatments (i.e. decreased or increased with decreasing treatment temperature), we used the multivariate Mann–Kendall test (Libiseller and Grimvall, 2002; Appendix 9—tables 1 and 2). We used pairwise *t*-tests to test whether the normalized swimming speeds differed during warm and cold-water occupation (Figure 5A). Prior to performing statistical analyses, data distribution (normality) was assessed using the Shapiro-Wilk test (Appendix 4—tables 1 and 2, Appendix 8—table 4). All statistical tests were conducted using Python packages.

## Figures and Tables

**Appendix 1—figure 1.**
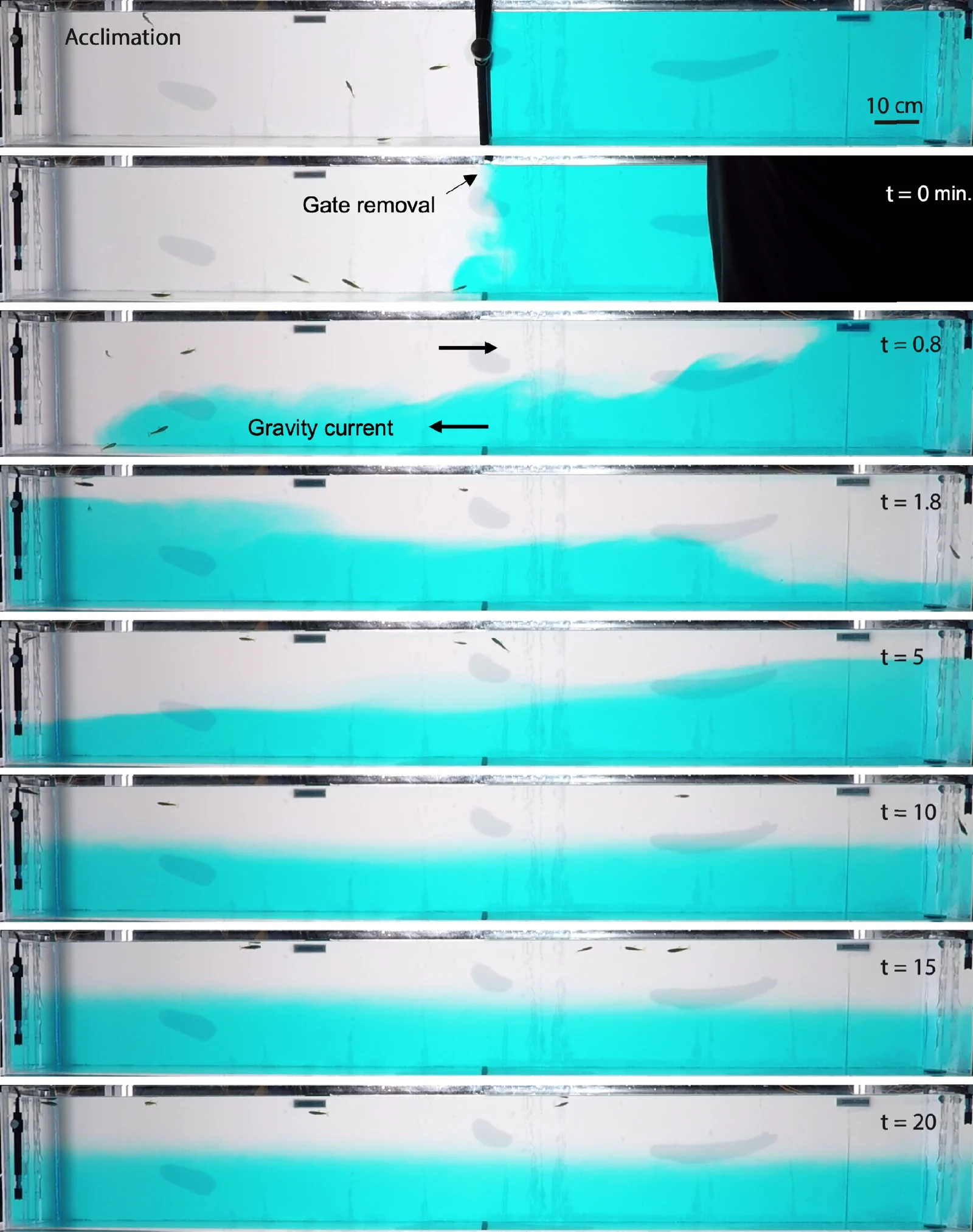
Side view of temporal progression of gravity current for cold treatment 4 (TR4). A stationary vertical stratification (i.e. the water is practically still) is reached only after about *t* = 10 min.

**Appendix 1—figure 2.**
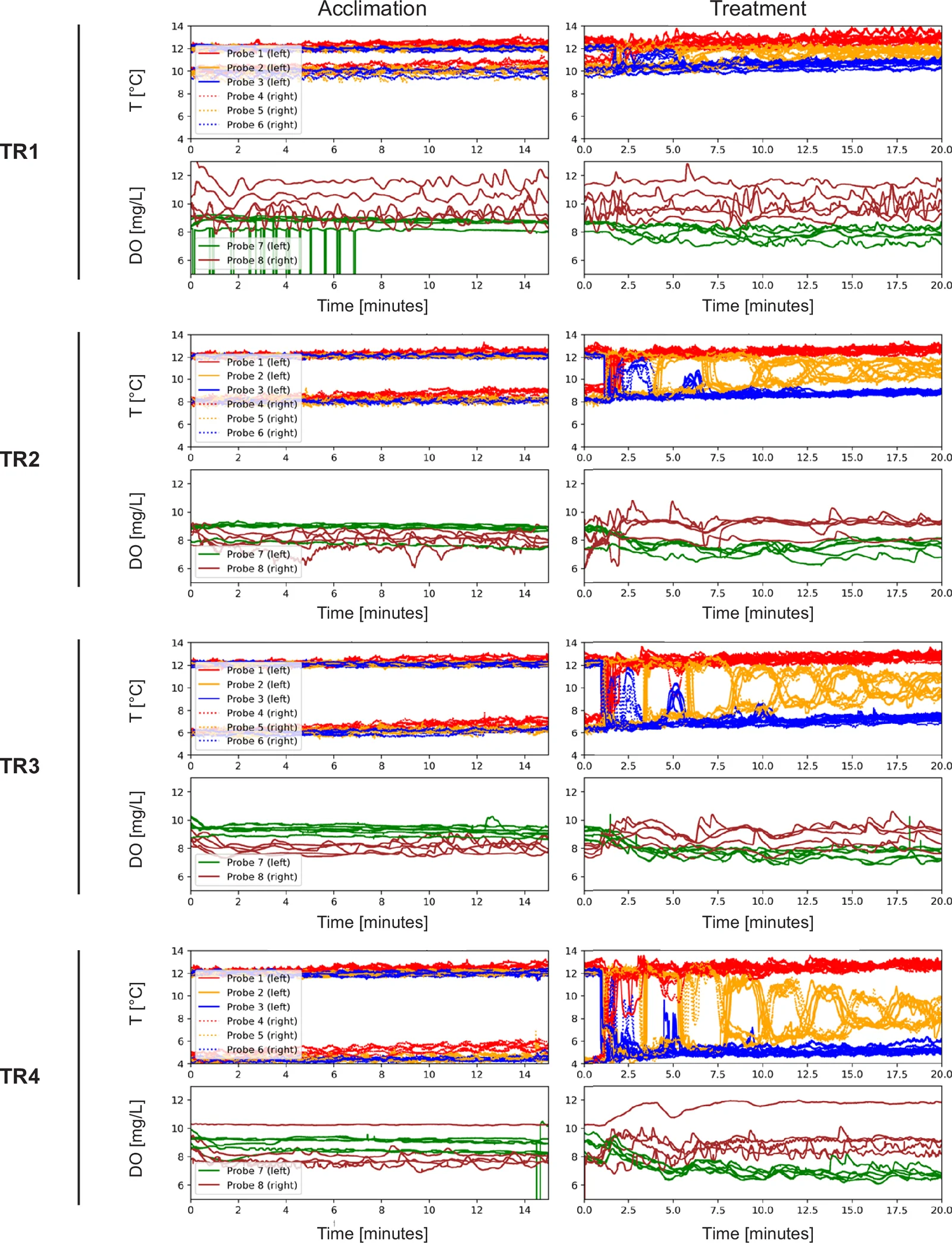
Cold treatments. Time traces of temperature [°C] and dissolved oxygen concentration [mg/L] in experiments during acclimation (left) and treatment phase (right). Temperature (Probe 1-6) was measured at three different depths (red, orange, blue) at both side walls of the experimental tank for all experiments (upper plots). Dissolved oxygen was measured on both sides of the tank. All measurements were continuously obtained at a frequency of 5 Hz.

**Appendix 1—figure 3.**
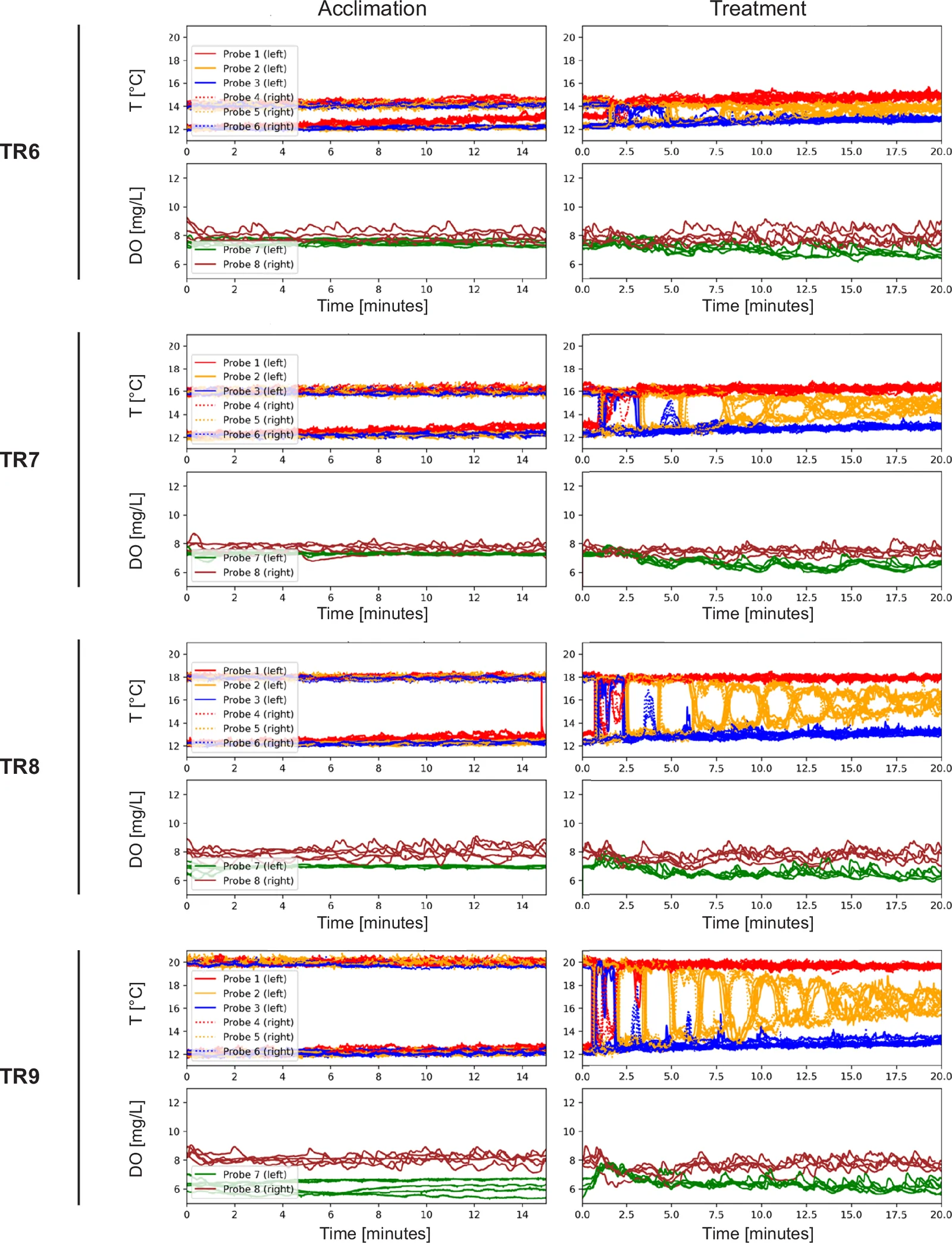
Warm treatments. Time traces of temperature [°C] and dissolved oxygen concentration [mg/L] in experiments during acclimation (left) and treatment phase (right). Temperature (Probe 1-6) was measured at three different depths (red, orange, blue) at both side walls of the experimental tank for all experiments (upper plots). Dissolved oxygen was measured on both sides of the tank. All measurements were continuously obtained at a frequency of 5 Hz.

**Appendix 2—figure 1.**
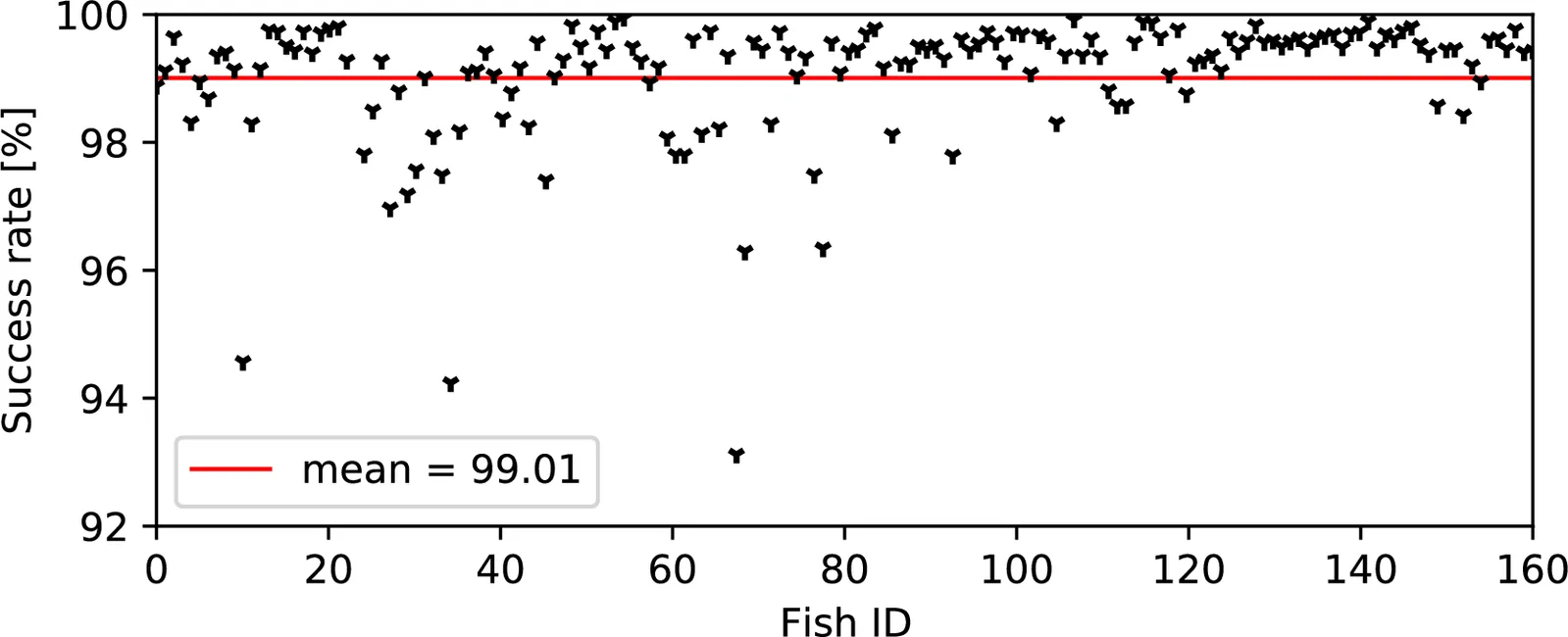
Tracking success rate for each individual fish during experimental phases (p1, p2, and p3). Low values are explained by fish occluding over longer periods of time (without moving) or being located behind the dissolved oxygen probes. For these cases, a second camera perspective was used to resolve the occlusions, and data gaps were closed using linear interpolation. The open-source tracking software ‘TRex’ (67) was used to track fish at a temporal resolution of 24 frames/s.

**Appendix 3—figure 1.**
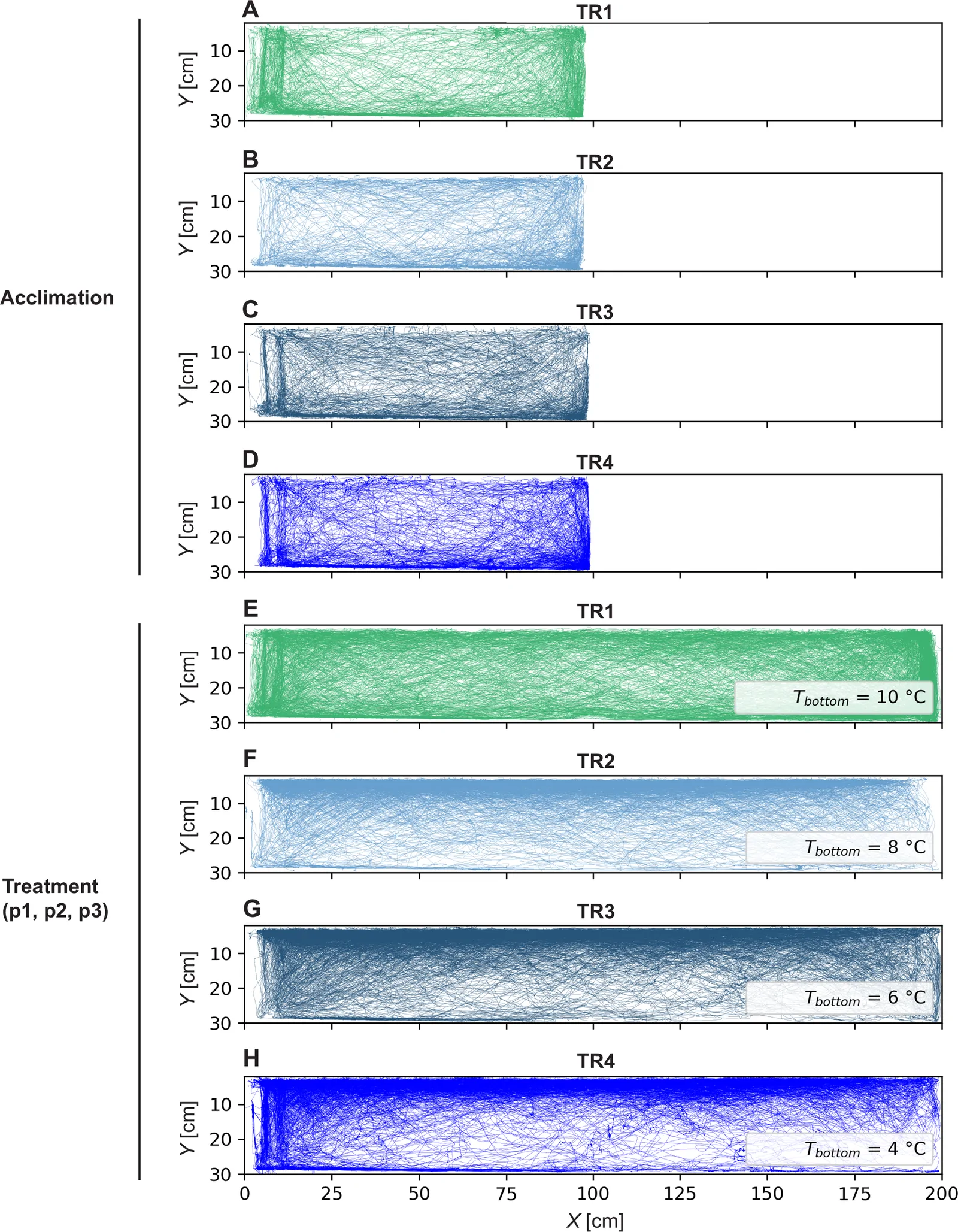
Fish tracking for cold treatments. Trajectories for all fish during acclimation phase (**A–D**) and treatment phases (**E–F**).

**Appendix 3—figure 2.**
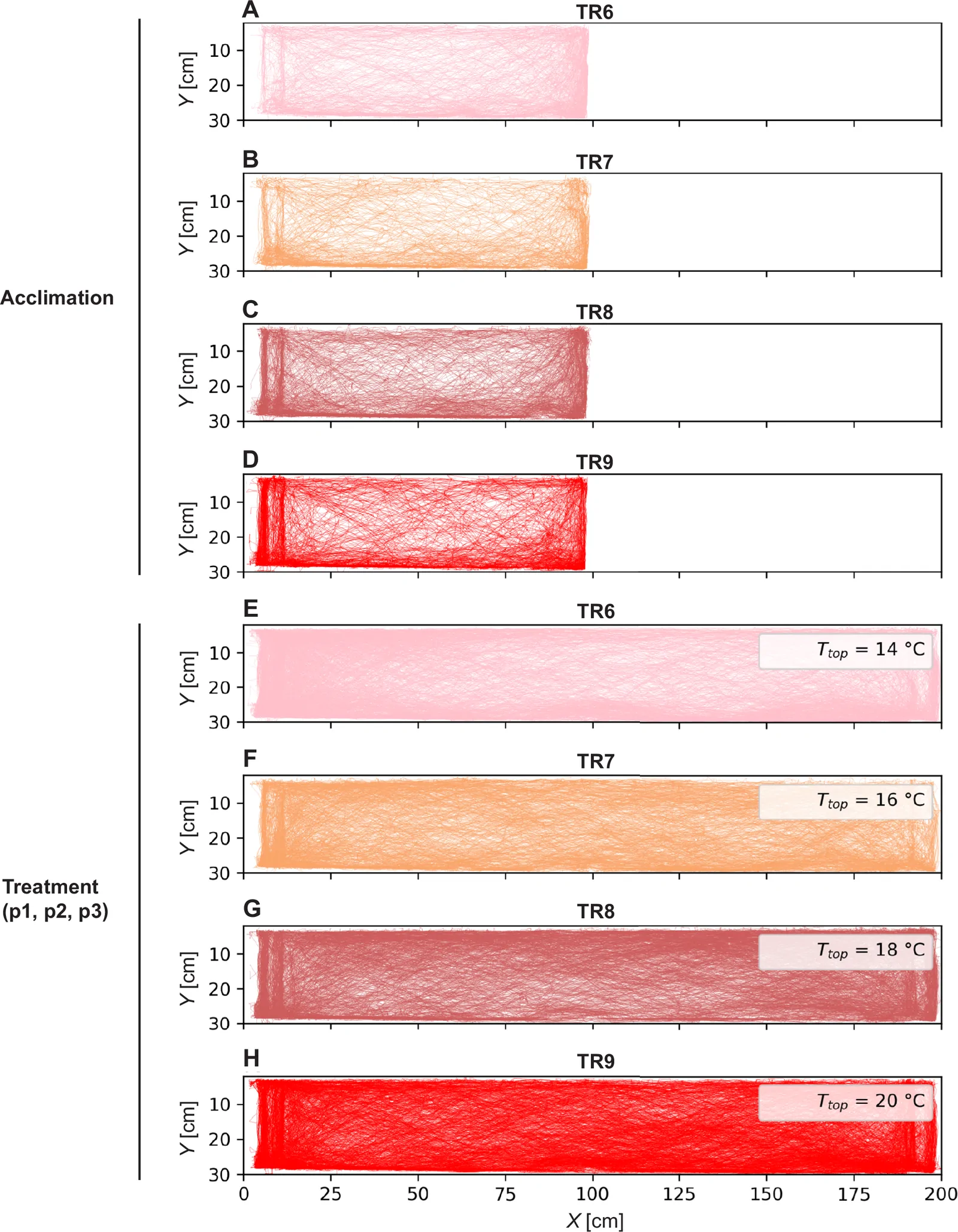
Fish tracking for warm treatments. Trajectories for all fish during acclimation phase (**A–D**) and treatment phases (**E–F**).

**Appendix 4—figure 1.**
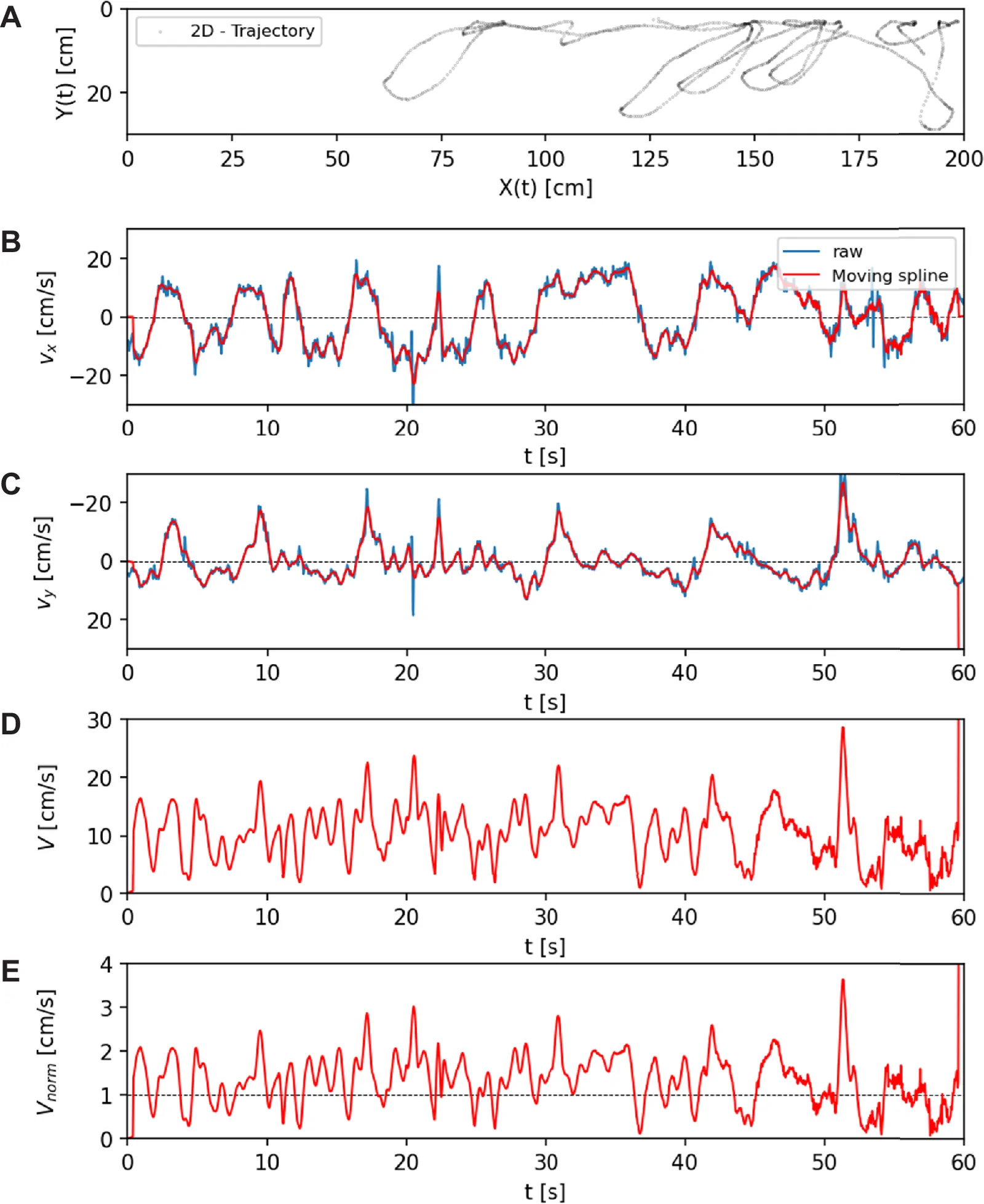
Example time traces illustrating the derivation of fish swimming speed (*V*) and normed swimming speed *V*_norm_ from the raw tracking output. (**A**) Raw trajectory output from tracking software (TRex) for a single fish during 1 min of treatment 3 (*T*_bottom_ = 6 °C). (**B, C**) Horizontal and vertical velocity components (*v*_x_, *v*_y_) derived from raw output (blue line) and after applying the spline smoothing (Lüthi et al., 2005). (**D**) Absolute 2D-swimming speed *V* based on smoothed velocity components. (**E**) Normalized swimming speed *V*_*norm*_ speed derived as *V*_norm_ = *V*(*t*) / *V*_mean_. The dashed line indicates the average swimming speed during the entire treatment duration (p1, p2, and p3 combined).

**Appendix 4—figure 2.**
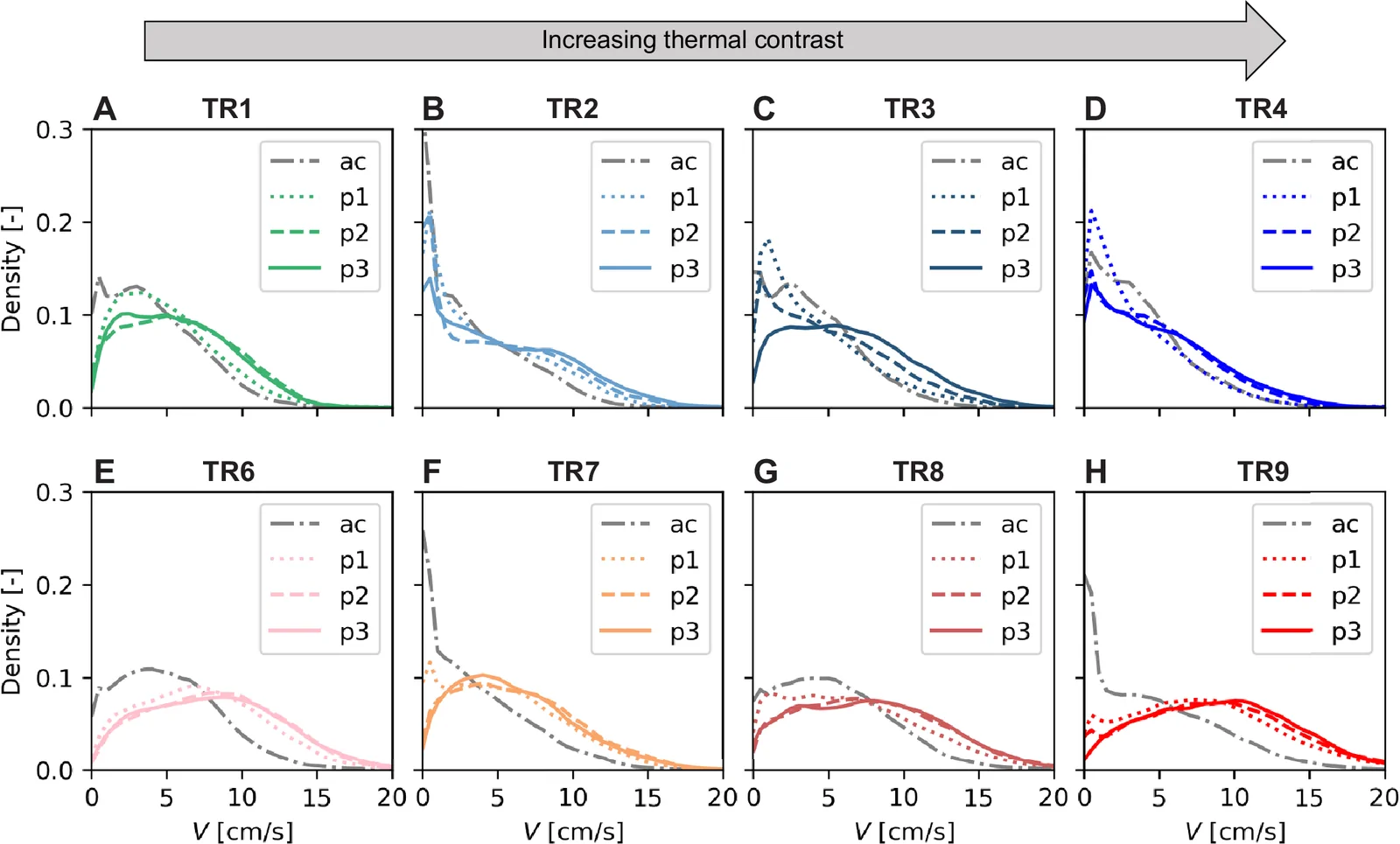
Probability density of absolute swimming speeds per experimental phase (ac: acclimation; p1, p2, and p3) and treatment. Cold treatments (**A–D**) and warm treatments (**E–H**). Each line indicates the normalized distributions of N = 6 min x 60 s x 24 fps x 20 fish = 172’800 observations.

**Appendix 5—figure 1.**
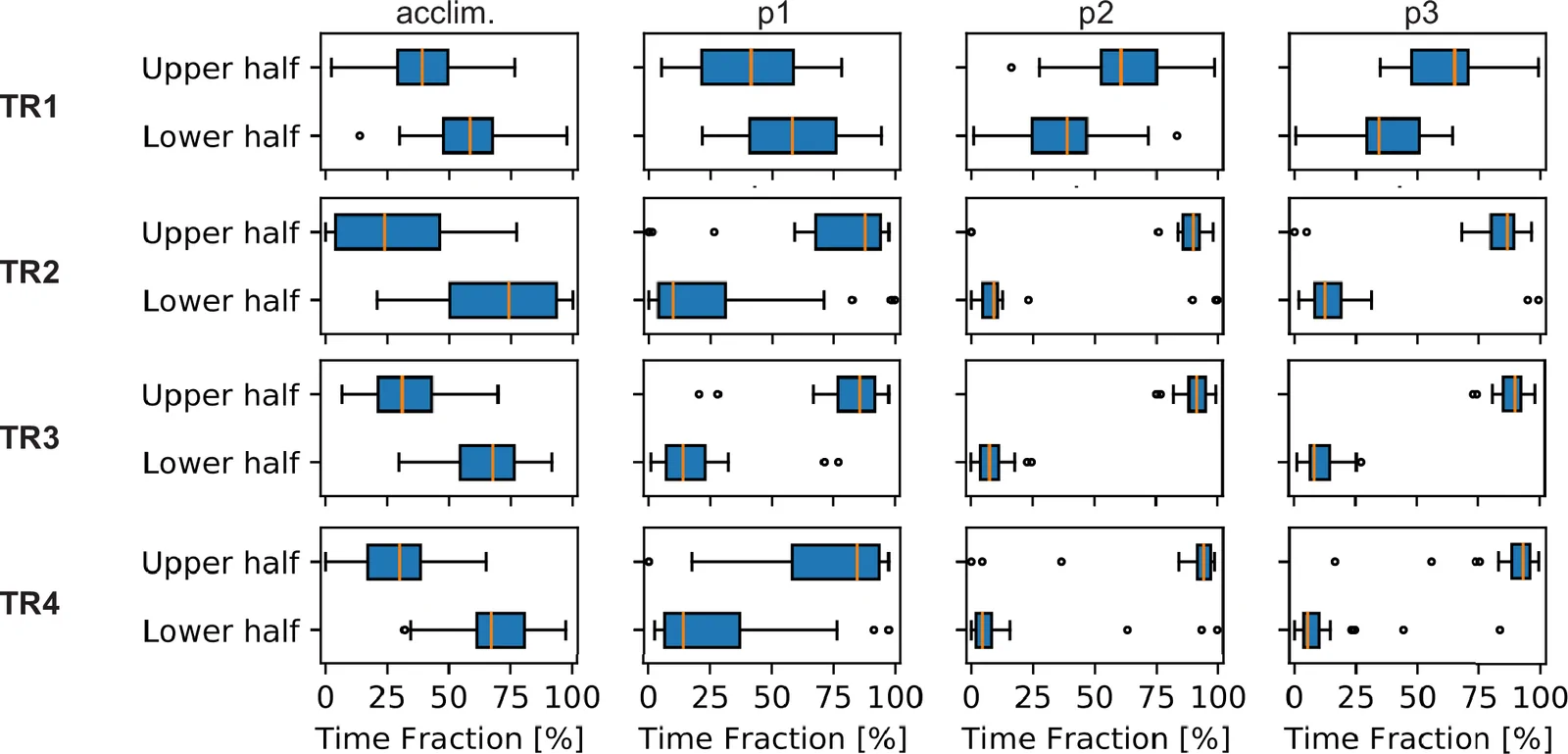
Cold treatments. Boxplots of time fraction spent in upper half (*Y*_norm_ = 0–0.5) and lower half (*Y*_norm_ = 0.5–1) of the tank across experimental phases (acclim.: acclimation; p1, p2, and p3) and treatments (TR1, TR2, TR3, and TR4).

**Appendix 5—figure 2.**
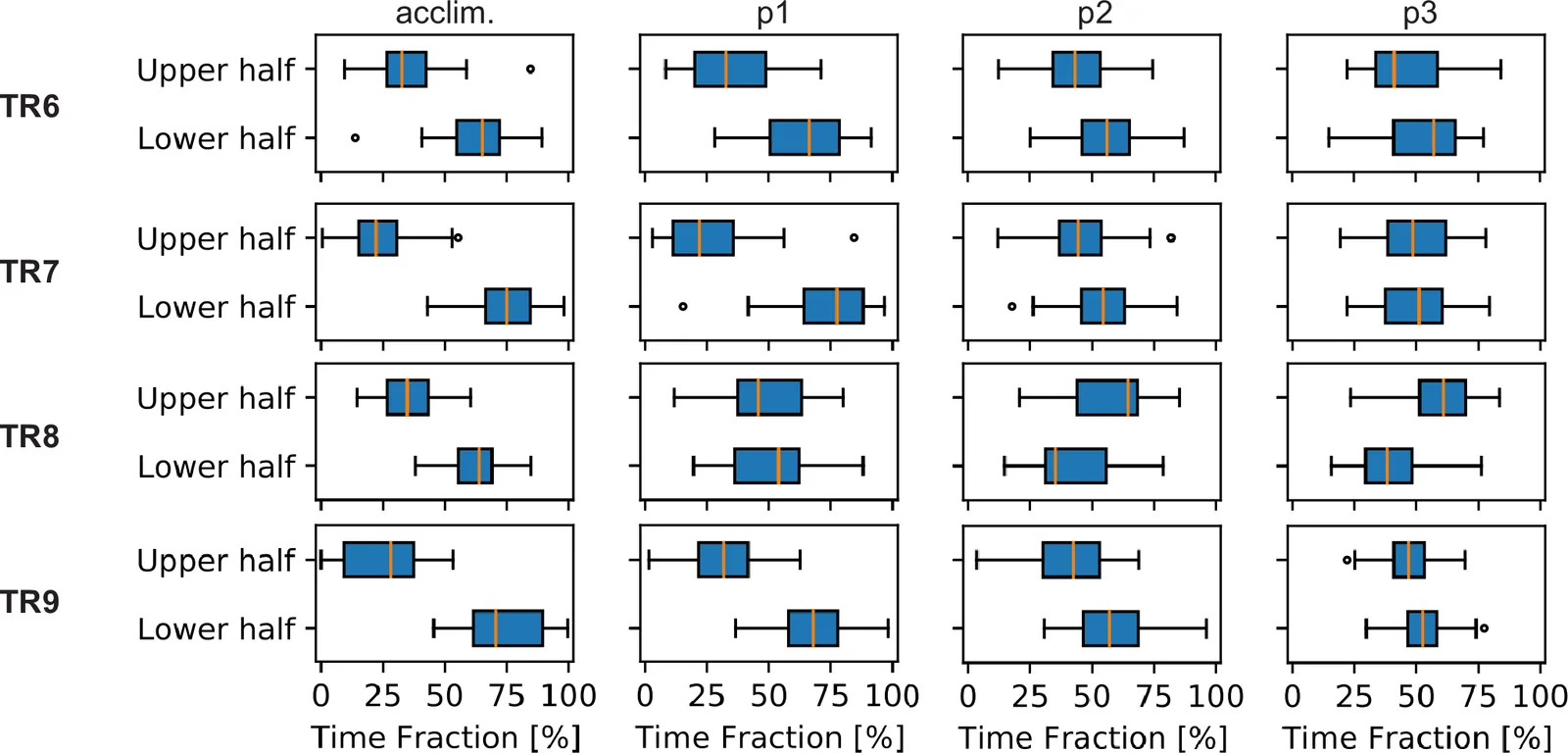
Warm treatments. Boxplots of time fraction spent in upper half (*Y*_norm_ = 0–0.5) and lower half (*Y*_norm_ = 0.5–1) of the tank across experimental phases (acclim.: acclimation; p1, p2, and p3) and treatments (TR6, TR7, TR8, and TR9).

**Appendix 6—figure 1.**
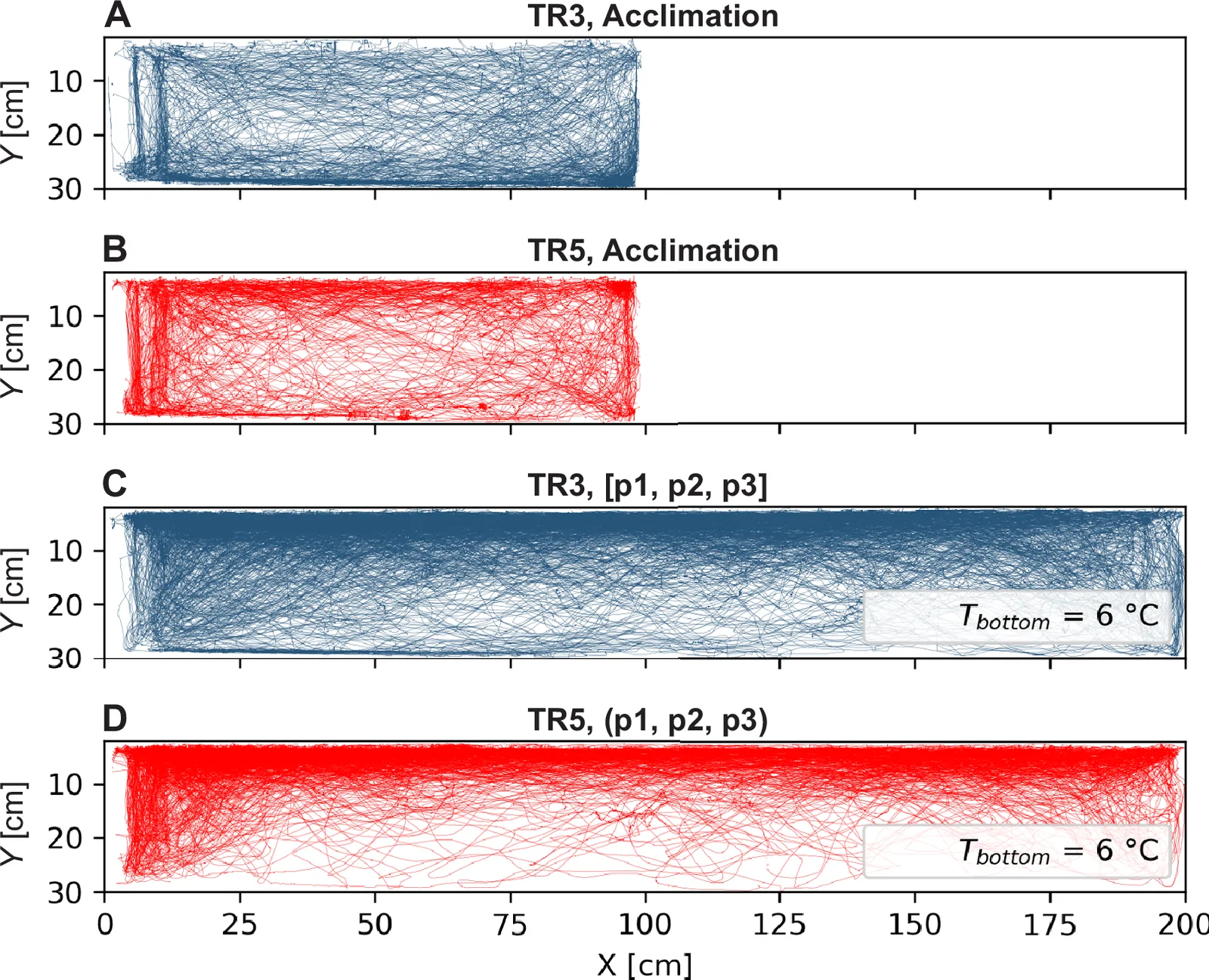
2D-swimming trajectories for TR3 (**A, C**) and its control without blue dye (TR5, **B, D**) during 6 min acclimation (**A, B**) and later experimental phases (p1, p2, and p3, **C, D**). Both treatments had identical temperature conditions (T_left_ = 12 °C; T_right_ = 6 °C).

**Appendix 6—figure 2.**
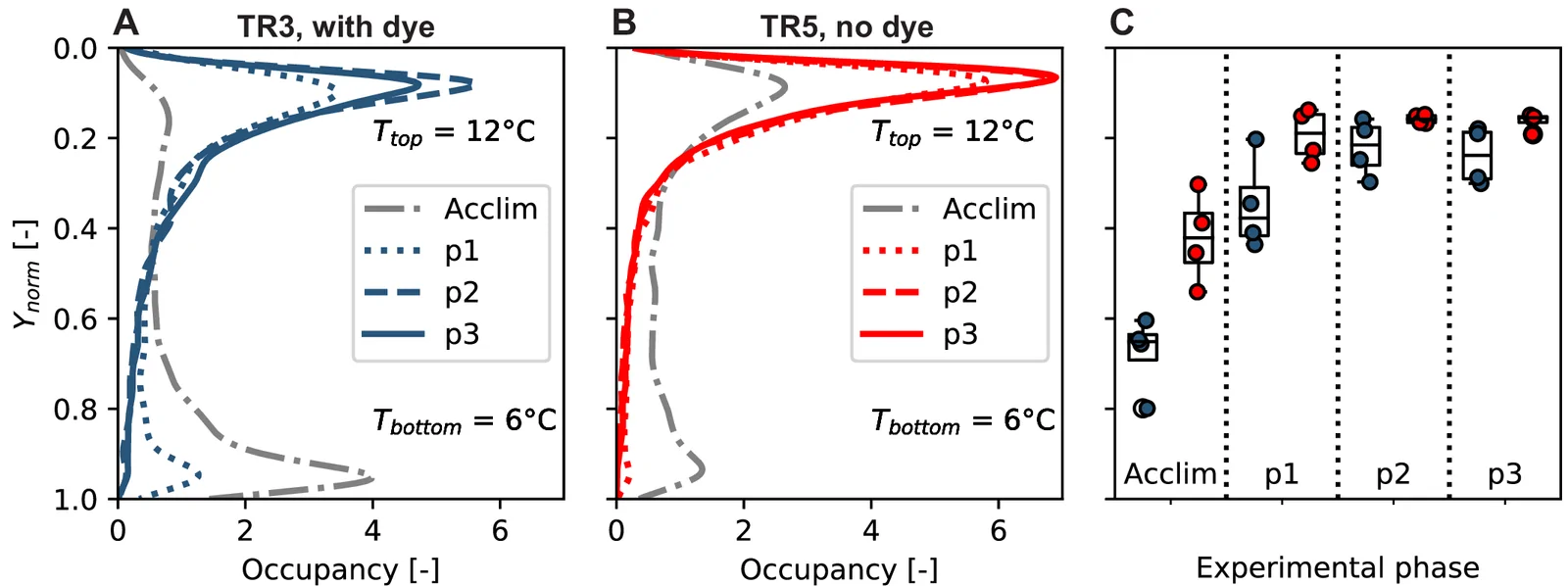
Vertical relocation in control experiment. (**A, B**) Probability density functions of fish depth during *experimental phases* (*acclim*.: acclimation; p1, p2, and p3) of the experiments for cold-water treatment 3 (TR3*, A, N = 16* fish) and its control without the addition of blue dye (TR5, B, *N* = 16 fish). Both cases had identical thermal conditions (T_bottom_ = 6 °C, T_top_ = 12 °C) above and below the thermal interface. The depth was normalized so that *Y*_norm_ = 0 is the water surface and *Y*_norm_ = 1 indicates the bottom of the tank. (**C**) Boxplots aggregated on the fish group level did not show significant differences with regards to the vertical relocation across experimental phases.

**Appendix 7—figure 1.**
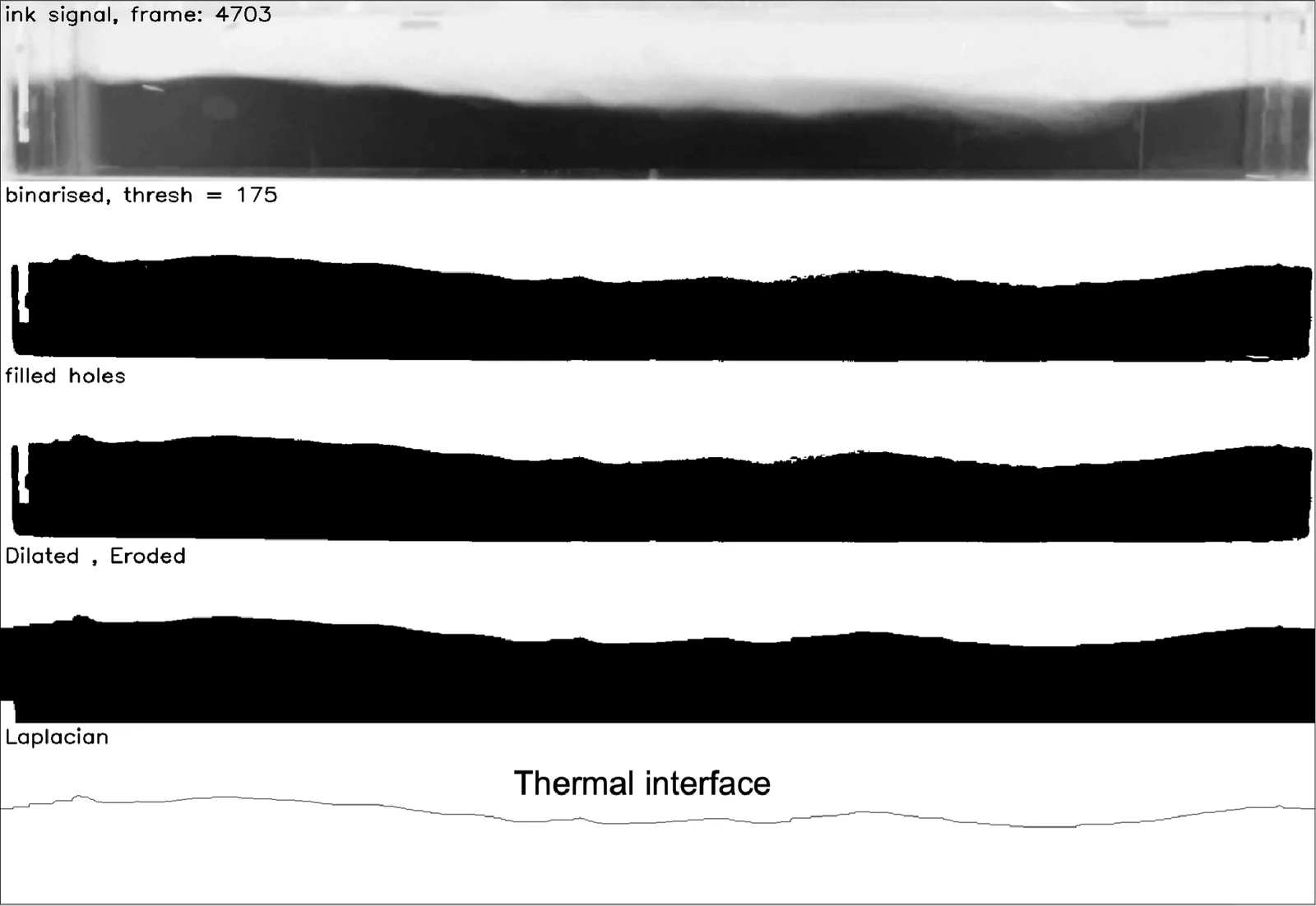
Image operations in Python (OpenCV) to derive the instantaneous position of the thermal interface. As the lock exchange flow is primarily a 2D phenomenon and the width of the tank is narrow (20 cm) as compared to its length (200 cm), the temperature interface was approximated as a 2D line (*X*(t),*Y*(t)). The interface coordinates were derived for each time frame using a custom-made image analysis pipeline (Python, OpenCV).

**Appendix 7—figure 2.**
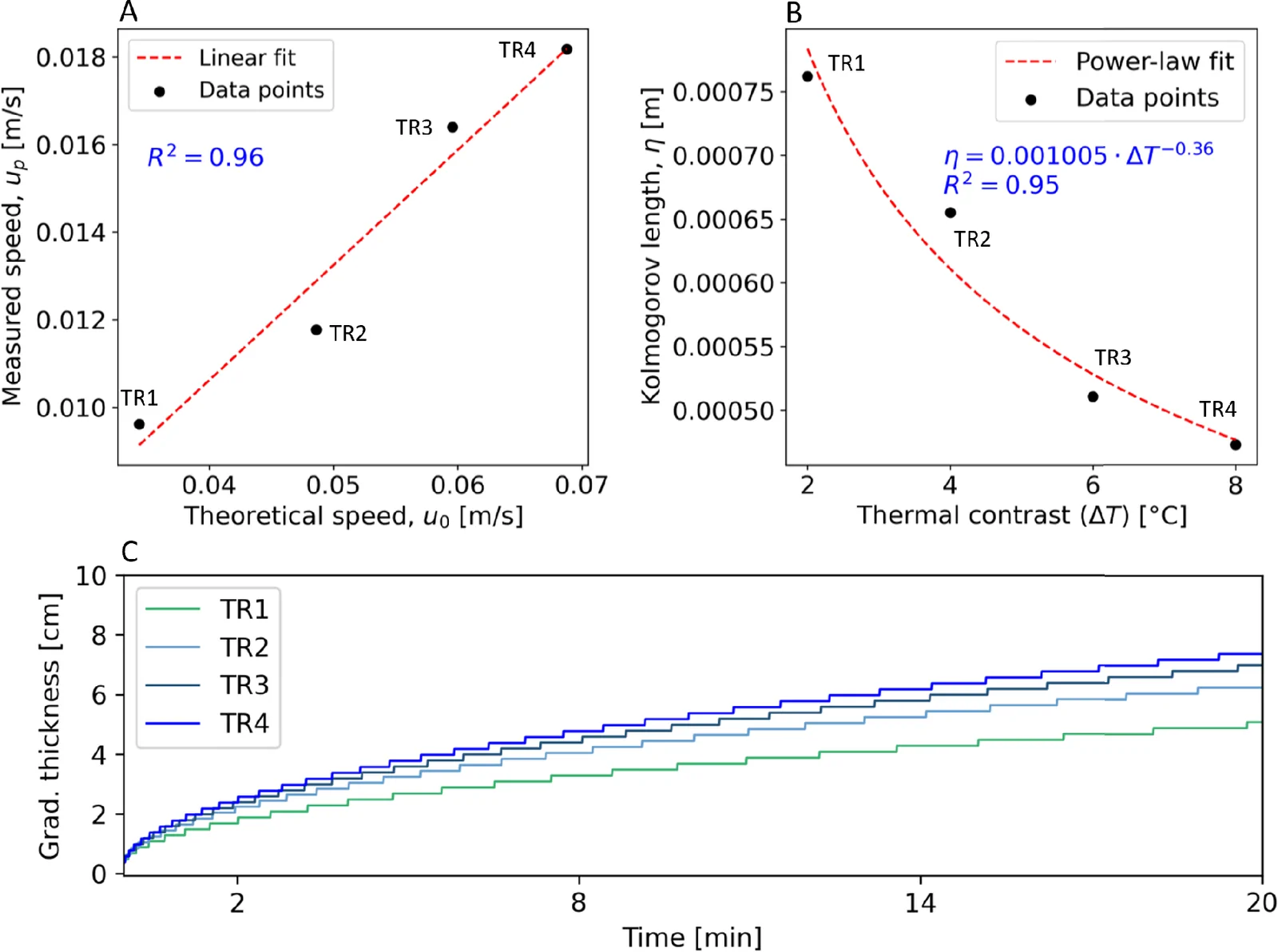
The theoretical large-scale gravitational velocity *u*_0_ plotted against the cold current propagation speed *u_p_* obtained from experimental videos of cold-water treatments (TR1–TR4). (**A**) A linear regression fit (red dashed line) is shown. (**B**) The Kolmogorov length *η* as a function of the temperature contrast Δ*T*. The black dots represent experimental data, while the red dashed line indicates a power-law fit. (**C**) Temporal evolution of the thermal interface thickness, influenced by turbulence and diffusion. A 2D numerical simulation was conducted over a 20 minute period, using an initial interface thickness estimated as $\delta\backsimeq 3.7 \, \eta$ (see Methods). The spatial domain was discretized into 1 mm^2^ grid cells (dx = dy = 0.001 m) and a constant thermal diffusivity of $\text {D}=1.43\text {e}-07 \, \rm m^2/s$ was assumed. Results indicate that, over the course of the experiment, the gradient thickness $\delta_k$ increased over time, rising to values larger than the length of the individual fish.

**Appendix 8—figure 1.**
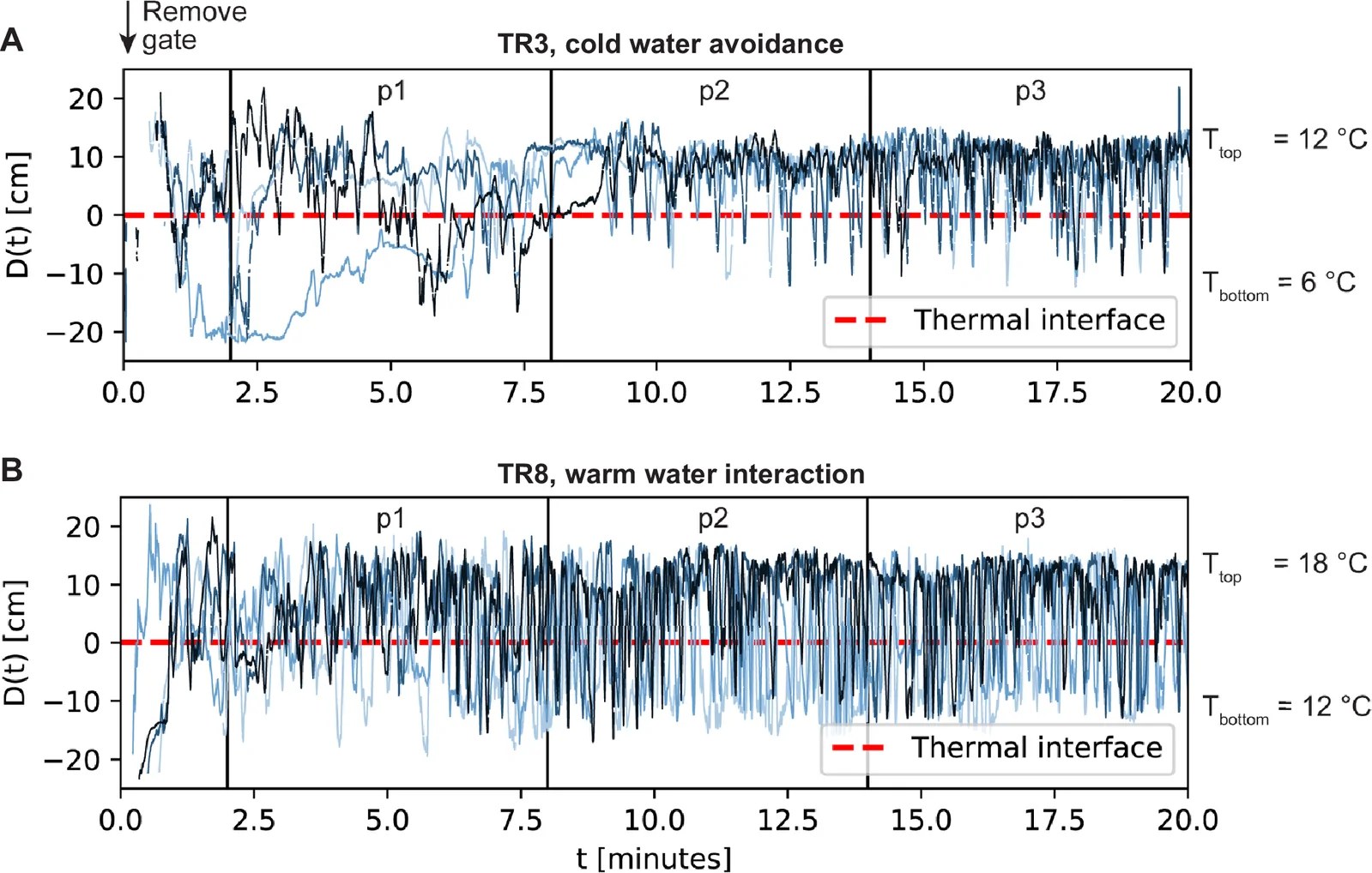
The temporal progression of the vertical distance to the thermal interface line, *D*(*t*), is shown for four individuals in an experiment of cold treatment 3 (TR3) (**A**) and warm treatment 8 (TR8) (**B**). The vertical gate was removed at time *t = 0*. Negative values of *D*(*t*) depict times when the center of gravity of the tracked fish was below the thermal interface; hence, in the colder water and vice versa. Each sign change of *D*(*t*) depicts an interface crossing. Data gaps within the first two minutes arise from the fact that the gravity current did not yet fully span the entire horizontal dimension of the tank (i.e. in this period was excluded from our analysis, fish may neither be located below nor above but laterally displaced from the thermal interface). Shortly after the current reaches the wall, maximum distances |D(t)| of up to 20 cm are possible (see also Appendix 13—Video 1). After the vertical stratification becomes stationary (t≈10 min), the interface is approximately located at half the tank depth (*h* = 14 cm), and values for |D(t)| are below 14 cm.

**Appendix 8—figure 2.**
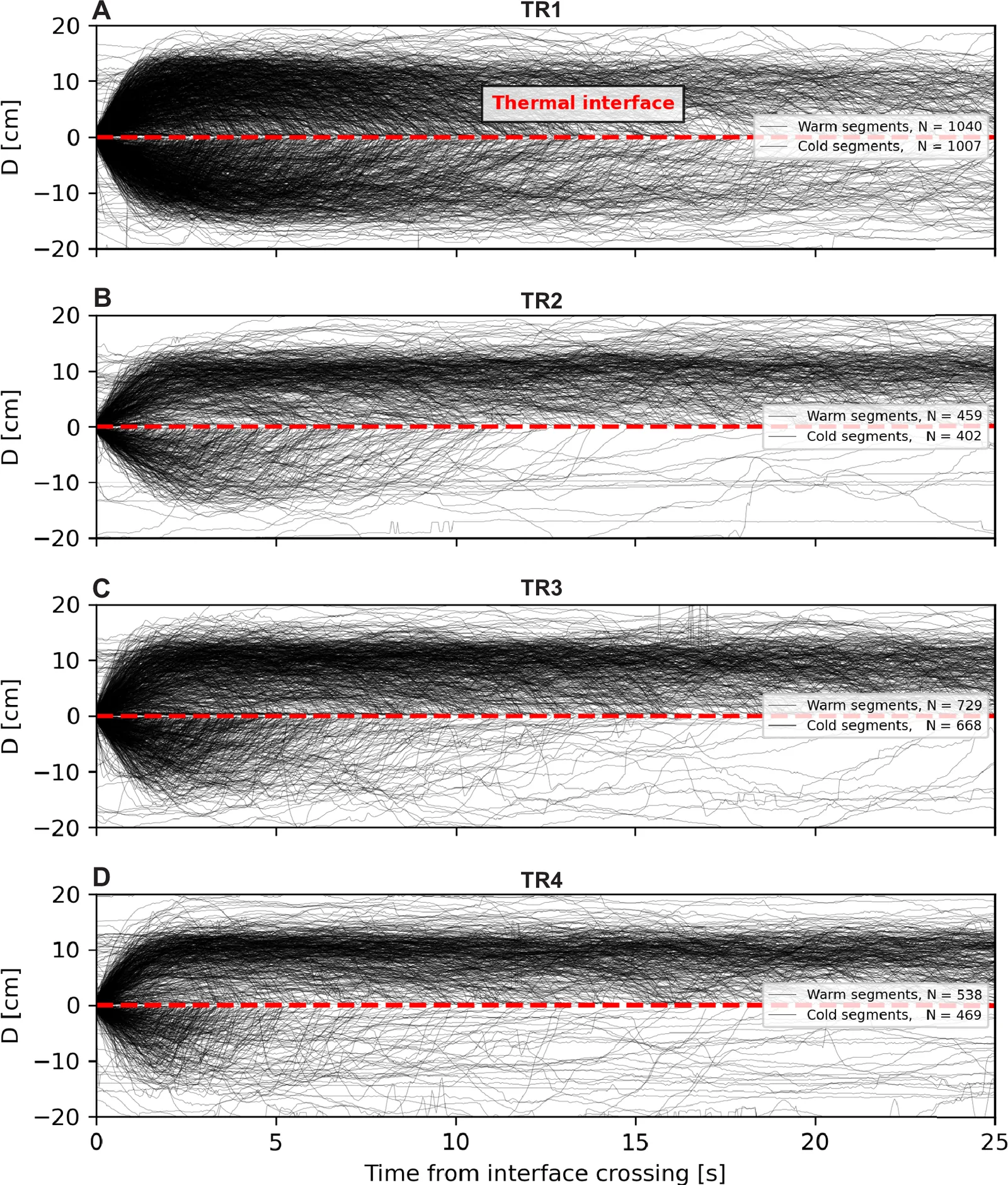
Cold treatments, TR1–TR4 (**A–D**). Temporal evolution of the fish’s instantaneous vertical distance to the thermal interface *D*(*t*) within the first 25 s for each cold- and warm-water interval for all cold treatments. At *t* = 0 fish crosses the thermal interface line upwards (warm segment; upper half) or downwards (cold segment; lower half). The end of an interval occurs when the interface line was crossed again in the opposite direction.

**Appendix 8—figure 3.**
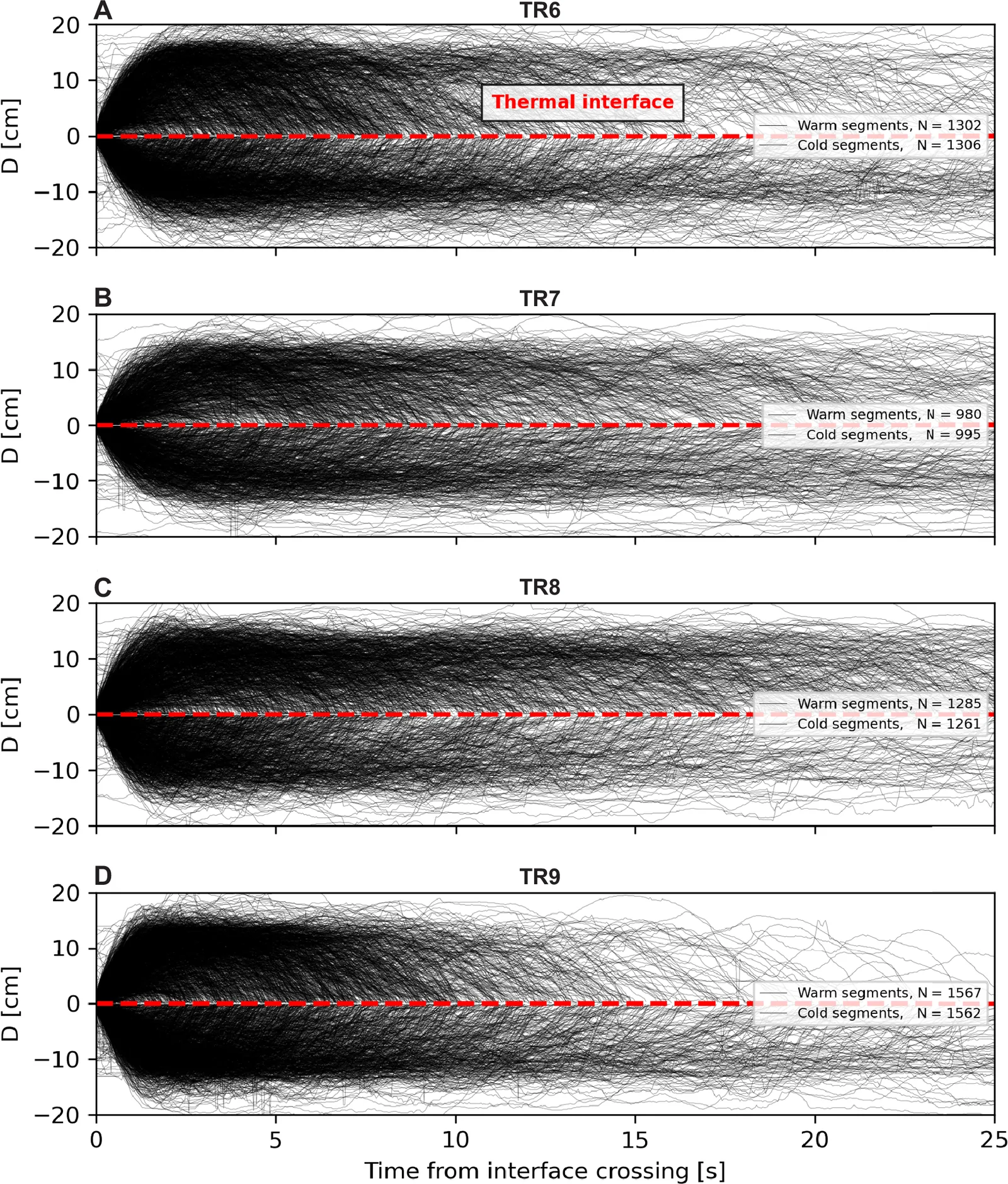
Warm treatments, TR6–TR9 (**A–D**). Temporal evolution of the fish’s instantaneous vertical distance to the thermal interface *D*(*t*) within the first 25 ss for each cold- and warm-water interval for all warm treatments. At *t* = 0, fish crosses the thermal interface line upwards (upper half) or downwards (lower half). The end of an interval occurs when the interface line was crossed again in the opposite direction.

**Appendix 8—figure 4.**
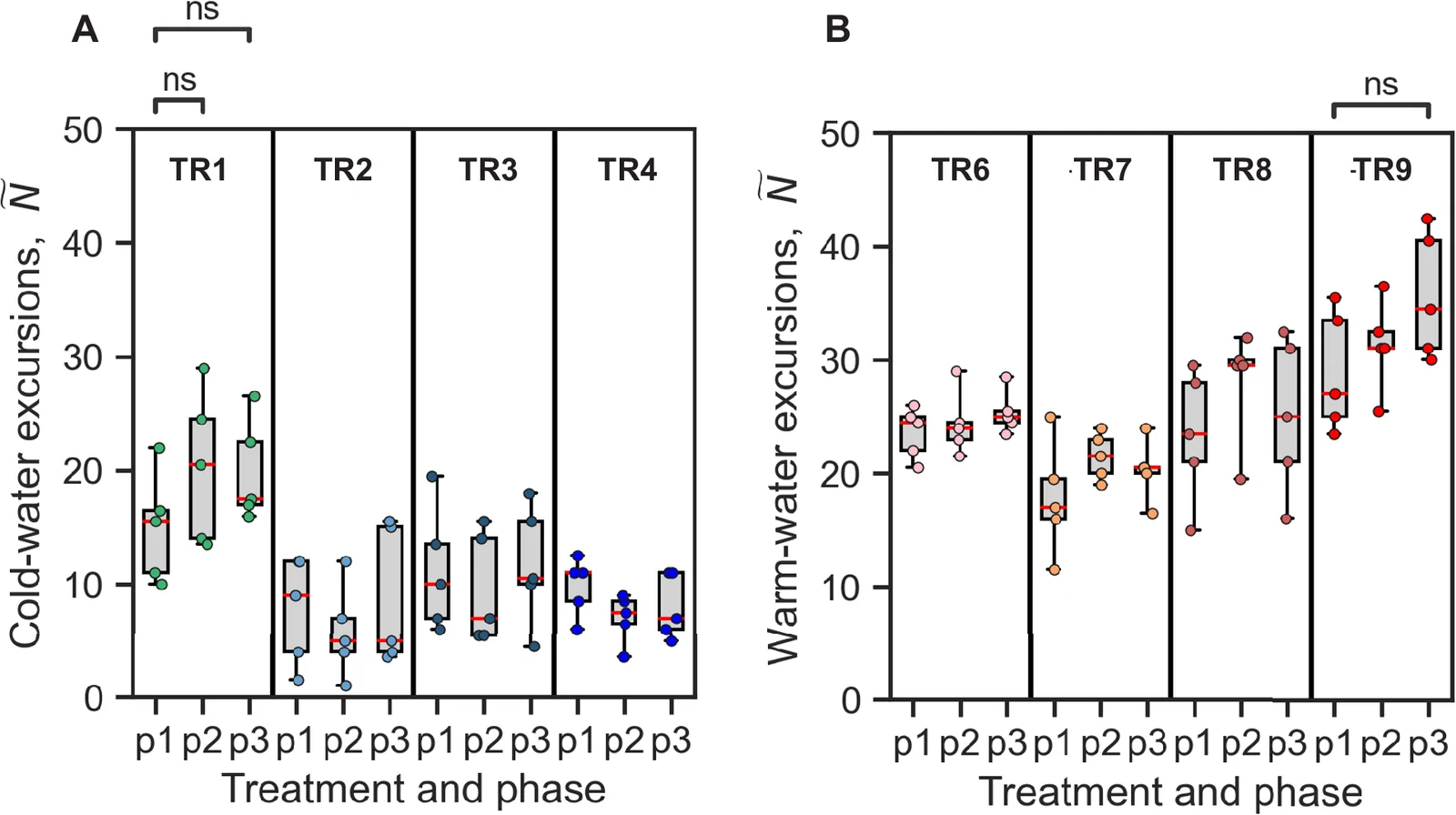
Number of performed cold-water excursions (**A**) and warm-water excursions (**B**) over time (p1–p3). Boxplots show the median; whiskers extend to the full data range, and the boxes are limited by the 25th and 75th percentiles. Each dot represents the median across four individuals tested simultaneously.

**Appendix 8—figure 5.**
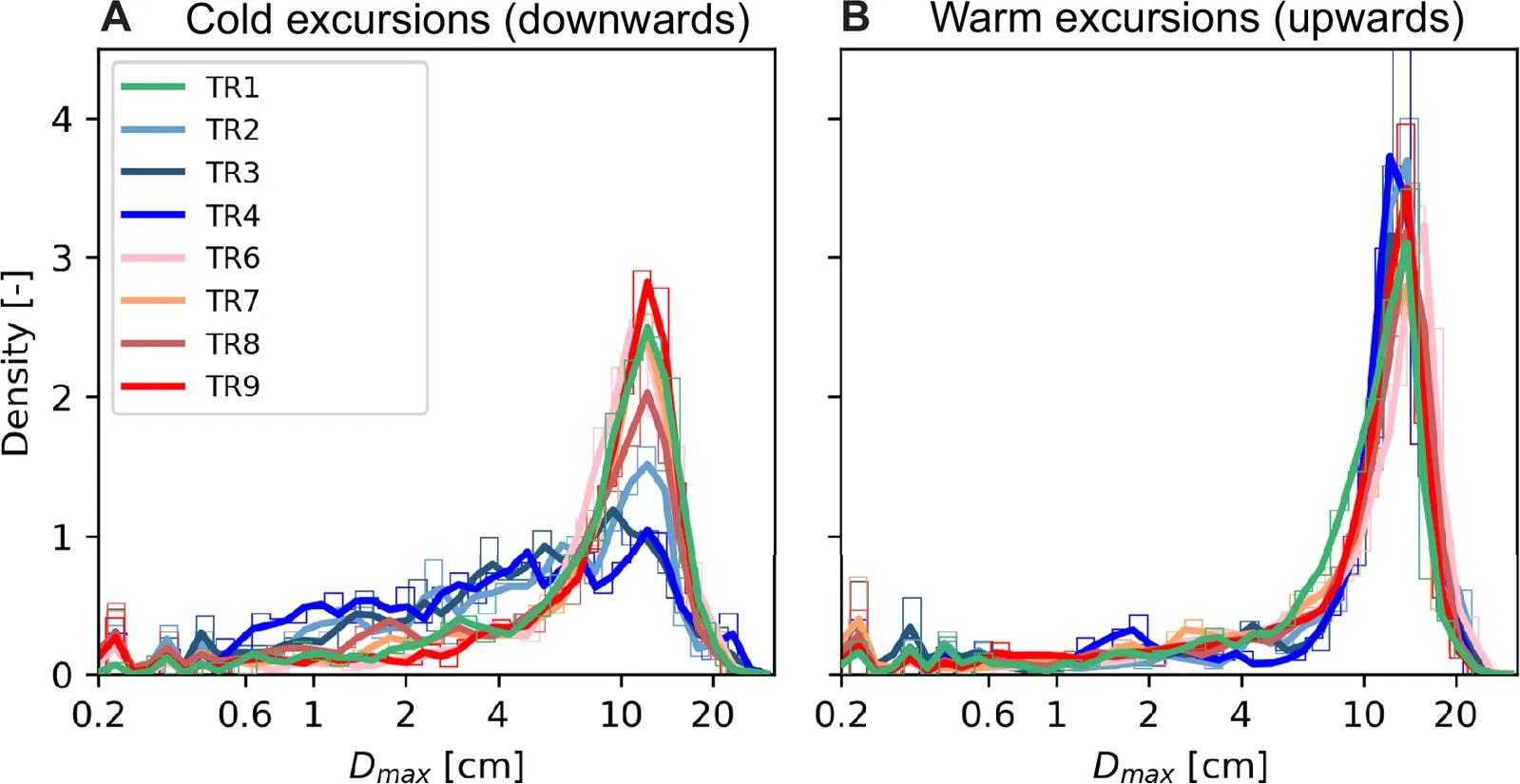
Probability density function of maximum distance to interface line *D*_max_ for cold (**A**) and warm (**B**) excursions for all treatments. Underlying normalized histograms are shown in thin colored lines.

**Appendix 8—figure 6.**
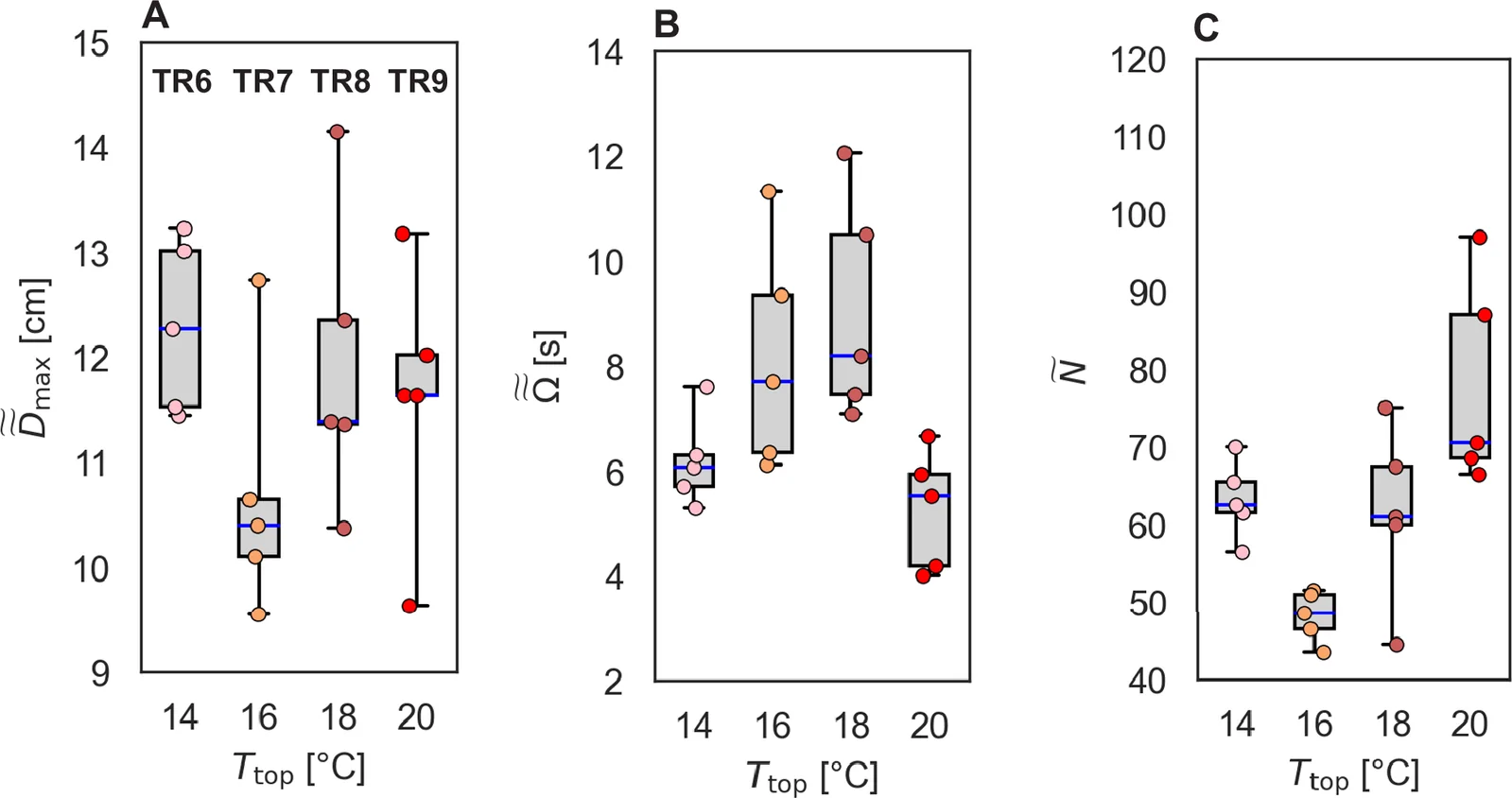
Warm-water excursions during warm treatments (TR6-TR9). For each quantity, the median of four individuals (tested simultaneously) was considered; hence, each dot represents a single experiment. (**A**) Boxplots of the maximum (upwards) distance to the interface of warm-water excursions. The median of $\boldsymbol{D}_{\max }$ was applied to all excursions performed by an individual fish and for all phases (p1, p2, and p3). (**B**) Boxplots of the median duration of cold-water excursions for fish. The median of $\boldsymbol{D}_{\max }$ was applied to all excursions performed by an individual fish and for all phases (p1, p2, and p3). Each dot represents a single experiment. (**C**) Boxplot of the median number of warm-water excursions (upwards) for fish groups across warm treatments and experimental phases (p1, p2, and p3). Each dot represents the median response of four fish in a single experiment. Blue lines indicate the median; boxplots are limited by the 25th and 75th percentiles. Whiskers extend to the full range of observations.

**Appendix 9—figure 1.**
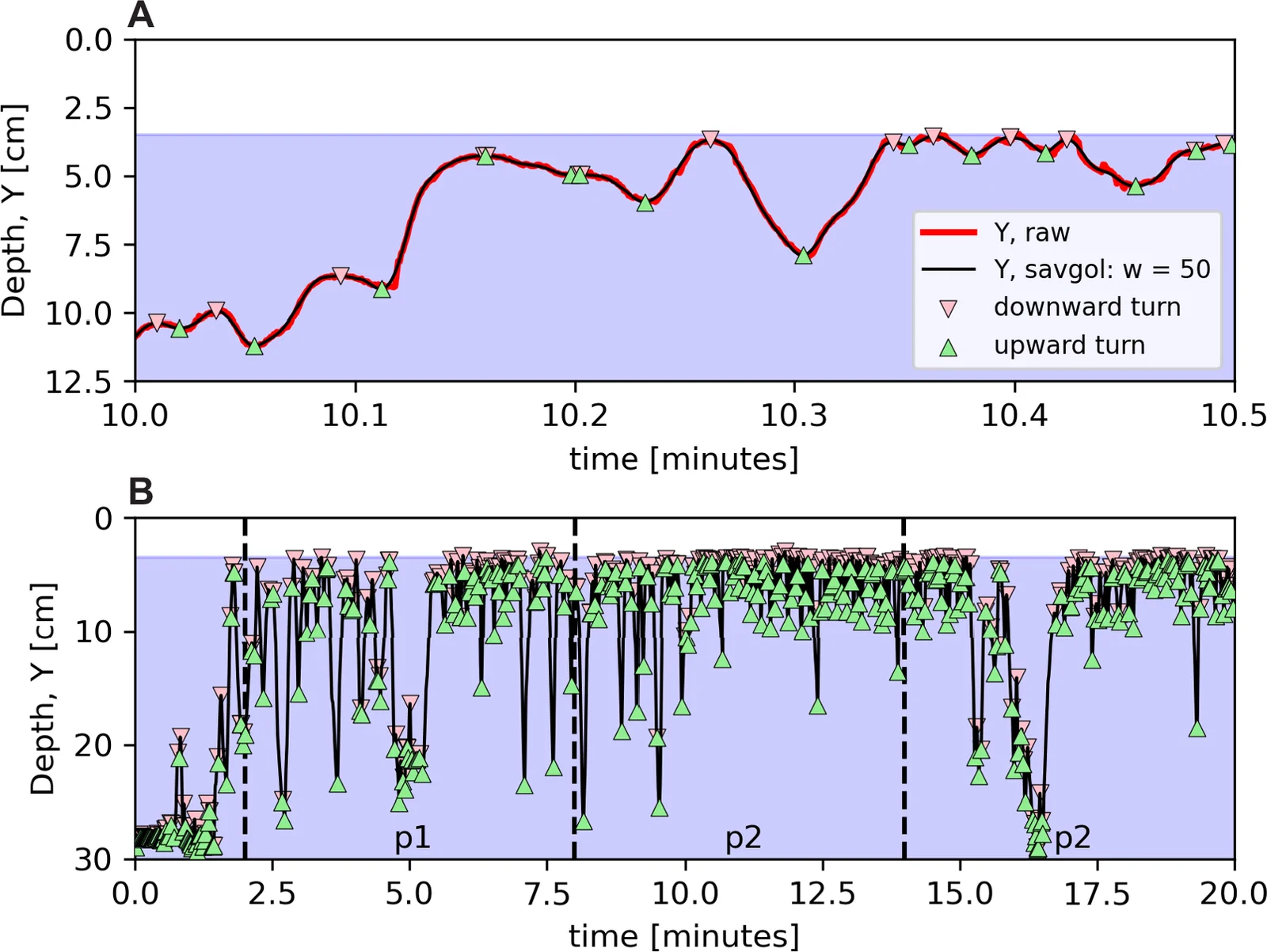
Derivation of vertical turning points. (**A**) To smooth the fish’s vertical position signal (red line), a fifth-order polynomial was fitted to a moving window of 50 consecutive time points (black line). This is a standard practice in particle tracking to filter out noise (e.g. Lüthi et al., 2005). Therefore, the SAVGOL function (python: scipy.signal.savgol_filter) was applied on the raw output (black line) from the tracking software. Upward turning (green triangles) was detected as a sign change (from negative to positive) of the derivative of the smoothed signal. Downward turning (red triangles) was detected via sign change (from positive to negative). (**B**) Occurrence of turning points after gate removal (at *t* = 0) for an individual fish in cold treatment (TR3).

**Appendix 9—figure 2.**
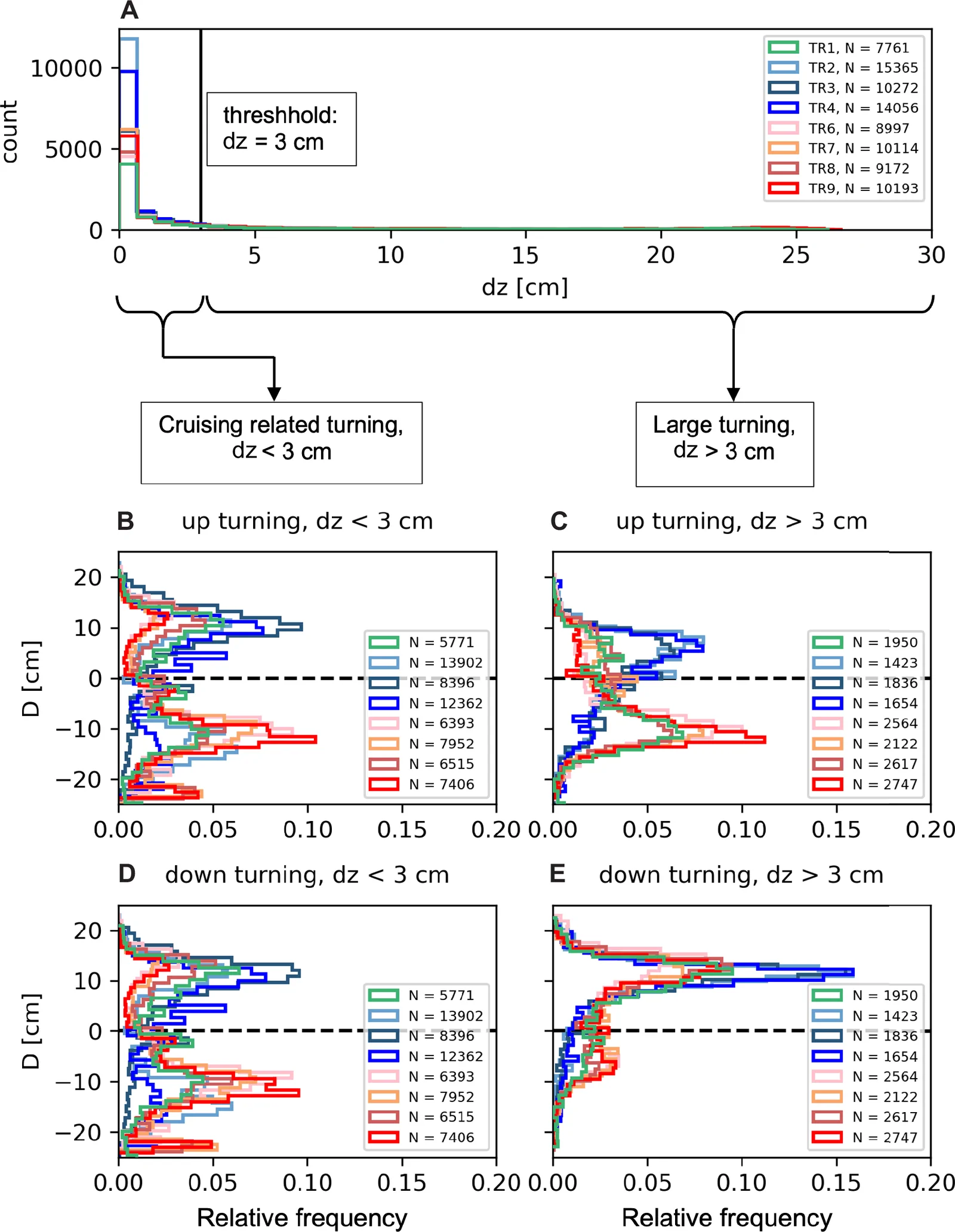
Occurence frequency of vertical turning points. (**A**) Normalized histogram of the vertical distance dz between all consecutive turning points for all treatments (depicted by colors). A threshold of dz = 3 cm was used to differentiate turning events that result in small, cruising-related vertical displacements (**B, D**) from vertical turns that resulted in large vertical displacement (dz >3 cm) (**C, E**).

**Appendix 9—figure 3.**
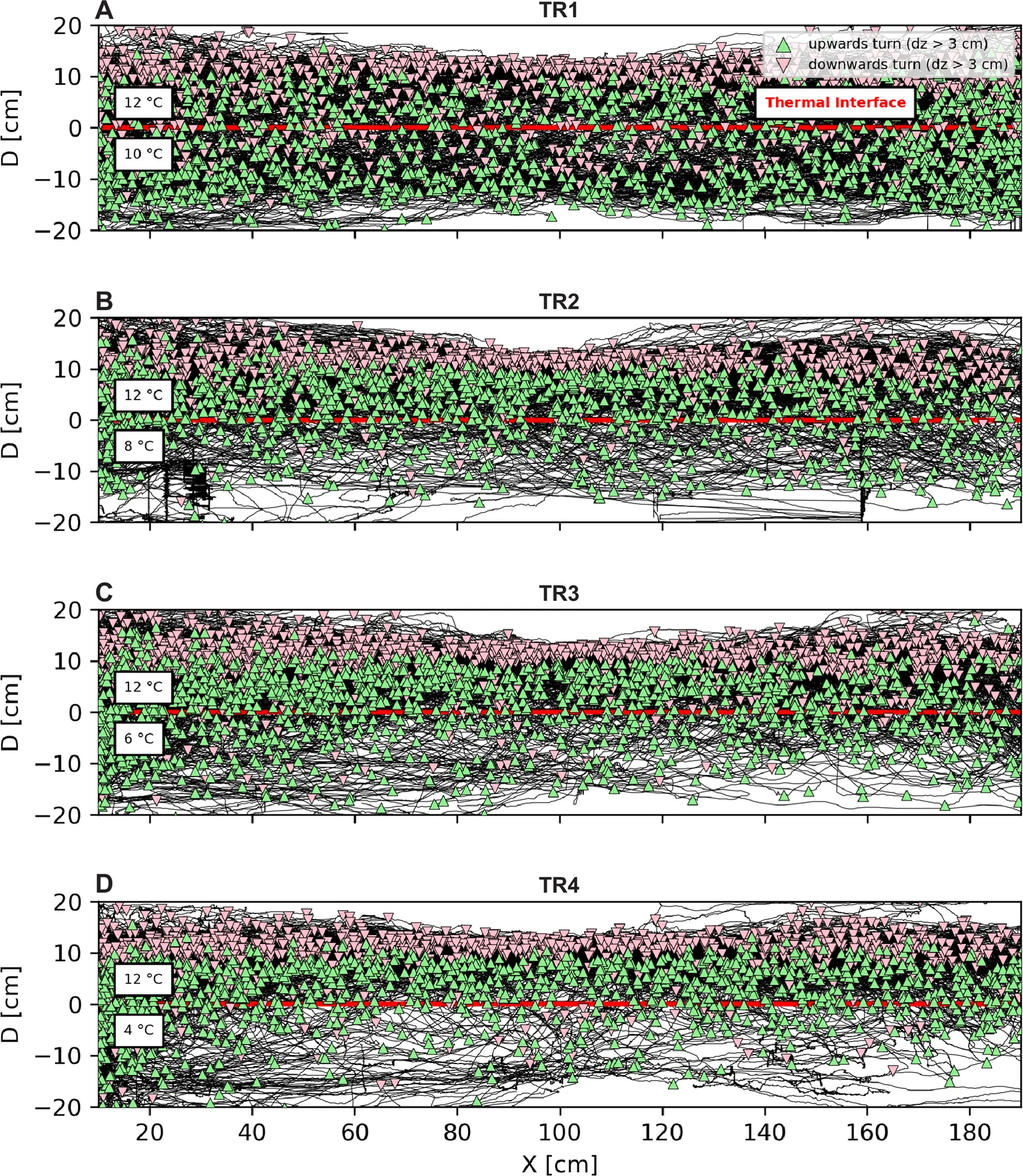
Cold treatments. Swimming trajectories (black lines) relative to the thermal interface (red line) for all fish across cold treatments (TR1 – TR4, **A–D**). The horizontal axis depicts the horizontal position in centimeters; the vertical axis depicts the instantaneous distance from the thermal interface. For *D* > 0, fish is located at a distance *D* [cm] above the interface and for *D* < 0, the fish is located below the temperature interface. Red and green triangles depict large downward and upward turns, respectively (see Appendix 9—figure 2 for derivation).

**Appendix 9—figure 4.**
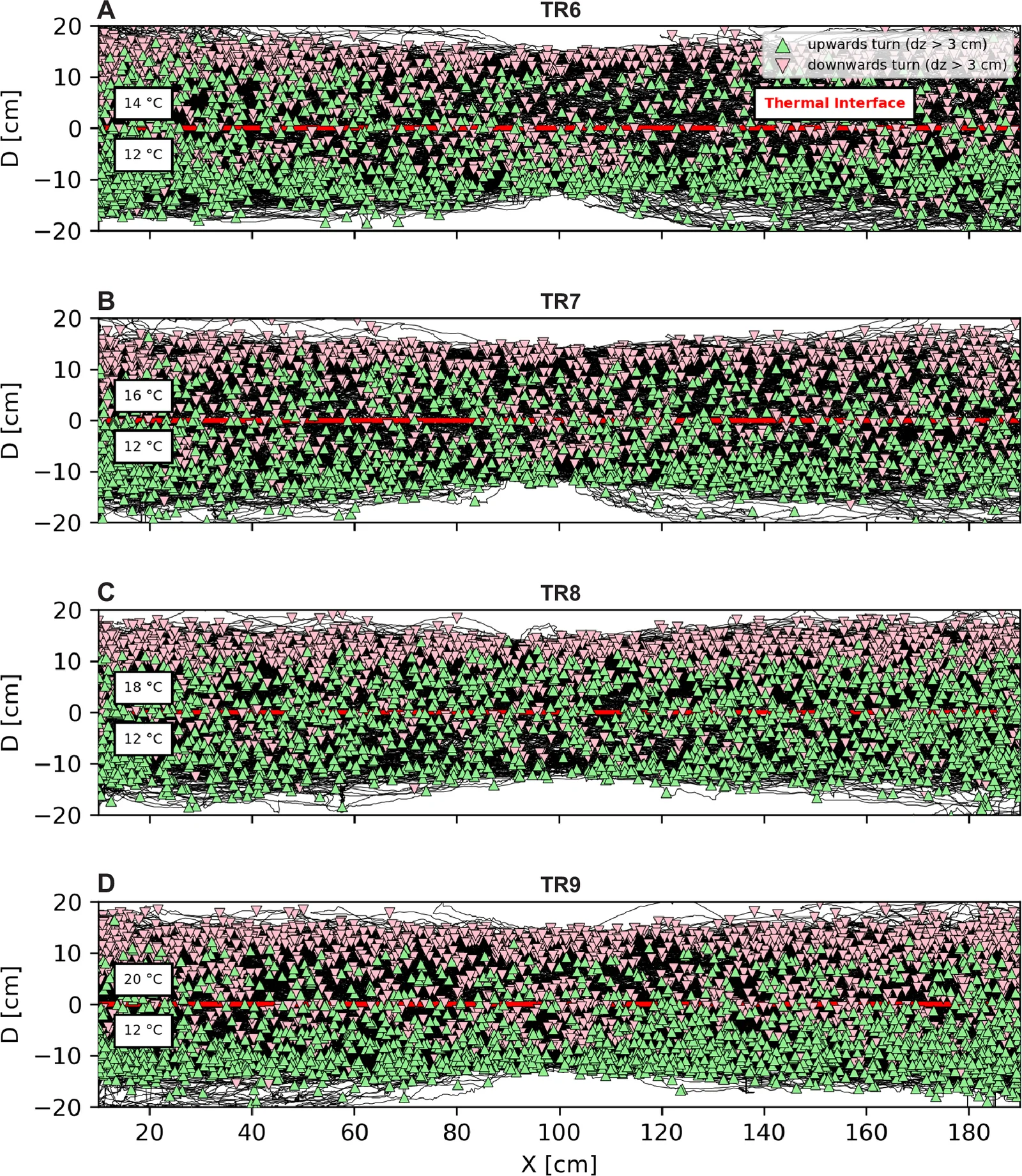
Warm treatments. Swimming trajectories (black lines) relative to the thermal interface (red line) for all fish across cold treatments (TR6–TR9, **A–D**). The horizontal axis depicts the horizontal position in centimeters; the vertical axis depicts the instantaneous distance from the thermal interface. For *D* > 0, fish is located at a distance *D* [cm] above the interface and for *D* < 0, the fish is located below the temperature interface. Red and green triangles depict large downward and upward turns, respectively (see Appendix 9—figure 2 for derivation).

**Appendix 9—figure 5.**
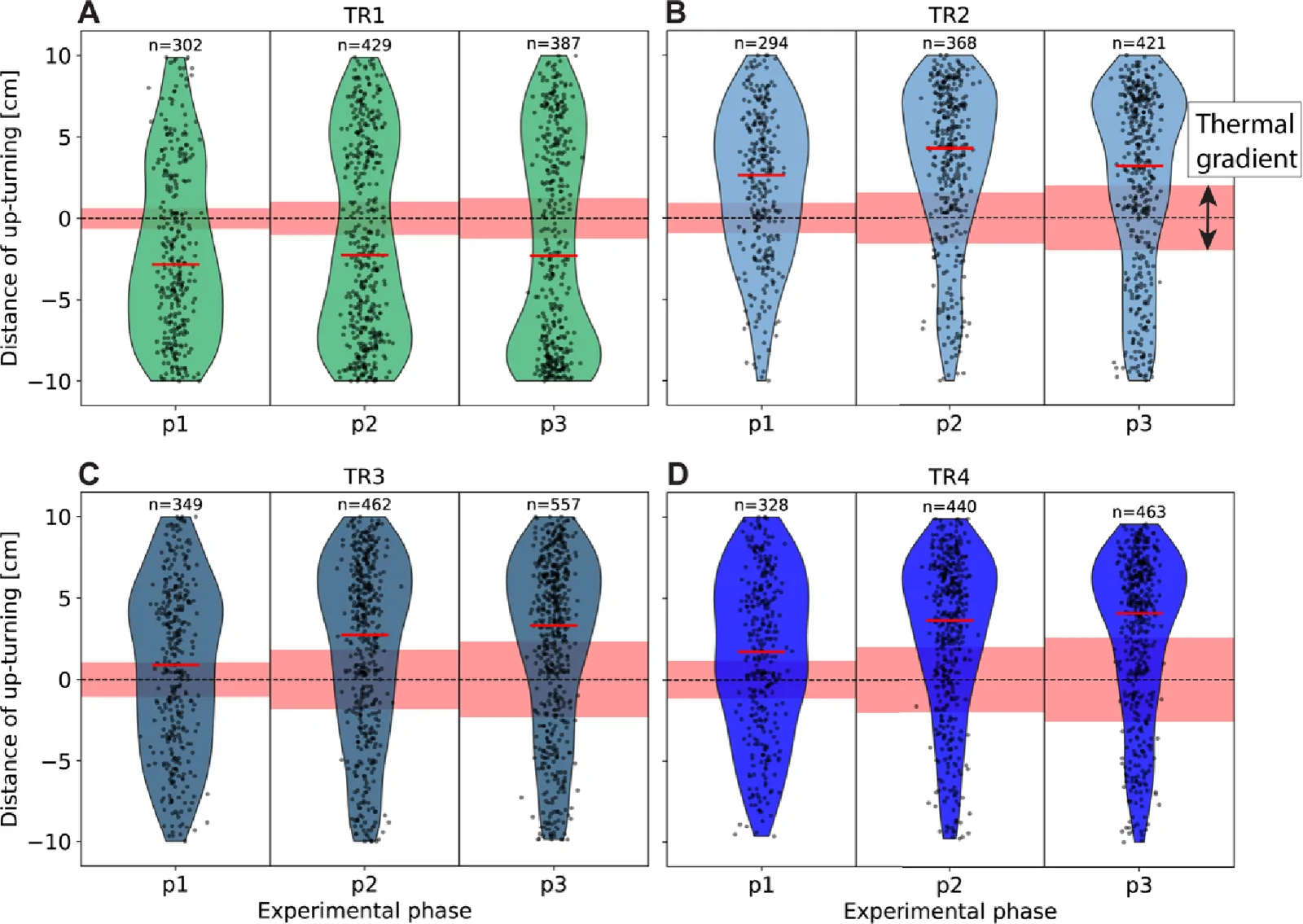
Violin plots showing the vertical distance from the thermal interface at which up-turning events occurred during cold-water treatments (TR1–TR4, **A–D**). Each black dot represents a single up-turning event, and the red line indicates the median distance. Data are grouped by experimental phase (p1–p3). The dashed line denotes the position of the thermal interface (0 cm), and the red shaded area indicates the estimated thickness of the vertical thermal gradient (see Methods).

**Appendix 10—figure 1.**
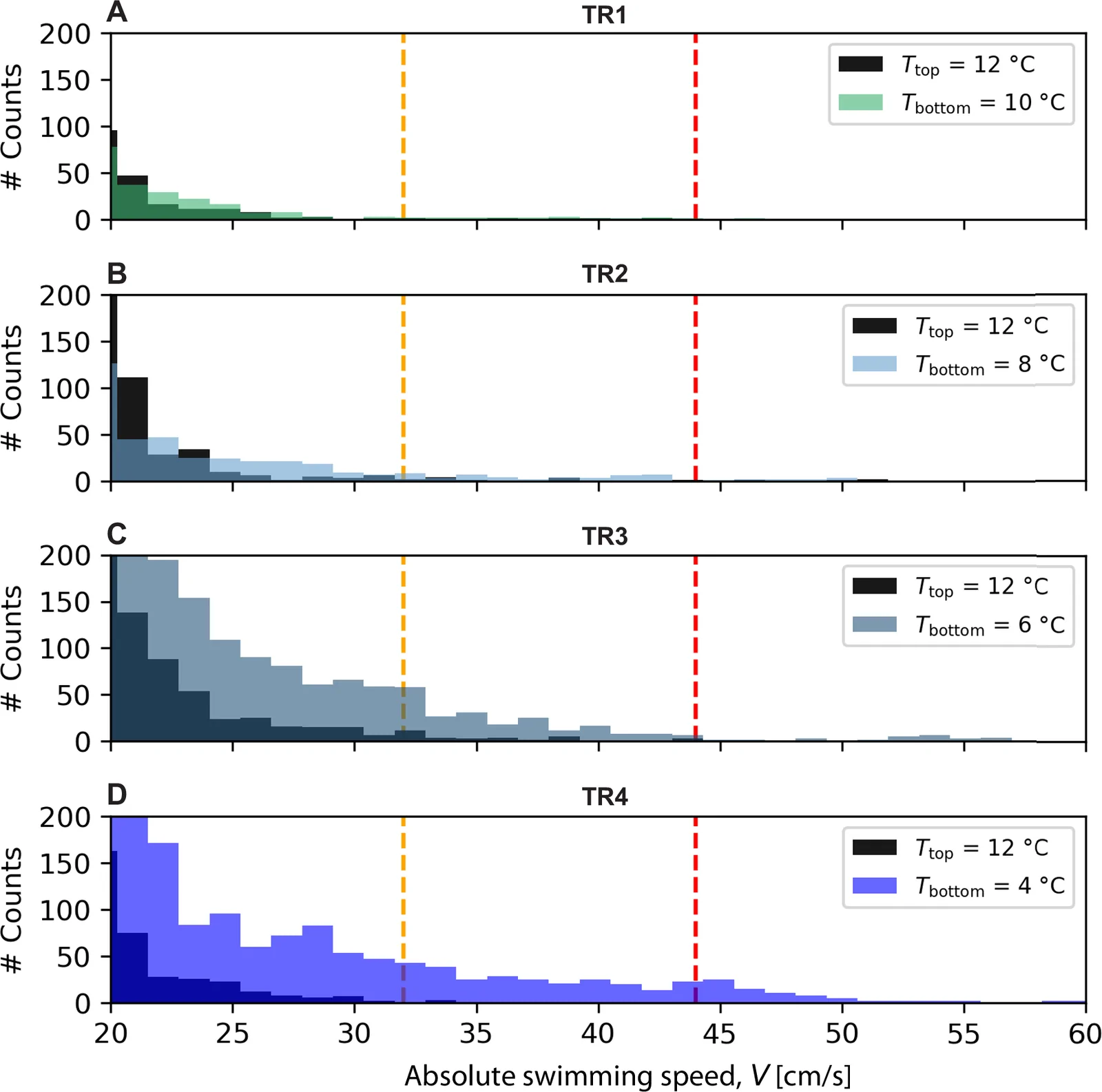
Extreme swimming speeds above *V* = 20 cm/s for TR1–TR4 (**A-D**). Black histograms show all observations above the thermal interface (*T*_top_ = 12 °C). Colored histograms display all observations below the thermal interface (in colder water). Dashed vertical lines depict literature thresholds for burst swimming speeds of rheophilic fish based on temperature (orange line: *T* = 12°; red line: *T* = 4 °C), body length (3.6 cm), and swimming durations (5 s) as proposed by Ebel, 2014 (see above). High swimming speeds were most frequent during cold-water occupation in colder treatments (TR2–TR4).

**Appendix 11—figure 1.**
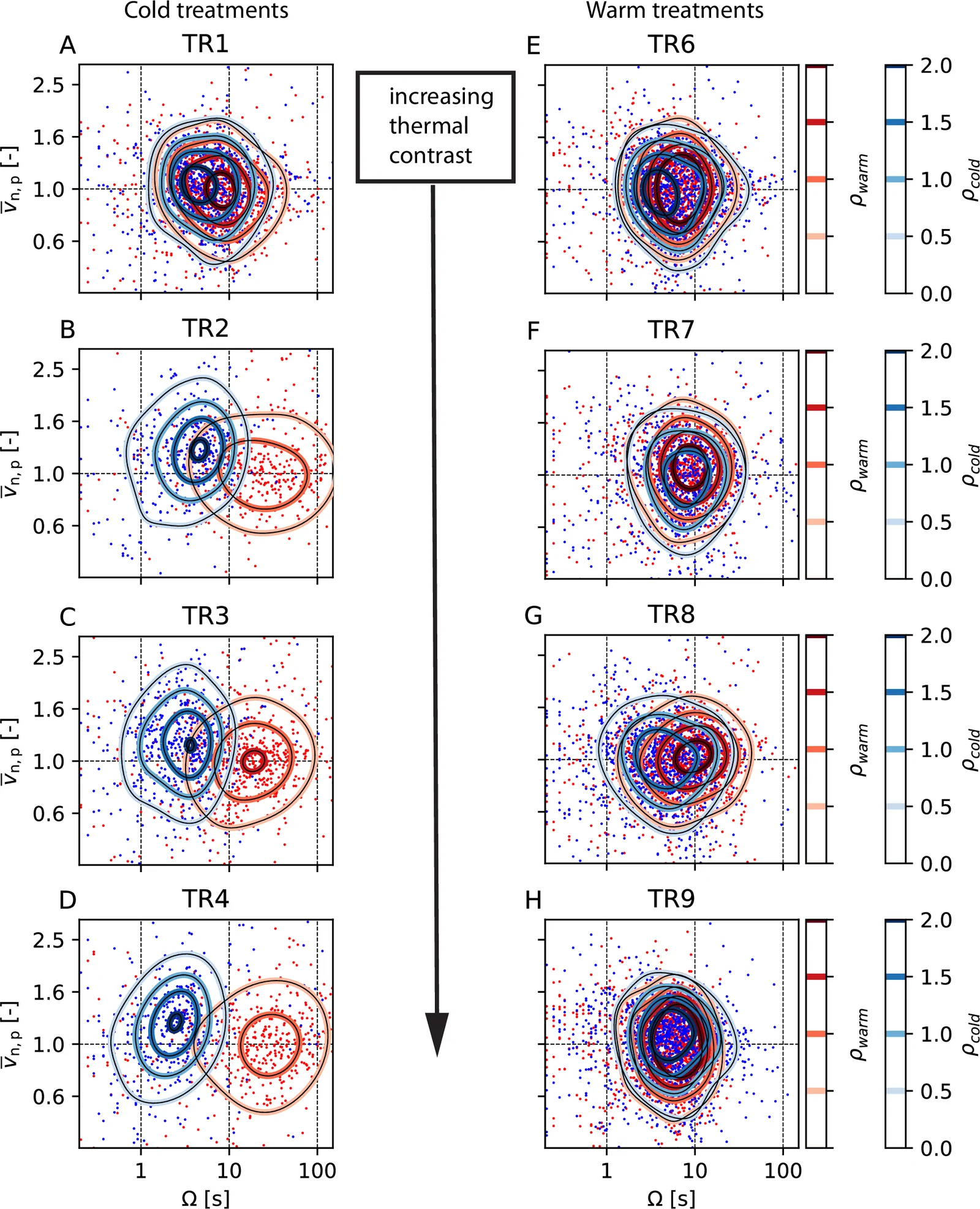
Normalized swimming speed $\bar{v}_{\text {n,p}}$ averaged over excursions plotted as a function of the duration Ω of each cold (blue points) and warm (red points) water excursion for all cold (**A–D**) and all warm treatments (**E–H**). To control for temporal trends, speed was standardized by the average swimming speed of each individual within each phase. Fitting a 2D Kernel density estimator to the point clouds (red and blue lines) reveals for cold treatments with $T_{\text {Bottom}}\leq {8\,}^{\circ} {\rm C}$ (TR2–TR4, **B–D**), clusters separate with respect to swimming speed (y-axis) and duration (x-axis).

**Appendix 12—figure 1.**
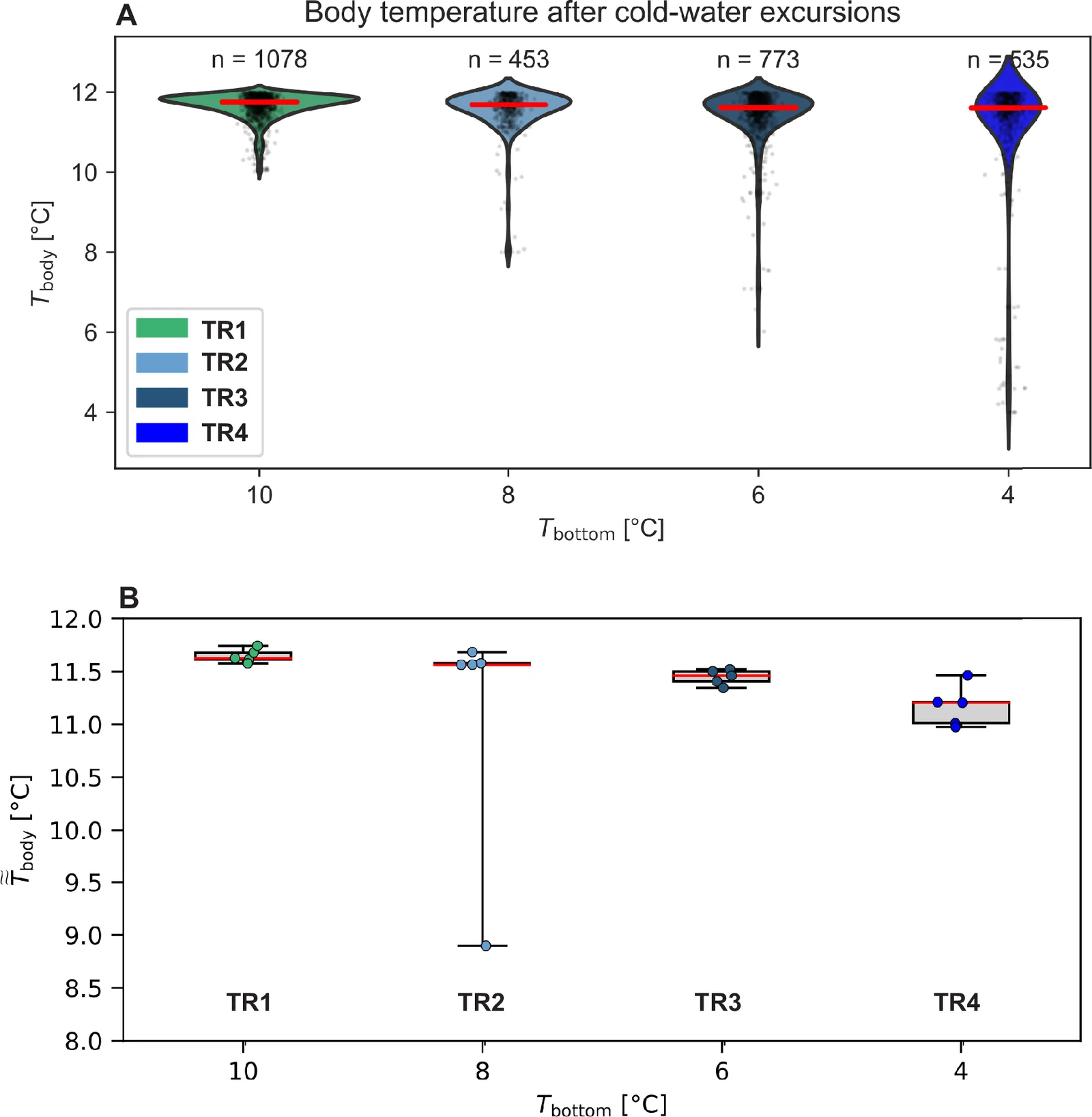
Simulated body temperature after cold-water excursions. (**A**) Violin plots of simulated body temperature *T*_body_ after each performed cold-water excursion for cold-water treatments (TR1-TR4). Each dot is one excursion, the red line indicates the median, and *n* is the number of performed excursions by 5x4 = 20 fish within each treatment. The model (see *Methods*) assumed fish body mass of m = 4 gramm, initial body temperature of *T*_0_ = 12 °C and a cooling rate coefficient of k = 1.373 min-1. (**B**) Box plot of the median body temperature ($\overset{\sim}{\overset{\sim}{T}}_{\mathrm{body}}$) after cold-water excursions for each experimental replicate. The red line indicates the median across experimental replicates.

## Data Availability

The Python scripts used for the analyses are available through a public GitHub repository https://github.com/naroberto/Thermal_paper/, copy archived at Naudascher, 2026. The generated videos for fish-tracking can be accessed in an open access archive (https://doi.org/10.3929/ethz-b-000660506). The following dataset was generated: StockerR NaudascherR 2024Trout parr exhibit an acute behavioral response to heterothermal environmentsETH Library research collection10.3929/ethz-b-000660506PMC1366812042873085

## Additional files

MDAR checklist

## Appendix 1

### Gravity currents and definition of treatments

We contrasted water at the acclimation temperature (12 °C) with four cold treatments (TR1–TR4) and four warm treatments (TR6– TR9); hence, we varied the incoming water temperature in both directions (Appendix 1—table 1).

The difference in specific weight between warmer and colder fluid provided the driving force for the gravity currents that unfolded during the first 2 min after gate removal. After impounding at the wall, the thermal interface separated the cold water in the bottom from the warmer water in the top of the tank across the entire length $L\, =\, 2\, \mathrm{m}$ of the tank. However, the thermal interface was not stationary in time. Continuously dissipating the initial energy of the system, the interface was swaying between the left and right side of the tank with diminishing movement over time (Figure 1—figure supplement 4, Appendix 1—figure 1). For all treatments, its vertical position varied more strongly across X and in time during p1 and decreased for p2 and p3. The maximum movement velocity was small ($v_{\mathrm{interface}}\, \approx \, 0.1\, \mathrm{cm/s}$) when compared to the typical swimming speed of the tested fish ($v_{\mathrm{Fish}}\, \approx \, 2\, -\, 8\, \mathrm{cm/s}$, Appendix 4—figure 2). The effect of the flow-field was therefore neglected.

During the acclimation phase, the water tank is partitioned into 12 °C in the left compartment and 4 °C (shown is TR4) in the right compartment (Appendix 1—figure 1). After 15 min of acclimation, the central gate is removed (here at time: $t\, =\, 0$) and the density difference drives a lock exchange flow with the transparent warm water flowing slowly in the upper half of the tank to the right and the dyed cold water flowing along the channel bottom towards the left ($t\,=\,0.8$ min). After reaching the side walls of the tank, the directionality of the flow reverts and the current sloshes back and forth in the form of an internal wave ($t\,=\,1.8 - 5$ min). A clear second and a third wave passage take place, while flow speed and wave amplitude gradually attenuate through dissipation. During these internal wave passages, the vertical height of the temperature interface remains a periodic function of time. The start of the first treatment phase (p1) was defined as the time when the vertical gate was fully removed from the water ($t\, =\, 0$).

**Appendix 1—table 1.** Definition of treatments. We contrasted water at the acclimation temperature (12 °C) with four cold treatments (TR1–TR4) and four warm treatments (TR6–TR9); hence, we varied the incoming water temperature in both directions. Initial water temperature (T) and density (ρ) in the left (subscript: _left_) and right (subscript: _right_) compartments of the tank for each treatment. Treatment 5 (TR5) was the control treatment, conducted without the addition of dye (see Appendix 6).

Treatment	*T*_left_ [°C]	*T*_right_ [°C]	*ρ*_left_ [kg/m^3^]	*ρ*_right_ [kg/m^3^]	Density ratio: *γ* [-]
TR1	12	10	999.5	999.7026	0.99979734
TR2	12	8	999.5	999.8512	0.99964875
TR3	12	6	999.5	999.9432	0.99955677
TR4	12	4	999.5	999.9749	0.99952509
TR5 (control)	12	6	999.5	999.9432	0.99955677
TR6	12	14	999.5	999.2474	1.00025279
TR7	12	16	999.5	998.946	1.00055458
TR8	12	18	999.5	998.5986	1.00090266
TR9	12	20	999.5	998.2071	1.00129522

### Water parameters

Both water temperature and dissolved oxygen were continuously monitored during experimentation (Appendix 1—figures 2 and 3). Here, we show the obtained measurements for the cold and warm water treatments.

## Appendix 2

### Fish tracking

The following set of parameters was used for fish tracking in the software TRex (Appendix 2—table 1). Detailed description of parameters can be found here: https://trex.run/docs/parameters_trex.html

**Appendix 2—table 1.** Tracking parameters used for fish tracking in TRex software.

TGrabs parameters	TRex parameters
enable_difference = true reset_average = true use_adaptive_threshold = true adaptive_threshold_scale = 2 dilation_size = –3 track_max_individuals = 4 average_samples = 3,000 averaging_method = mean threshhold = 150 meta_real_width = 202 blob_size_ranges = [[0.1,6]] approximate_length_minutes = 15 cam_framerate = 24	blob_size_ranges = [[0.1,6]] track_max_reassign_time = 5 speed_extrapolation = 3 frame_rate = 24 gui_focus_group = [0,1,2,3] heatmap_ids = [0,1,2,3] track_max_reassign_time = 0.5 heatmap_resolution = 9 huge_timestamp_ends_segment = false midline_start_with_head = true midline_stiff_percentage = 0.4 outline_approximate = 15 output_csv_decimals = 2 output_posture_data = true output_heatmaps = true posture_closing_steps = 1 track_end_segment_for_speed = false track_max_speed = 40 track_max_individuals = 4 track_max_reassign_time = 5 blob_split_max_shrink = 0.2 output_invalid_value = nan track_ignore = [[[0,0],[0,16],[1840,0],[1840,16]]] auto_quit = true gui_auto_scale_focus_one = true

Thereby, the fish position was detected on average 99% of the time.

## Appendix 3

### Swimming trajectories

The following plots show the abstracted swimming trajectories for all fish in all treatments.

## Appendix 4

### Swimming speed

The fish's location at any time instance is represented by the center of gravity of its detected shape. Naturally, this location is displaced by short-term body rotations or body wobble, leading to unwanted artifacts in the trajectory which propagate into its derivatives (i.e. velocity and acceleration). To dampen these high-frequency fluctuations, we fitted a ‘moving-spline’ to the trajectory acting as a low-pass filter (Appendix 4—figure 1B, C and D) – an approach suggested by Lüthi et al., 2005. For each time step, t, and around each point of a measured trajectory, a third-order polynomial is fitted from t − 10 · ∆t to t+10· ∆t for each component, $x_{1}$, $x_{2}$ (*x*- and *y*-position). This results in a fit to 21 measured trajectory points. The constants, $c_{i}$, for the polynomial of type

$$\displaystyle x_{i}\left (t\right)=c_{i,0}+c_{i,1}t+c_{i,2}t^{2}+\ c_{i,3}t^{2}$$

are determined as

$$\displaystyle c_{i}=\left (\boldsymbol{A}^{T}\, x_{i}\right)\left (\boldsymbol{A}^{T}\, \boldsymbol{A}\right)^{-1}\ ,$$

where

$$\displaystyle \boldsymbol{A}=\, \left [\begin{array}{c}\begin{array}{cc}1 & \left (t-10\cdot \Delta t\right)\\1 & \left (t-9\cdot \Delta t\right)\end{array}\\\begin{array}{cc}\ldots & \ldots \\1 & \left (t+10\cdot \Delta t\right)\end{array}\end{array}\begin{array}{c}\begin{array}{cc} \left (t-10\cdot \Delta t\right)^{2} & \left (t-10\cdot \Delta t\right)^{3}\\\left (t-9\cdot \Delta t\right)^{2} & \left (t-9\cdot \Delta t\right)^{3}\end{array}\\\begin{array}{cc}\ldots & \ldots \\\ \left (t+10\cdot \Delta t\right)^{2} & \left (t+10\cdot \Delta t\right)^{3}\end{array}\end{array}\right ]$$

and

$$\displaystyle x_{i}=\, \left [\begin{array}{c}x_{i}\left (t-10\cdot \Delta t\right)\\x_{i}\left (t-9\cdot \Delta t\right)\\\ldots \\x_{i}\left (t+10\cdot \Delta t\right)\end{array}\right ]$$

The filtered position, velocity, and acceleration, $\hat{x_{i}}\left (t\right),\, \hat{u_{i}}\left (t\right)\, and\, \hat{a}_{i}\left (t\right)$, were then obtained per component as:

$$\displaystyle \hat{x}_{i}(t)=c_{i,0}+c_{i,1}(t)+c_{i,2}(t^{2})+c_{i,3}(t^{3}),$$

$$\displaystyle \hat {u}_{i}\left (t\right)=c_{i,1}+2c_{i,2}t+3c_{i,3}t^{2}$$

$$\displaystyle \hat {a}_{i}\left (t\right)=2c_{i,2}+6c_{i,3}t$$

the swimming speed $\hat {v}\left (t\right)$ was then derived as:

$$\displaystyle \hat {v}\left (t\right)=\, \sqrt{\left (\hat {u}_{1}\left (t\right)\right)^{2}+\left (\hat {u}_{2}\left (t\right)\right)^{2}}$$

Finally, an individuals’ normalized swimming speed $v_{norm}\left (t\right)$ is calculated by division through the individuals’ temporally averaged speed during the treatment phase, $\overline{\widehat{v} }$:

$$\displaystyle v_{norm}\left (t\right)\,=\, \frac{\hat {v}\left (t\right)}{\overline{\widehat {v}}}$$

The effect of the moving spline on the raw velocity components and the derivation of $\hat {v}\left (t\right)$ and $v_{norm}\left (t\right)$ is visualized in Appendix 4—figure 1A-E and in Appendix 13—Video 5.

After the velocity smoothing, the absolute swimming speed could be calculated.

**Appendix 4—table 1.** Shapiro-Wilk test for cold treatments. Group averaged and normed swimming speeds $\bar{\bar{{v}}}_{{n},{t}}$ for different temperatures (above and below interface).

	TR1		TR2		TR3		TR4	
Temperature [°C]	12	10	12	8	12	6	12	4
Replicate								
1	1.0726	0.9593	1.4859	1.1885	0.8875	1.4206	0.9857	1.0257
2	1.0231	0.9907	0.9691	1.1946	0.9896	1.2103	0.928	1.489
3	0.9545	1.1524	0.9309	1.4012	0.9202	1.1159	1.0319	1.0756
4	1.0015	0.9802	0.981	1.2883	0.9433	1.3381	0.9652	1.279
5	0.9436	1.0808	0.9747	1.1839	0.9841	1.3986	0.917	1.3978
p-value	0.714	0.297	**0.002**	0.079	0.612	0.465	0.743	0.576
test_stat	0.947	0.877	0.63	0.799	0.932	0.909	0.951	0.927

**Appendix 4—table 2.** Shapiro-Wilk test for warm treatments. Group averaged and normed swimming speeds for different temperatures (above and below interface).

	TR6		TR7		TR8		TR9	
Temperature [°C]	12	14	12	16	12	18	12	20
Replicate								
1	1.0107	1.0181	0.9482	1.0876	0.9654	1.0185	0.9535	1.1097
2	0.9723	1.027	0.8855	1.1742	1.1352	0.9216	0.959	1.0755
3	0.985	1.0255	0.9288	1.1218	0.8776	1.0737	0.9735	1.3444
4	0.9409	1.0696	0.9546	1.0416	0.8878	1.0693	0.9914	1.0356
5	0.9811	0.9994	0.8443	1.1643	0.9375	1.0068	1.0333	0.9775
p-value	0.839	0.396	0.409	0.678	0.15	0.361	0.387	0.247
test_stat	0.965	0.897	0.9	0.942	0.834	0.891	0.896	0.865

**Appendix 4—table 3.** t-test results for cold treatments. ns: not significant; *: $0.01\boldsymbol{\, }< \boldsymbol{\, }\boldsymbol{p}\boldsymbol{\, }\leq \boldsymbol{\, }0.05$; ***: $0.0001\boldsymbol{\, }< \boldsymbol{\, }\boldsymbol{p}\boldsymbol{\, }\leq \boldsymbol{\, }0.001$:

Treatment	T_top_ [°C]	T_bottom_ [°C]	p-Value	Significance
TR1	12	10	0.4603	ns
TR2	12	8	0.1438	ns
**TR3**	12	6	0.0004	***
**TR4**	12	4	0.014	*

**Appendix 4—table 4.** t-test results for warm treatments.

Treatment	Ttop [°C]	Tbottom [°C]	p-Value	Significance
TR6	14	12	0.0147	*
TR7	16	12	0.0002	***
TR8	18	12	0.3199	ns
TR9	20	12	0.0859	ns

## Appendix 5

### Vertical occupancy

The following plots display the time fraction that fish were spending in the upper and lower half of the tank.

**Appendix 5—table 1.** Friedman test within each treatment,<time-fraction spent in 12 °C >, Tr depicts the treatment temperature initially in the right compartment of the tank.

Treatment	T_r_ [°C]	Source	W	ddof1	Q	p-unc
**TR1**	10	Phase	0.76	2	7.6	**0.022***
TR2	8	Phase	0.04	2	0.4	0.819
**TR3**	6	Phase	0.76	2	7.6	**0.022***
TR4	4	Phase	0.48	2	4.8	0.091
TR6	14	Phase	0.76	2	7.6	0.094
**TR7**	16	Phase	0.84	2	8.4	**0.015***
TR8	18	Phase	0.52	2	5.2	0.074
TR9	20	Phase	0.28	2	2.8	0.247

**Appendix 5—table 2.** TR1, Dunn-test.

Phase	1	2	3
1	1.0	**0.04***	**0.049***
2		1.0	1.0
3			1.0

**Appendix 5—table 3.** TR3, Dunn-test.

Phase	1	2	3
1	1.0	0.774	0.688
2		1.0	1.0
3			1.0

**Appendix 5—table 4.** TR7, Dunn-test.

Phase	1	2	3
1	1.0	0.688	0.537
2		1.0	1.0
3			1.0

## Appendix 6

### Control experiment

To assess the effect of the blue dye, control experiments without the use of blue dye were conducted for TR3. Due to a camera failure, one of the control experiments could not be processed; hence, the control is based on four replicate*s*, while TR3 is based on five replicates. No significant difference with regard to the vertical occupancy was detected. Here, we show the 2D swimming trajectories and the vertical occupancy to allow for comparison (Appendix 6—figures 1 and 2).

## Appendix 7

### The thermal interface

The lateral view of the tank was recorded at 24 frames/s with a digital video camera (GoProHero7). The light was absorbed by the dye and hence, the intensity of the image could be related to the dye concentration along the light path from the back to the front of the tank (Shin et al., 2004). With this arrangement, the reduction in intensity is exponentially related to the dye concentration. Since the blue dye diffuses at about the same rate as (in both cases diffusion is negligible for this flow), the dye concentration is a surrogate for the density and therefore the water temperature.

On the left side, fish (tested in a group of four in each replicate) were acclimated (15 min) to water at 12 °C, which was identical to the housing temperature. On the right side, we added water at a different temperature (colder or warmer), dyed with 0.29 mg/L pre-dissolved *Patent blue V sodium salt* (C_27_H_31_N_2_NaO_7_S_2_, Sigma-Aldrich) to allow visualization of the thermal gradient. After gate removal, the water stratified vertically and the thermal interface could be derived based on image analysis.

Here, we followed a theoretical approach to simulate the thickness of the vertical gradient over time.

## Appendix 8

### Warm and cold water excursions

By combining the obtained swimming trajectories with the position of the thermal interface, warm- and cold-water excursions could be derived. Here, we show this procedure for two examples (Appendix 8—figure 1). Then we show all the obtained excursions for all experiments (Appendix 8—figures 2 and 3).

In a next step, the excursions were used as a behavioral metric. In particular, the number and depth of excursions was investigated (Appendix 8—figures 4 and 5).

A statistical analysis (Friedman test and Dunn test) of the number of excursions (Appendix 8—figure 4) revealed no significant reduction in the number of performed cold- and warm-water excursions over time.

**Appendix 8—table 1.** Friedman test, result, and median number of excursions over time for all treatments.

Treatment	*T*_r_ [°C]	Source	W	ddof1	Q	p-unc
**TR1**	10	Phase	0.760	2	7.600	**0.022**
TR2	8	Phase	0.443	2	4.429	0.109
TR3	6	Phase	0.200	2	2.000	0.368
TR4	4	Phase	0.178	2	1.778	0.411
TR6	14	Phase	0.074	2	0.737	0.692
TR7	16	Phase	0.040	2	0.400	0.819
TR8	18	Phase	0.520	2	5.200	0.074
**TR9**	20	Phase	0.640	2	6.400	**0.041**

**Appendix 8—table 2.** TR1, Dunn-test (post-hoc).

Phase	1	2	3
1	1.0	0.537	0.312
2		1.0	1.0
3			1.0

**Appendix 8—table 3.** TR9, Dunn-test (post-hoc).

Phase	1	2	3
1	1.0	1.0	0.308
2		1.0	1.0
3			1.0

To test whether the median number of cold-water excursions $\overset{\sim }{N}$ is normally distributed, we conducted a Shapiro-Wilk test for each of our treatments. The data of each treatment was confirmed to be normally distributed (Appendix 8—table 4) (i.e. p-value >0.05), and thus, a one-way ANOVA (python: scipy.stats.f_oneway) was applied to test for significant difference between the treatments (Appendix 8—table 5). To find specific differences, we then applied a t-test to see which treatments are significantly different (Appendix 8—table 6).

**Appendix 8—table 4.** Median number of cold-water excursions $\tilde {\rm N}$ of four fish tested simultaneously within each experimental replicate. Shapiro-Wilk-test results.

Replicate	TR1	TR2	TR3	TR4	TR6	TR7	TR8	TR9
1	40.5	2.5	18.0	21.5	56.5	48.5	61.0	70.5
2	61.0	20.5	24.5	24.5	61.5	46.5	44.5	66.5
3	50.0	31.0	34.5	28.0	65.5	51.5	60.0	68.5
4	69.0	11.5	43.0	24.0	62.5	51.0	75.0	97.0
5	34.5	32.5	27.5	16.0	70.0	43.5	67.5	87.0
test_stat	0.96	0.93	0.98	0.95	0.99	0.93	0.96	0.85
p-value	0.82	0.58	0.95	0.77	0.98	0.64	0.78	0.19

**Appendix 8—table 5.** One-way ANOVA test result.

f-Statistic	p-Value
21.5	2.02e-10

**Appendix 8—table 6.** Pairwise t-test result.

Pairs	p-Value
TR1, TR2	**0.0063**
TR1, TR3	**0.0230**
TR1, TR4	**0.0029**

For completeness, we also show the depth, duration, and number of warm-water excursions in the warm treatments.

**Appendix 8—table 7.** Percentage of all captured cold and warm excursions shorter than 10 s for all phases (p1, p2, and p3) of each treatment.

Treatment	Short cold excursions (*Ω* < 10 s) [%]	Short warm excursions (*Ω* < 10 s) [%]
TR1	75.4	69
**TR2**	**91.2**	**38.1**
**TR3**	**94**	**49.1**
**TR4**	**92.2**	**42.7**
TR6	81	76.3
TR7	73.1	71.4
TR8	72.5	82.7
TR9	88.2	81.6

## Appendix 9

### Vertical turning points

The vertical turning points were abstracted by analyzing the swimming trajectory of each individual fish. Here, we show this procedure and the outcome of this analysis.

**Appendix 9—table 1.** Mann–Kendall test. Input data: median distance $\tilde{\tilde{\boldsymbol{D}}}_{\max}$ (in centimeters) and median durations $\tilde {\tilde {{\Omega }}}$ (in seconds) across all treatments and experimental replicates.

Treatment	*T*_bottom_ [°C]	$\tilde {\tilde {D}}_\text {max}$ [cm]	Med($\tilde {\tilde {D}}_\text {max}$)	$\tilde {\tilde {\Omega}}$ [s]	Med($\tilde {\tilde {\Omega}}$)
TR1	10	10.87		7.02	
	10	9.83		5.45	
	10	9.50		6.04	
	10	10.37		4.10	
	10	9.91	9.91	6.94	6.04
TR2	8	14.52		276.22	
	8	5.79		4.04	
	8	7.38		3.94	
	8	6.31		4.30	
	8	4.97	6.31	2.88	4.04
TR3	6	4.86		3.25	
	6	4.97		3.23	
	6	5.98		3.48	
	6	3.90		2.56	
	6	5.00	4.97	2.79	3.23
TR4	4	4.14		2.84	
	4	2.94		2.19	
	4	5.27		2.63	
	4	5.24		2.59	
	4	4.28	4.28	1.95	2.59

**Appendix 9—table 2.** Mann–Kendall test result. A p-value lower than some significance level (common choices are 0.10, 0.05, and 0.01) indicates statistically significant evidence that a trend is present in the data. We found in both cases p < 0.001.

Test results	(*T*_bottom_,$\tilde {\tilde {\Omega}}$)	(*T*_bottom_,$\tilde {\tilde {D}}_\rm {max}$)
Trend	Decreasing	Decreasing
**p-value**	5.71E-07	4.27E-06
Test statistic	–292	–263
z-value	-5	–4.60
tau-value	–0.77	–0.69
s-value	–292	–263.00
Slope	–0.30	–0.36
Intercept	7.85	9.55

## Appendix 10

### Burst swimming speed

We compared our observed maximum swimming speeds to a multivariate swimming speed model for rheophilic fish species (Ebel, 2014). Thereby, fish burst swimming speed (u) was calculated for a duration of $t\, =\, 5$ s, body length of $BL\, =\, 0.036\, \mathrm{m}$, and temperatures of $T\, =\, {12\,}^{\circ} {\rm C}$ and $T\, =\, {6\,}^{\circ}{\text {C}}$ (Appendix 10—figure 1).

$$\displaystyle \log \left (u\right)=0,5460+ 0,7937 \cdot \log \left (BL\right)\, -0,0902 \cdot \log \left (t\right)\, + 0,2813 \cdot \log \left (T\right)$$

The direct comparison of our observed absolute swimming speeds to the model is, however, difficult because:

1. The model is based on swimming speed derived from respirometers where a steady flow is present. The absence of flow in our experiments does not compare to this, and fish inherently display lower swimming speeds.
2. The confined space and the presence of walls force fish to decelerate frequently, preventing them from reaching very high swimming speeds.
3. We are looking at the 2D-projection of the swimming trajectory; thus, our observations neglect the third velocity component.

## Appendix 11

### Swimming speed vs. excursion duration

Our analysis revealed how swimming speed and duration of excursions reveal distinctive patterns in cold and warm water treatments. We found clear clusters with respect to swimming speed (y-axis) and duration (x-axis; Appendix 11—figure 1).

## Appendix 12

### Simulated body cooling

**Appendix 12—table 1.** Input to body-cooling model. Summary of fish body temperature at the beginning of cold-water excursion (*T*_b_) and ambient water temperature (*T*_a_) during cold-water excursions.

Treatment	*T*_b_ [°C]	*T*_a_ [°C]
1	12	10
2	12	8
3	12	6
4	12	4

## Appendix 13

### Supplementary Movies

All movies are played at 20 x real speed. The original frame rate was 24 frames/s.

Appendix 13—video 1. Tracking of 1 fish in cold treatment (TR2). Side-view of the tank with individual fish swimming-trajectory and gravity current for single experimental replicate (TR2, cold-water in light blue). Individual fish movement trajectory is shown in red and its instantaneous vertical distance to the thermal interface $D\left (t\right)$ in the lower plot for the progression of the entire treatment (p1, p2, and p3) immediately after gate-removal. Playback is 20 x real speed.

Appendix 13—video 2. Tracking of four fish in cold treatment (TR2). Same for all four individuals.

Appendix 13—video 3. Tracking of one fish in warm treatment (TR7). Side-view of the tank with individual fish swimming-trajectory and gravity current for single experimental replicate (TR7, warmer water in light blue). Individual fish movement trajectory is shown in red and its instantaneous vertical distance to the thermal interface $D\left (t\right)$ in the lower plot for the progression of the entire treatment (p1, p2, and p3) immediately after gate-removal. Playback is 20 x real speed.

Appendix 13—video 4. Tracking of four fish in warm treatment (TR7). Same for all individuals.

Appendix 13—video 5. Derivation of normed swimming speed. Derivation of normed swimming speed for trajectory segment of 60 seconds. (A) Single fish trajectory. (B) and (C) horizontal and vertical velocity components. (D) Absolute swimming speed. (E) Normed swimming speed.
