# Supplementary material for: Salmonids elicit an acute behavioral response to heterothermal environments
Source: eLife. 2026 Oct 14;13:RP99126. doi: 10.7554/eLife.99126 (PMC13668120; doi:10.7554/eLife.99126)
Supplement: Peer review history

# Editor report: eLife Assessment

**DOI:** 10.7554/eLife.99126.3.sa0

**Authors:** Gordon J Berman

**Affiliations:** Emory University United States

**Keywords:** Solid, Valuable

This **valuable** paper investigates how fish avoid thermal disturbances that occur on fast timescales. The authors use a creative experimental approach that quickly creates a vertical thermal interface, which they combine with careful behavioral analyses. The evidence supporting their results is **solid**, but there is a potential confounding factor between temperature and vertical positioning, and characterization of the thermal interface would greatly assist in interpreting the results.

# Referee report: Reviewer #1 (Public review)

**DOI:** 10.7554/eLife.99126.3.sa1

Summary:

The experiment is interesting and well executed and describes in high detail fish behaviour in thermally stratified waters. The evidence is strong but the experimental design cannot distinguish between temperature and vertical position of the treatments.

Strengths:

High statistical power, solid quantification of behaviour.

Weaknesses:

A major issue with the experimental design is the vertical component of the experiment. Many thermal preference and avoidance experiments are run using horizontal division in shuttlebox systems or in annular choice flumes. These remove the vertical stratification component so that hot and cold can be compared equally, without the vertical layering as a confounding factor. The method chosen, with its vertical stratification, is inherently unable to control for this effect because warm water is always above, and cold water is always below. This complicates the interpretations.

# Referee report: Reviewer #2 (Public review)

**DOI:** 10.7554/eLife.99126.3.sa2

The paper by Naudascher et al., investigates an interesting question: How do fish react to and avoid thermal disturbances from the optimum that occur on fast timescales. Previous work has identified potential strategies of warm avoidance in fish on short timescales while strategies for cold avoidance are far more elusive. The work combines a clever experimental paradigm with careful analysis to show that trout parr avoid cold water by limiting excursions across a warm-cold thermal interface. While direct measurements of the interface are lacking, thermal dynamics simulations suggest that trout parr avoid the warm-cold interface in the absence of gradient information.

The authors assume that the thermal interface triggers the upward turning behavior, possibly leading to the formation of an associative memory. However, an alternative explanation is that exposure to cold water during initial excursions increases the tendency for upward turns. In other words, exposure to a cold interface changes the behavioral state leading to increases in gravity controlled upward turning. This could be an adaptive strategy since for temperatures > 4C swimming upwards is a good strategy to reach warmer water. That being said, the vertical design offers new insight and is ecologically relevant.

# Author comment: Author response

**DOI:** 10.7554/eLife.99126.3.sa3

**Authors:** Robert Naudascher, Stefano Brizzolara, Jonasz Slomka, Robert M Boes, Markus Holzner, Luiz GM Silva, Roman Stocker

**Affiliations:** ETH Zurich Zurich Austria; ETH Zurich Zurich Switzerland; ETH Zurich Zürich Switzerland; ETH Zurich Zürich Switzerland; Swiss Federal Institute for Forest, Snow and Landscape Research Zürich Switzerland; ETH Zurich Zürich Switzerland; ETH Zurich Zurich Switzerland

The following is the authors’ response to the original reviews

**Reviewer #1**:

Summary:

The experiment is interesting and well executed and describes in high detail fish behaviour in thermally stratified waters. The evidence is strong but the experimental design cannot distinguish between temperature and vertical position of the treatments.

Strengths:

High statistical power, solid quantification of behaviour.

Weaknesses:

A major issue with the experimental design is the vertical component of the experiment. Many thermal preference and avoidance experiments are run using horizontal division in shuttlebox systems or in annular choice flumes. These remove the vertical stratification component so that hot and cold can be compared equally, without the vertical layering as a confounding factor. The method chosen, with its vertical stratification, is inherently unable to control for this effect because warm water is always above, and cold water is always below. This complicates the interpretations and makes firm conclusions about thermal behaviour difficult.

We highly appreciate this evaluation and have addressed the reviewer’s specific comments below.

The sentence "Further, the metabolic performance (and thus functions including growth, reproduction, and locomotion) of ectotherms takes the form of a bell-shaped curve as a function of temperature6, peaking within a range of optimal temperatures (the 'preferendum') and going to zero at lower and upper temperature limits7." contains several over-simplifications and misconceptions:

(1) Thermal performance curves are never bell-shaped.

(2) The optimum for various traits often shows different TPCs.

(3) The preferendum rarely lines up with the thermal optimum for various trait TPCs.

(4) Performance for various traits rarely reaches zero at upper or lower limits, instead they can reach zero at less extreme temperatures (e.g. growth) or maintain high function all the way up to and sometimes beyond thermal limits (e.g. aerobic scope, heart rate).

We highly appreciate this input. We have replaced that sentence with: L69-71: “Because temperature influences the rates of most physiological processes, rapid warming or cooling can affect fish performance traits, including metabolic rates, swimming ability, and thermal tolerance (Jutfelt et al. 2024).”

The use of adaptation instead of acclimation is confusing. Adaptation should be reserved for evolutionary change. This is an issue in several parts of the manuscript.

Thanks for this input, we have replaced the word adapt with acclimate in two instances: L79 and L398.

It is not true that "very few quantitative studies of thermotaxis have been conducted in fish". There exists an extensive literature on thermal preference and avoidance in fish that the manuscript downplays.

Thanks a lot for this input. We understand that thermal preference is ultimately driven by mechanistic responses to thermal gradients, and that thermotaxis and thermokinesis are the two mechanisms used by fish to navigate heterothermal environments. Our study and analysis are focused on understanding these mechanisms in vertically stratified conditions, not to understand thermal preferences per se. We have modified our text to clarify this aspect. Our literature review was focused on the behavioral mechanisms and our understanding is that the establishment of thermal preferences has a different goal compared to understanding how fish respond to rapid changes in water temperature. We have deleted that sentence and replaced it by (L107-110): “While the thermal preference of fish is a well-established field of research, very few quantitative studies of the behavioral mechanisms allowing fish to seek their preferendum (i.e. thermotaxis) have been conducted in fish.”

(Methods) It is unclear why the blue dye was used in all experiments. The fish can see the differently coloured water layer and that may have affected their choices. Five control trials without dye were run but finding no difference there could also be due to low statistical power.

We appreciate this comment. The blue dye was used to visualize the precise location of the thermal interface and was therefore necessary in all experiments (see Methods section ‘Visualization and evolution of the thermal interface’). We acknowledge that fish can perceive the colored water layer, but since the dye concentration and resulting color intensity were consistent across all treatments, we do not see how it could have acted as a confounding variable. While we recognize the possibility of some behavioral influence from the dye, the clear behavioral differences across treatments indicate that it was not a determining factor. To emphasize this we have added the following to the manuscript (L701-703): “Furthermore, because the dye concentration and resulting color intensity were consistent across all treatments, the dye did not act as a confounding variable in our statistical comparisons.”

Regarding statistical power, our control experiment without dye (N = 16 fish, 4 replicates; see Fig. S34 and S35) provides sufficient statistical power to assess whether the dye influenced behavior. The reviewer indicated that the high statistical power was a strength of the paper, which aligns with our view that our study design enables robust statistical comparisons. It seems contradictory that statistical power is a concern for the control trials, given that our main experiments were conducted with a similar sample size. Indeed, the number of replicates used is consistent with similar studies and balances statistical rigor with the ethical goal of reducing the number of animals used in experimentation. To emphasize this, we have added the following to the manuscript (L865-868): “The number of replicates used in this study reflects a balance between statistical rigor and the ethical imperative to minimize the use of animals in experimentation. Regarding statistical power, our design (five replicates with groups of four fish each) is consistent with similar studies and represents an adequate sample size.”

A major issue with the experimental design is the vertical component of the experiment. Many thermal preference and avoidance experiments are run using horizontal division in shuttlebox systems or in annular choice flumes. These remove the vertical stratification component so that hot and cold can be compared equally, without the vertical layering as a confounding factor. The method chosen, with its vertical stratification, is inherently unable to control for this effect because warm water is always above, and cold water is always below. This complicates the interpretations and makes firm conclusions about thermal behaviour difficult. This issue should be thoroughly discussed.

Thank you very much for this comment. We revised the manuscript accordingly, to clearly indicate that our goal was to assess the response of fish to vertically thermally stratified water, a scenario that occurs frequently in nature. We have added the following paragraph the discussion (L523-530): “However, a generalization of our observations to horizontally oriented thermal gradients remains elusive. Our results are inherently tied to the vertical stratification created in our experiments. As warm water was always positioned above and cold water below, we could not control for the effect of vertical position (i.e., we could not do cold over warm layer experiments). This limits our ability to directly compare our findings to those obtained from horizontally oriented thermal gradients. On the other hand, the case we addressed is of direct environmental relevance, as natural waters often experience vertical thermal stratification.”

It is unclear why the authors assume an "optimal temperature" (undefined for which trait) of 12°C for brown trout parr, and why they assume the preference temperature would match that "optimal" temperature. The thermal biology for any fish species is more complex than a single perfect temperature, with various traits showing differing optima and often a mismatch with the preferred temperature. The literature suggests brown trout growth optima between 13 and 16°C, and preference temperature has even been suggested to be as high as 21°C. In light of this, the authors' conclusion that brown trout avoid cold and don't avoid warm water is possibly misguided. It is possible that the brown trout had a preference temperature higher than 12°C, which should be acknowledged and discussed.

This is indeed a very important aspect, which was partly (but indeed not fully) already addressed in the discussion. To reflect these considerations, we have expanded the existing paragraph in the discussion (additions are in yellow). (L422 - L439): “We conclude from the behavior of fish when warmer water was available that their acute thermal preferendum exceeded 12 °C, departing from the acclimation temperature we had chosen based on the thermal preferendum for trout reported in literature[33]. Indeed, the thermal biology for any fish species is more complex than a single, static thermal preferendum: Many internal and external factors, such as hypoxia, satiation, time of day, and life stage[5], can influence the temperature preference of fish. For example, the level of satiation can have an impact because when fish are well fed, their growth rate increases with body temperature as metabolic performance increases[40]. This modifies the preferred temperature, as observed in Bear Lake sculpin (*Cottus extensus*) that ascend into warmer water after feeding to stimulate digestion and thereby achieve a three-fold higher growth rate[41]. In contrast, field studies with adult fish have observed movement from warm to cold water in summer[42,43], allowing fish to lower their metabolic rate, likely in effort to conserve energy[2,44]. We propose that the behavior of trout parr upon exposure to warmer water in our experiments served to achieve a higher body temperature to ultimately increase growth rate, which is critical for this life stage[45,46]. Indeed, growth experiments on brown trout populations have shown that optimal growth temperatures can range between 15 and 19 °C, depending on the stream of origin[46].”

The figures are unnecessarily complex and introduce a long list of abbreviations and Greek characters for no apparent reason. There are many simpler ways for showing the results so unclear why they are so opaque.

We appreciate the reviewer’s feedback and agree on the importance of clarity, however (in the absence of specific suggestions) we did not make changes to the figures or the use of Greek characters (which align with convention), as we believe they effectively convey the results. We highlight that the data themselves are very rich (multiple fish, multiple phases, multiple treatments, etc.) and we wanted to convey this richness in a compact and transparent manner.

**Reviewer #2:**

This paper investigates an interesting question: how do fish react to and avoid thermal disturbances from the optimum that occur on fast timescales? Previous work has identified potential strategies for warm avoidance in fish on short timescales while strategies for cold avoidance are far more elusive. The work combines a clever experimental paradigm with careful analysis to show that trout parr avoid cold water by limiting excursions across a warm-cold thermal interface. While I found the paper interesting and convincing overall, there are a few omissions and choices in the presentation that limit interpretability and clarity.

A main question concerns the thermal interface itself. The authors track this interface using a blue dye that is mixed in with either colder or warmer water before a gate is opened that leads to gravitational flow overlaying the two water temperatures. The dye likely allows to identify convective currents which could lead to rapid mixing of water temperatures. However, it is less clear whether it accurately reflects thermal diffusion. This is problematic as the authors identify upward turning behavior around the interface which appears to be the behavioral strategy for avoiding cold water but not warm water. Without knowing the extent of the gradient across the interface, it is hard to know what the fish are sensing. The authors appear to treat the interface as essentially static, leading them to the conclusion that turning away before the interface is reached is likely related to associative learning. However, thermal diffusion could very likely create a gradient across centimeters which is used as a cue by the fish to initiate the turn. In an ideal world, the authors would use a thermal camera to track the relationship between temperature and the dye interface. Absent that, the simulation that is mentioned in passing in the methods section should be discussed in detail in the main text, and results should be displayed in Figure 1. Error metrics on the parameters used in the simulation could then be used to identify turns in subsequent figures that likely are or aren't affected by a gradient formed across the interface.

The authors assume that the thermal interface triggers the upward-turning behavior. However, an alternative explanation, which should be discussed, is that cold water increases the tendency for upward turns. This could be an adaptive strategy since for temperatures > 4C turning swimming upwards is likely a good strategy to reach warmer water.

The paper currently also suffers from a lack of clarity which is largely created by figure organization. Four main and 38 supplemental figures are very unusual. I give some specific recommendations below but the authors should decide which data is truly supplemental, versus supporting important points made in the paper itself. There also appear to be supplemental figures that are never referenced in the text which makes traversing the supplements unnecessarily tedious.

The N that was used as the basis for statistical tests and plots should be identified in the figures to improve interpretability. To improve rigor, the experimental procedures should be expanded.

Specifically, the paper uses two thermal models which are not detailed at all in the methods section.

We appreciate these crucial comments to our paper. We have addressed these points in detail below.

As stated above, a characterization of the thermal interface is critical. Ideally via measurement or at least by expanding on the simulation.

We appreciate the idea of using thermal cameras and, indeed, we had initially tried to use them. However, thermal cameras generally cannot see through plexiglass or glass-like material due to the way infrared radiation interacts with these materials. While thin plastics can transmit some infrared, thicker plastics and reflective materials like glass tend to block or reflect infrared light.

We have attempted to better characterize the thermal interface thickness, namely the spatial extent of the thermal gradient over the time period of our experiments (20 min). Indeed, our simulations in the original SI were conducted precisely to estimate the thermal interface thickness, though based on thermal diffusion in still water, while turbulence generated by the moving gravity current can smear out the interface, particularly in the initial phase. To account for this in our in the reviewed manuscript, we adopted a phenomenological approach to estimate the initial increase in thickness of the thermal interface due to turbulence and present this refined simulation in our manuscript.

Our analysis suggests that, rather than assuming an initial interface thickness of zero (as in the original version of the manuscript), the thermal diffusion simulations should begin with an initial thickness of 2.8 mm in TR1. To incorporate this adjustment, we set the initial interface thickness to 2.8 mm and ran the simulation forward for t = 20 min, assuming diffusion. This approach resulted in a final interface thickness ranging between 4 and 6 cm (see Fig. 29 in the Supplementary Information).

To reflect this refinement, we have added a new paragraph L717-758: "Characterization of the thermal gradient", to the Methods section. Additionally, we have updated Fig. S29 in the Supplementary Information and included an average (over time and across treatments) gradient thickness of 5 cm in Figs. 2 and 3 of the manuscript. The revised Figs. 2 and 3 now explicitly indicate the estimated vertical extent of the thermal gradient, with an extended caption detailing these changes.

The simulation should be detailed in the methods so that its validity can be evaluated and ideally, it should involve curved interfaces as encountered in the experiment.

To account for the effect of turbulence during the initial, inertia-dominated phase after the gate removal, we have provided a correction for the initial thickness of the interface (see the addition to the Methods section). Thank you for your suggestion regarding the incorporation of curved interfaces in the simulations. We believe that including curved interfaces in the simulations would not significantly affect the results. As shown in the manuscript, the interface is curved primarily during the initial phase of the process (first 2 min where the flow is inertia-dominated), which is currently not included in our data analysis (phase 1 begins 2 min after the gate removal).

In that vein, distances from the interface rather than height above the interface should be reported for the fish.

We acknowledge the reviewer’s suggestion to report distances from the interface rather than height above or below it. However, beyond the initial phase, we do not see a strong justification for using the orthogonal distance over the vertical distance, as the choice is inherently arbitrary (e.g., one could also measure the distance along the fish’s orientation vector). We have therefore kept our assessment based on the vertical distance.

Absent measurements, the paragraph on associative learning should be struck from the discussion as it is purely speculative.

We agree that the original paragraph on associative learning may have sounded overly speculative. However, after updating our manuscript with additional simulations of the thermal gradient's vertical extent, we found that fish perform upward turns not only above the thermal interface, but also before entering the thermal gradient itself. This observation makes us hesitant to attribute the response solely to thermotaxis. We believe it is essential to provide a plausible explanation—albeit speculative—for how fish initiate these turns before directly encountering the cold-water gradient. To support this, we have extended the discussion in this paragraph and added Supplementary Fig. 39. The new text now reads (additions in yellow): (L487 – 499): “Our findings show that fish were able to perform upward turns while still located above the thermal interface and that is, before actually sampling the cold water below the interface. In fact, our simulation of the vertical extent of the thermal gradient revealed that a substantial fraction of upward turns occurred before fish encountered the gradient itself — that is, prior to any sensory detection of the temperature change (Supplementary Fig. 39). This finding may be evidence of associative learning, whereby fish used information regarding the presence of colder water at depth obtained at prior times. While the current data do not provide conclusive evidence in this regard, they prompt the possibility that, rather than responding solely to immediate thermal cues, fish use spatial memory or associative learning to anticipate the location of colder water based on prior experience. Indeed, fish are able to perform associative learning based on non-visual cues[53], create mental maps of their surroundings54 and retain memory for hours[55], days[56] and months[57,58].”

The body-temperature simulations need to be detailed in the methods.

Thanks for this comment. We have removed the supplementary text section and have included the paragraph “Body cooling during cold-water excursions” into the methods section of our manuscript (L804 - L829).

Constant temperature experiments could be helpful in addressing the importance of a gradient/interface for triggering upward turning

We agree, however, we were limited (for ethical reasons) to a maximum number of fish we could use in the experiments. Hence, we focused on getting approval to run experiments focused on the responses to thermal gradients. However, occupancy during the acclimation phase in 12 °C showed that fish were much more stationary and primarily occupied the lower half of the tank.

A lot of ease of reading could be gained by labeling the conditions according to either the second temperature or perhaps even better the delta temperature (i.e. TR[-2C] instead of TR1).

We agree that labeling conditions by the second temperature or delta temperature could in principle improve readability. However, since T_bottom and T_top are explicitly mentioned in each main figure at least once, they can be directly associated with the respective treatment. Therefore, we have opted to retain the current labeling for consistency.

The figure legends are often short and do not accurately label all figure elements. This is especially true for supplemental figure legends which often appear rushed (e.g., the legend for Figure S2 stops mid-sentence, the legend of Figure S3 does not indicate what Ttop or Tbottom are).

We appreciate the reviewer’s comment and have carefully revised all figure legends to ensure clarity and completeness. Specifically, we have corrected figure labels, expanded the descriptions for supplemental figures, and ensured that all elements are accurately defined. For instance, we have completed the legend for Figure S2 and clarified the definitions of T_top and T_bottom in Figure S3. Additionally, we have systematically reviewed all figure legends to prevent inconsistencies and omissions.

For Figure S3, to improve clarity, plotting the standard deviation at different points in the tank across the phases could be more informative than the hard-to-distinguish multi-line plots in different shades of red.

We appreciate the reviewer’s suggestion regarding Figure S3. However, the primary goal of this figure is to illustrate how the thermal interface moves over time. While plotting the standard deviation at different points in the tank could provide additional statistical insights, it would detract from the intended visualization of the interface dynamics. For this reason, we have opted to retain the current multi-line representation. Nevertheless, we have ensured that the figure is as clear as possible by refining the color contrast and improving the legend for better readability.

There is an inconsistency in in-text citation styles (mixture of superscript and numbers in brackets).

Thank you for pointing this out. We have carefully reviewed the manuscript and corrected any inconsistencies in the in-text citation style to ensure uniform formatting throughout.

While the statement in the introduction, that increases in movement frequency could be purely metabolic in nature is correct, at least for larval zebrafish it has been shown that sensory neural activity is predictive of motor neuron activity and swim rates (Haesemeyer, 2018, cited by the authors).

This is an interesting finding. It is however unclear to us why this information is crucial in our context of brown trout parr.

Examples of summary results from Supplementary Figures 8-10 should be bundled in a main text figure since this appears to be important information supporting the conclusions.

We agree that Supplementary Figures 8–10 contain important information (i.e. Boxplots) on vertical occupancy and the time individuals spent in different water temperatures. However, this information is already integrated into Figure 2C, D, F, and G, which display the vertical distributions of fish across treatments and over time. Given the current length of the manuscript, adding another main-text figure could dilute rather than enhance clarity. For this reason, we have opted to keep these details in the Supplementary Materials while ensuring they are appropriately referenced in the main text.

The distributions of excursion length for all treatments should be graphed in a main figure to support the point made in the third paragraph of the "Trout parr... do not avoid warm water" section of the results.

We appreciate the reviewer’s suggestion. However, we do not believe that plotting excursion length is necessary to support this statement, as the key finding is already well represented in the manuscript. Specifically, the transition to bimodal depth occupancy, with fish spending comparable time above and below the interface in warm-water treatments (TR6–TR9), is clearly conveyed in Figure 2F and Supplementary Figure 8B. Additionally, this information is explicitly stated in the results section (L235): "Fish did not avoid warmer water in any of the warm-water treatments (TR6–TR9). Instead, fish transitioned to a bimodal depth occupancy, with comparable time spent above and below the interface (Fig. 2F; Supplementary Fig. 8B)." Given this, we believe that adding an additional figure would not enhance clarity but may instead introduce redundancy.

There should be a main figure panel that statistically compares the turn biases around the interface for the different conditions and the +/- 5cm interface line mentioned in the text should be visualized in the appropriate figures - incidentally, this length scale is on par with the diffusion seen in simulations further suggesting that fish in fact sense a gradient here rather than remembering an interface.

To address the reviewer’s comment, we have made the following updates:

• Extended and incorporated simulations of the thermal interface thickness (see Methods and Supplementary Fig. 29).

• Plotted the vertical locations of up-turning events relative to the phase-averaged position of the thermal interface (see Supplementary Fig. 39), which includes the estimated 5 cm vertical extent of the thermal gradient.

• Added the thermal interface thickness to the main figures (Fig. 3F,G and Fig. 2E,H) where applicable.

While we do not claim that memory alone explains cold-water avoidance, our data still suggests that it may contribute to the observed behavior, particularly since a substantial number of upturns occurred before the fish entered the thermal gradient (see also Author response image 1 below). Our aim is not to statistically disentangle the relative contribution of thermotaxis versus associative learning, but to propose a plausible interpretation of this observed anticipatory behavior with due caution to clarify that this is only a possibility.

Given that the thermal gradient is now visualized and characterized in detail, we respectfully suggest that an additional statistical comparison of turn biases would not add further clarity. We believe that is is evidence that vertical turning, away from the cold, occurred within and above the thermal gradient. However, we welcome the reviewer’s perspective and to demonstrate that turning points occur outside and above the thermal interface we have plotted them against gradient growth over time (see Author response image 1 below).

**Author response image 1.** The colored area indicates the temporal growth of thermal interface thickness.

**Reviewer #3:**

In this study, the authors measured the behavioural responses of brown trout to the sudden availability of a choice between thermal environments. The data clearly show that these fish avoid colder temperatures than the acclimation condition, but generally have no preference between the acclimation condition or warmer water (though I think the speculation that the fish are slowly warming up is interesting). Further, the evidence is compelling that avoidance of cold water is a combination of thermotaxis and thermokinesis. This is a clever experimental approach and the results are novel, interesting, and have clear biological implications as the authors discuss. I also commend the team for an extremely robust, transparent, and clear explanation of the experimental design and analytical decisions. The supplemental material is very helpful for understanding many of the methodological nuances, though I admit that I found it overwhelming at times and wonder if it could be pruned slightly to increase readability. Overall, I think the conclusions are generally well-supported by the data, and I have no major concerns.

Minor comments

P2 intro paragraphs 1/3 - it is not clear that thermal preference generally reflects the thermal optimum, partly because it is not clear what trait is being optimized (fitness?). Some nuance here would be helpful, and would also link nicely to the discussion on p10.

Thank you for this comment. We have now refined this section as follows (L67–71): "As most fish species are ectotherms, their body temperature fluctuates with the surrounding water temperature. Because temperature influences the rates of most physiological processes, rapid warming or cooling can affect fish performance traits, including metabolic rates, swimming ability, and thermal tolerance[6]."

To further clarify how thermal preference relates to thermal optimum and what trait is being optimized, we have incorporated additional nuance in this section. Specifically, we now acknowledge that thermal preference may not always align with the thermal optimum for performance or fitness.

P2 intro paragraph 2 - "adapt physiologically" implies evolution, but here you are referring to plasticity. Suggest saving the word "adapt/adaptation" for evolutionary changes (see also p9).

Thank you for this comment. We have revised the wording to "acclimate physiologically" (L79) to more accurately reflect plastic responses rather than evolutionary adaptation.

P7 - "This difference in probabilities (ρup - ρdown) was particularly large in the region immediately above and below the interface (-5 cm < D < 5 cm; Fig. 3F) and is a hallmark of a thermotactic behavior." I agree that the result provides compelling evidence for thermotaxis, but would it be possible to bolster this case by statistically testing for a difference in probabilities among the treatment groups here?

In addition to Fig. 3F, we are presenting statistical evidence that for colder water temperatures, fish penetrate less deeply into the cold lower water. The decreasing trend was statistically significant (Mann–Kendall test: $D_{{\rm max }}^{s}$, *p* < 0.001; Supplementary Table 6) and is presented in Fig. 4C. The depth reached during each cold-water excursion is determined by the location of the vertical turning point, which redirects the fish upward toward the surface. We think this is sufficient evidence for thermotaxis.

P9 paragraph 3 = "recent studies suggest that fish may instead respond to temporal changes of their internal body temperature." It seems like a citation is missing here. Would be useful to briefly summarize the evidence for internal temperature sensing that is the basis of this modelling exercise.

Thanks, we have added that citation (L385).

P10 "Our findings provide the first experimental evidence for this mode of behavioral thermoregulation in which fish navigate their heterothermal environment to achieve gradual body warming."

I think this statement overreaches given the presented data. While there may be a trend towards fish in the warm treatment spending increasing amounts of time in the upper half of the tank, I do not see this pattern supported statistically. There is also no evidence of gradual body warming, and even if there was I disagree that this would constitute experimental evidence that this was happening "intentionally". By this reasoning, any shuttlebox experiment in which fish actively shuttle between relatively warm and cool sides to end up with a preference that is above the starting condition would also constitute evidence for gradual warming. Overall, this is an interesting pattern, but I do not think there is sufficient evidence to conclude that fish are strategically warming.

We appreciate the reviewer’s comment and acknowledge that our original wording may have overstated the evidence. We have revised the sentence to better reflect the evdience presented (L411-415): “Our observations resemble this mode of behavioral thermoregulation, in which fish progressively favor warmer regions within a heterothermal environment. However, additional experimental evidence is required to determine the mechanisms underlying this behavior.”

P11 "Despite the avoidance response of cold water, fish engaged in repeated cold-water excursions..."

This is an interesting speculation, but I think it would be helpful to also point out that these fish are biased towards the bottom of the tank (based on control measurements) and this pattern may therefore simply reflect a desire to be lower in the water column.

Thank you for this helpful comment. We have now added this point to the revised text, which reads (L475-477): “Despite the avoidance response to cold water, fish engaged in repeated cold-water excursions, potentially reflecting a behavioral strategy to map the thermal environment. This pattern may also reflect an inherent tendency to occupy the lower part of the tank, as observed during homogeneous temperature of 12 °C during the acclimation phase.”

P13 - why was the dye always added to the right side of the tank, instead of being assigned to a side randomly? I think the control experiment is good evidence that the dye did not substantially affect behaviour, but it seems like it would have been nice to separate dye and novel temperature exposure.

We agree that randomizing the side of dye application would have been ideal. The dye was consistently added to the right side to maintain procedural consistency, ensuring that the “incoming” or “novel” temperature was always dyed. That said, our control experiment provides strong evidence that the dye itself did not influence behavior (as discussed above and in the manuscript).
